# The effectiveness of knowledge translation interventions for promoting evidence-informed decision-making among nurses in tertiary care: a systematic review and meta-analysis

**DOI:** 10.1186/s13012-015-0286-1

**Published:** 2015-07-14

**Authors:** Jennifer Yost, Rebecca Ganann, David Thompson, Fazila Aloweni, Kristine Newman, Afeez Hazzan, Ann McKibbon, Maureen Dobbins, Donna Ciliska

**Affiliations:** 1 School of Nursing, Faculty of Health Sciences, McMaster University, Main Street West, Hamilton, ON Canada; 2 School of Nursing, Faculty of Health and Behavioural Sciences, Lakehead University, 955 Oliver Road, Thunder Bay, ON Canada; 3 Singapore General Hospital, 31 Third Hospital Avenue, Singapore, Singapore; 4 Daphne Cockwell School of Nursing, Faculty of Community Services, Ryerson University, 350 Victoria Street, Toronto, ON Canada; 5 Department of Medicine, McMaster University, St. Peter’s Hospital-Hamilton Health Sciences, 88 Maplewood Avenue, Hamilton, ON Canada; 6 Clinical Epidemiology and Biostatistics, Faculty of Health Sciences, McMaster University, Main Street West, Hamilton, ON Canada

**Keywords:** Knowledge translation, Evidence-informed decision-making, Nursing practice, Tertiary care, Systematic review, Meta-analysis

## Abstract

**Background:**

Nurses are increasingly expected to engage in evidence-informed decision-making (EIDM) to improve client and system outcomes. Despite an improved awareness about EIDM, there is a lack of use of research evidence and understanding about the effectiveness of interventions to promote EIDM. This project aimed to discover if knowledge translation (KT) interventions directed to nurses in tertiary care are effective for improving EIDM knowledge, skills, behaviours, and, as a result, client outcomes. It also sought to understand contextual factors that affect the impact of such interventions.

**Methods:**

A systematic review funded by the Canadian Institutes of Health Research (PROSPERO registration: CRD42013003319) was conducted. Included studies examined the implementation of any KT intervention involving nurses in tertiary care to promote EIDM knowledge, skills, behaviours, and client outcomes or studies that examined contextual factors. Study designs included systematic reviews, quantitative, qualitative, and mixed method studies. The search included electronic databases and manual searching of published and unpublished literature to November 2012; key databases included MEDLINE, Cumulative Index to Nursing and Allied Health Literature (CINAHL), and Excerpta Medica (EMBASE). Two reviewers independently performed study selection, risk of bias assessment, and data extraction. Studies with quantitative data determined to be clinically homogeneous were synthesized using meta-analytic methods. Studies with quantitative data not appropriate for meta-analysis were synthesized narratively by outcome. Studies with qualitative data were synthesized by theme.

**Results:**

Of the 44,648 citations screened, 30 citations met the inclusion criteria (18 quantitative, 10 qualitative, and 2 mixed methods studies). The quality of studies with quantitative data ranged from very low to high, and quality criteria was generally met for studies with qualitative data. No studies evaluated the impact on knowledge and skills; they primarily investigated the effectiveness of multifaceted KT strategies for promoting EIDM behaviours and improving client outcomes. Almost all studies included an educational component. A meta-analysis of two studies determined that a multifaceted intervention (educational meetings and use of a mentor) did not increase engagement in a range of EIDM behaviours [mean difference 2.7, 95 % CI (−1.7 to 7.1), *I*^2^ = 0 %]. Among the remaining studies, no definitive conclusions could be made about the relative effectiveness of the KT interventions due to variation of interventions and outcomes, as well as study limitations. Findings from studies with qualitative data identified the organizational, individual, and interpersonal factors, as well as characteristics of the innovation, that influence the success of implementation.

**Conclusions:**

KT interventions are being implemented and evaluated on nurses’ behaviour and client outcomes. This systematic review may inform the selection of KT interventions and outcomes among nurses in tertiary care and decisions about further research.

**Electronic supplementary material:**

The online version of this article (doi:10.1186/s13012-015-0286-1) contains supplementary material, which is available to authorized users.

## Background

Nurses are expected to use research evidence in practice to improve client and system outcomes. Standards of practice identify evidence-informed decision-making (EIDM) as an important and integral component of effective nursing practice. EIDM involves critically integrating research evidence with information about client preferences, clinical expertise, clinical context, and resources to inform clinical decisions [1–4]. Although awareness about EIDM has increased, it remains inconsistently operationalized for use in practice [5, 6]. In addition to multiple organizational and system factors, nurses continue to lack the necessary knowledge and skills to be able to find, access, and interpret the best available research evidence and subsequently apply, implement, and evaluate its impact on practice [7–10].

Previous systematic reviews have focused on effectiveness of knowledge translation (KT) interventions to promote the use of research evidence among healthcare professionals [5, 6, 9, 11–17]. Active interventions such as alerts, educational outreach, opinion leaders, audit and feedback, and point-of-care computer reminders show small to moderate improvements in EIDM behaviours and client outcomes, with insufficient evidence to support multifaceted interventions over single interventions [5, 6, 9, 11–17]. No analyses were specific to nurses in these reviews. Only one systematic review by Thompson and colleagues [15] considered the effect of KT interventions on nurses’ research use. With a limited number of studies of poor quality, Thompson and colleagues concluded that there was insufficient evidence to support or refute the use of any specific intervention aimed at increasing research use in nursing [15].

Continuing to focus on nurses and expanding outcomes of interest, while addressing practice relevant questions, the knowledge user partners and review team members conducted a systematic review to determine among nurses in tertiary care settings:

1. Are KT interventions effective for promoting EIDM knowledge, skills, and behaviours among nurses?
2. Do KT interventions targeted to nurses affect client outcomes?
3. What contextual factors affect the impact of KT interventions?

## Methods

The search was developed by a review team member (AM) who reviewed existing systematic reviews as a starting point [15, 18]. Databases and other sources were searched on November 2012 (Table 1). Key databases included MEDLINE, Cumulative Index to Nursing and Allied Health Literature (CINAHL), and Excerpta Medica (EMBASE). Additional file 1 (Electronic database search strategy) provides full search details. Table 2 lists study inclusion and exclusion criteria. After de-duplication, two reviewers independently screened citations using predetermined relevance criteria: publication in English, acute care/hospital setting, description of a KT intervention and relevant outcome(s) (nurses’ knowledge or skill for research use, nurses’ research use (behaviour), client outcomes as a result of nurses’ research use, contextual factors for nurses’ research use). Any citation classified as ‘include’ or ‘unsure’ by any reviewer was retrieved. Full-text papers were independently screened by two reviewers for relevance, with a third reviewer to resolve conflicts, using the following criteria: publication in English, study design (systematic review, randomized controlled trial (RCT), cluster RCT, non-randomized cluster controlled trials, controlled before and after studies, interrupted time series, mixed methods, qualitative), description of a KT intervention applied in an acute care setting, report of quantitative or qualitative data, relevant outcome(s) change in nurses’ knowledge or skill for research use, change in nurses’ research use (behaviour), client outcomes as a result of nurses’ research use, and contextual information. See Additional file 2 for full details of the title and abstract and full-text screening criteria.

**Table 1:** Search strategy

Electronic databases (inception until November 22, 2012)
• Cochrane Database of Systematic Reviews
• Database of Abstracts of Reviews of Effects
• Health Technology Assessment Database
• MEDLINE (through PubMed)
• Scopus
• Cumulative Index to Nursing and Allied Health Literature (CINAHL)
• Excerpta Medica (EMBASE)
• Web of Science
• Psychological Abstracts (PsycINFO)
• Education Resources Information Center (ERIC)
• Dissertation Abstracts International
Other sources
• Effective Practice and Organization of Care (EPOC) Register was searched by the EPOC Information Specialist and Trials Search Coordinator (May 22, 2013)
• Hand searches of the references lists of included studies
• Hand search of following key journals for the 12-month period prior to the date the electronic database search was conducted: Implementation Science, BioMed Central Health Services Research, Journal of Health Services Research & Policy, and Nursing Research
• Open Grey (http://www.opengrey.eu/)
• KT Plus (http://plus.mcmaster.ca/kt/),
• Relevant conference proceedings, abstracts, and reports from the following were searched on June 14, 2013:
○ the Research Transfer Network of Alberta (https://web.archive.org/web/20140325202611/http://www.aihealthsolutions.ca/rtna/conference.php)
○ KT Canada (http://ktclearinghouse.ca/ktcanada), Knowledge Utilization Colloquia (http://www.kusp.ualberta.ca/en/KnowledgeUtilizationColloquia.aspx)
○ National Institutes of Health Science of Dissemination and Implementation conferences (http://obssr.od.nih.gov/scientific_areas/translation/dissemination_and_implementation)
○ Joanna Briggs Institute (http://joannabriggs.org/)

**Table 2:** Inclusion and exclusion criteria

Study design
Quantitative designs [84]: Systematic reviews, randomized controlled trials (RCTs), cluster RCTs, non-randomized trials (including controlled before and after studies), cluster non-randomized trials, interrupted time series designs with a clearly defined point in time at which the intervention occurred and at least three data points before and after the intervention, and prospective cohort studies.
Qualitative designs: All qualitative designs (e.g. descriptive, phenomenology, grounded theory). Studies needed to demonstrate that a specific qualitative methodology was followed (e.g. referencing a methodology, describing the analysis).
Mixed methods study designs. Studies needed to adhere to the inclusion criteria for both quantitative and qualitative designs.
Exclusion: Non-systematic reviews, cross-sectional studies, quantitative studies using post-test only, case reports, discussion papers, and editorials.
Setting
Inclusion: Tertiary care.
Exclusion: Studies conducted exclusively in primary care, long-term care, outpatient clinics, or community settings.
Participants
Inclusion: Nurses; registered nurses (RNs), APNs [e.g., clinical nurse specialists (CNSs), nurse practitioners (NPs)], licensed practical nurses (LPNs) or registered practical nurses (RPNs), and student nurses. When the implementation of the intervention involved nurses as part of a group of healthcare professionals and the study met all other inclusion criteria, the citation was included for client outcomes.
Exclusion: Studies in which the intervention was implemented solely among nurses functioning as LPNs, RPNs, or student nurses due to fundamental differences in training, education, and scope of practice. When the implementation of the intervention involved nurses as part of a group of healthcare professionals and the outcomes were group knowledge, skills, and behaviours and effects for nurses could not be isolated.
Interventions
Inclusion: Any KT intervention directed towards target participants and aimed at promoting nurses’ EIDM knowledge, skills, or behaviours, or affecting client outcomes. A list of KT interventions was compiled from similar systematic reviews conducted by the EPOC review group and the UK Health Technology Assessment Programme [6, 8, 71, 84, 101] (e.g. audit and feedback; educational materials, meetings, outreach visits; mass media; reminders).
Exclusion: Implementation of a guideline, which was not developed through a review of the best available evidence and/or accompanied in its implementation by an additional KT intervention.
Outcomes
Inclusion (quantitative): (1) EIDM knowledge, (2) EIDM skills, (3) EIDM behaviour, and (4) any client outcome. Nurses’ EIDM knowledge, skills, and behaviour were conceptualized using the Classification Rubric for Evidence Based Practice (EBP) Assessment Tools in Education framework [17].
Knowledge: Facts and concepts about EIDM. Examples include: the ability to define the components of a clinical question, the ability to identify resources to search for the best available research evidence, or knowledge of critical appraisal concepts.
Skills: The application of knowledge. Examples include the ability to correctly construct a clinical question, appropriately conducted a search of the evidence, or accurately appraise the quality of evidence.
Behaviours: Behaviours reflecting the conduct of EIDM in nursing practice. Examples include identifying and constructing clinical questions, searching for the best available evidence, or critically appraise evidence.
Inclusion (qualitative): Contextual factors influencing the implementation of the KT intervention.

A standardized form was developed, piloted, and refined to extract data for study characteristics and outcomes of interest. Two reviewers independently extracted data, with a third reviewer available to resolve conflicts.

Included studies were independently assessed for quality by two review team members, with a third reviewer available to resolve discrepancies. The Cochrane Collaboration tool for assessing risk of bias was used to assess quantitative primary studies [19]; this included an assessment of sequence generation, allocation concealment, blinding, incomplete outcome data, selective reporting, and other bias. Other bias included industry funding and involvement in some aspect of the study, not adjusting for significant baseline difference in outcome(s), insufficient power, unit of analysis issues, participation rate of <80 %, not the same participants at all data points (not applicable for groups of patients), possibility for co-intervention, and/or contamination.

For qualitative studies, the Joanna Briggs Institute Qualitative Assessment and Review Instrument (QARI) was used [20]. The QARI assesses congruity between the philosophical perspective and research methodology as well as the research methods and objectives, data collection methods, representation and data analysis, and interpretation of the results. In addition to assessing the influence of the researcher on the research (and vice-versa), QARI also assesses if the researcher is located culturally/theoretically, participants and their voices are adequately represented, the research is ethical, and conclusions flow from the analysis/data interpretation. For mixed methods studies, methodological quality was assessed separately for both quantitative and qualitative methods using the aforementioned criteria.

For studies with quantitative data, the results were summarized by outcome taking into account the strength of the quality of the evidence and evidence of effect. The Grading of Recommendations Assessment, Development and Evaluation (GRADE) approach was used to identify the overall quality and strength of the body of evidence to support the confidence in the effect of KT interventions [21, 22]. Using GRADE, the confidence in the findings was judged as ‘high’, ‘moderate’, ‘low’, or ‘very low’ (‘high’, further research was very unlikely to change the confidence in the estimate of effect; ‘moderate’, further research was likely to have an important impact on the confidence in the estimate of effect, possibly changing the estimate; ‘low’, further research was very likely to have an important impact on the confidence in the estimate of effect, thus likely to change the estimate, ‘very low’, when any estimate of effect was very uncertain) [21–23]. In applying GRADE, for continuous outcomes, the minimally important difference (MID) was used to calculate optimal information size (OIS) to determine imprecision [24]. When unable to identify the MID, it was assumed to be half of the reported standard deviation (SD) [25]. When the SD was absent, the MID was determined from data provided by the authors. To account for the size of effect, a relative risk (RR) or odds ratio (OR) of ≥0.5 or ≤2.0 was judged to be a small effect for dichotomous outcomes. For continuous outcomes, the MID determined the effect size. If the effect met the MID, it was judged to be small; if the effect was between the MID to 1.5 times the MID, it was judged to be moderate; and if the effect was 2.0 times the MID, it was judged to be large.

A high level of variation was anticipated between the included studies. Following data extraction, the appropriateness of summarizing studies use meta-analytic methods were made based on comparability of participants, intervention, outcome, and measurement of outcomes. In the presence of clinical heterogeneity, the results were synthesized narratively. For studies judged to be clinically homogeneous and appropriate to combine using meta-analytic techniques, statistical heterogeneity was explored using Cochrane’s *Q* (*a* = 0.10) and *I*^2^ statistic to quantify the magnitude of statistical heterogeneity between studies; *I*^2^ < 50 % representing minimal, *I*^2^ ≥ 50 % representing moderate, and *I*^2^ > 75 % representing substantial statistical heterogeneity across studies. A random effects model, providing a more conservative estimate of effect, was then used to calculate the overall estimation treatment effect.

For studies with quantitative data that did not determine the treatment effect between groups, the treatment effect and corresponding 95 % confidence intervals (CI) were calculated using Review Manager (RevMan) [26] from raw data in the article. Authors were not contacted for missing data; however, in some instances, calculations could be done based on data provided in the studies.

For studies with qualitative data, one reviewer examined the studies and data extraction files to become more familiar with the data, then generated initial codes [27]. The codes were searched for themes, which were then reviewed, defined, and named. A second reviewer verified examples of coding and subsequent themes. The qualitative data were then synthesized narratively by theme.

## Results

**Fig. 1:**
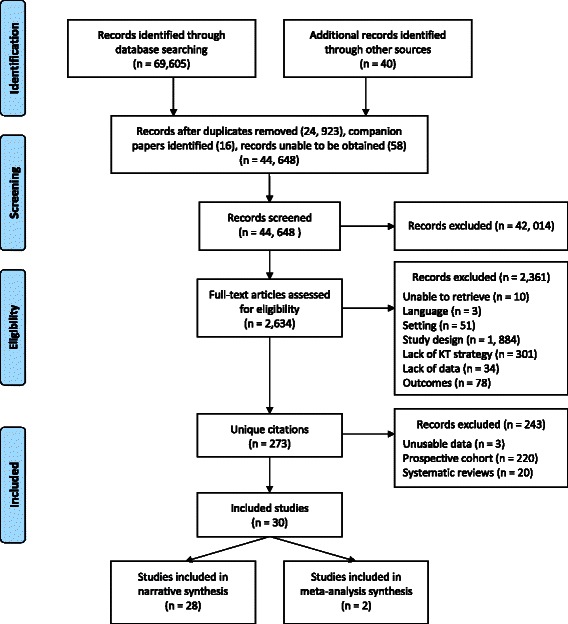
Flow diagram. Yost et al. effectiveness of KT interventions

Following de-duplication, 44,648 unique references were screened (see Fig. 1, flow diagram) and 273 unique references were identified. The list of the excluded studies is available upon request. Twenty systematic reviews were identified and reviewed to identify additional primary studies and to validate the search strategy. Although criteria initially included prospective cohort studies, these (*n* = 220) were subsequently excluded because a large number of studies with stronger methodological designs were found (RCTs, cluster RCTs, non-randomized controlled trials, and cluster controlled trials). The characteristics of the systematic reviews and prospective cohort studies are reported elsewhere [28]*.* During data extraction, three quantitative studies [29–31] that did not provide comparisons between groups were excluded.

Thirty studies met the final inclusion criteria: 18 quantitative studies [32–49], 10 qualitative studies [50–59], and 2 mixed methods studies [60, 61]. The quantitative research designs included five RCTs [34, 39, 43, 45, 47], six cluster RCTs [33, 36, 38, 41, 44, 49], three non-randomized trials [35, 37, 46], and four cluster non-randomized trials [32, 40, 42, 48]. The qualitative research designs included eight descriptive studies [50, 52, 54, 56–59] and two grounded theory studies [51, 53]. The mixed methods studies were non-randomized trials with a qualitative component [60, 61].

Most KT interventions implemented were multifaceted [34, 37, 38, 40–46, 48, 49, 51, 52, 54, 57, 58, 60, 61]. Single interventions included educational meetings [35, 39, 47, 50, 55, 56, 59], educational materials [32, 33], and a clinical decision support system [53]. All included studies incorporated an educational aspect to interventions except one study that implemented computerized decision support [53].

While none of the included quantitative or mixed methods studies considered the outcomes of EIDM knowledge and skills, 12 studies reported on outcomes associated with EIDM behaviours [32–35, 37, 39, 40, 42–44, 48, 60] and 8 studies reported on client outcomes [36, 38, 41, 45–49]. All qualitative and mixed methods studies reported contextual factors influencing the effectiveness of KT intervention implementation. Further details about setting, study design, participants, interventions, and outcomes for included studies are in Additional file 3 (characteristics of included studies).

Most studies reporting quantitative data were at high risk of bias. Criteria judged across studies to be high risk of bias were primarily blinding of participants/personnel and other bias. The most common risk for other bias was lack of power with no power calculation and risk of contamination and/or co-intervention. Overall, the quality of the evidence ranged from very low to high using GRADE, and GRADE tables are available upon request. Most studies reporting qualitative information met the quality criteria. A summary of the quality of the included studies is in Additional file 4 (quality assessment details).

### Intervention effects

The intervention effects of the studies with quantitative data are categorized by the outcome of interest to the review. None of the included studies assessed changes in EIDM knowledge and skills. Outcomes of EIDM behaviours and client outcomes were assessed. In studies evaluating EIDM behaviours, behaviours were either engaging in EIDM behaviours (e.g. searching for the best available evidence, critically appraising research evidence) or the use of research evidence (e.g., an evidence-informed guideline, protocol, pathway) for practice change. The findings of 18 quantitative studies [32–49] and one mixed methods study [60] are presented. The results of the other mixed methods study [61] are not reported on, as the difference in the outcome between groups was not reported by the authors. Table 3 (summary of findings) presents a summary of findings by KT intervention and outcome and Additional file 5 (outcomes tables) provides further details about the intervention effects on EIDM behaviours and client outcomes, including the effect estimates and confidence intervals for all studies.

**Table 3:** Summary of findings

Outcome	Impact	Number of studies	Confidence in the findings (GRADE)
Single educational intervention versus control			
Engaging in EIDM behaviours	Evidence of effect is mixed	1 non-randomized trial [35]	Very low
Use of research evidence for practice change	Evidence of effect is mixed	1 cluster RCT [33]	Unable to be assessed; serious study limitations
Client outcomes	Evidence of no effect	1 RCT [47]	Moderate
Single educational intervention versus single educational intervention			
Engaging in EIDM behaviours	Evidence of an effect for all outcomes	1 RCT [39], 1 cluster non-randomized trial [32]	Very low to moderate
Multifaceted intervention versus control			
Engaging in EIDM behaviours	Evidence of no effect	2 non-randomized trials [37, 60], 1 cluster non-randomized trial [42]	Very low to low
Use of research evidence for practice change	Evidence of effect for most outcomes	2 RCTs [34, 43]; 1 cluster non-randomized trial [48]	Very low to moderate
Client outcomes	Evidence of effect is mixed	1 RCT [45], 1 cluster RCT [41], 1 non-randomized trial [46], 1 cluster non-randomized trial [48]	Low to moderate
Multifaceted intervention versus single educational intervention			
Client outcomes	Evidence of effect is mixed	1 RCT [36]; 1 cluster RCT [38]	Moderate to high; no serious risk of bias
Multifaceted intervention versus multifaceted intervention			
Engaging in EIDM behaviours	Evidence of effect	1 cluster non-randomized trial [42]	Low
Use of research evidence for practice change	Evidence of effect is mixed	1 RCT [43]; 1 cluster RCT [44], 1 cluster non-randomized trial [40]	Very low to moderate
Client outcomes	Evidence of effect is mixed	1 cluster RCT [49]	Moderate to high

#### Engaging in EIDM behaviours

**Fig. 2:**
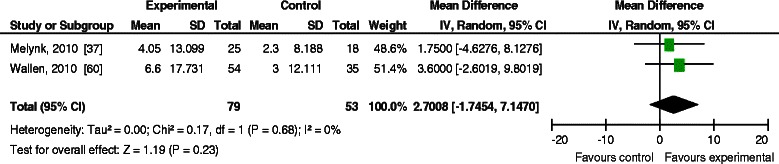
Meta-analysis: effect of educational meetings and use of a mentor for engaging in a range of evidence-based practice behaviours. Yost et al. effectiveness of KT interventions

Of four studies implementing KT interventions to promote engagement in EIDM behaviours [35, 37, 42, 60], three did not have an effect, with very low to low confidence in the findings. Two studies evaluated the effectiveness of educational meetings followed by the use of a mentor to promote a range of EIDM behaviours [37, 60]. The meta-analysis (Fig. 2) found that multifaceted KT interventions (educational meetings and use of a mentor) did not increase change in self-reported engagement in a range EIDM behaviours at 6 months compared to no intervention [WMD (weighted mean difference) = 2.7, 95 % CI (−1.7, 7.1) *P* = 0.23, *I*^2=^ 0 %] as measured by the EBP implementation scale [37, 60].

In the two other studies, there was evidence of no effect for multifaceted interventions on use of research results [35] and incorporation of research evidence into practice decisions [42]. Tsai [35], however, demonstrated an effect comparing the impact of 8 weeks (~40 h) of educational meetings on research utilization to usual practice. The intervention had a small effect on nurses’ self-reported participation in research (measured on a scale from 0 to 33, higher score indicative of greater participation) immediately post-intervention [MD (mean difference) = 4.00, 95 % CI (0.55, 7.45) *P* < 0.001; Note: MD and 95 % CI calculated using study data, hereafter referred to as ‘calculated by review team’] and at 6 months post-intervention [MD = 4.50, 95 % CI (1.05, 7.95) *P =* 0.003; calculated by review team]. The clinical significance of the small effect of this time- and cost-intensive intervention is unclear and due to serious study limitations, confidence in the findings of this study is very low.

#### Use of research evidence for practice change

Eight studies evaluated KT interventions to promote the use of research evidence (in the form of an evidence-informed guideline, protocol, or pathway) for a practice change [32–34, 39, 40, 43, 44, 48]. The direction of effects was similar across studies with effect sizes for increase in use of research evidence ranging from small to large [32–34, 39, 40, 43, 44, 48]; all but one [43] demonstrated an effect. Differences in types of KT interventions and measurements of outcomes across studies provide possible explanations for differences in effect size between the studies. However, other components (e.g. study quality, power analysis, use of theory, similarities in baseline characteristics, intervention length, and follow-up) were similar across studies. Most studies ranged from very low to moderate in confidence of the findings.

Single-component educational interventions compared to control or no intervention resulted in the largest effects on nurses’ use of research evidence. In the study by Day and colleagues [39], nurses who received a 2-hr educational programme which included didactic, interactive, and practical teaching demonstrations on endotracheal suctioning increased their mean performance score by 9.88 points more [95 % CI (3.42, 16.34)] at 4 days and 4 weeks after the intervention compared to nurses in the control group [nurses who received the same type of intervention, but on an unrelated topic (humidification for mechanical ventilation)]. This represents a large and potentially clinically important effect (assuming a MID of 3 points) in which there is moderate confidence.

The study with the next largest effect also implemented a single component educational intervention [33]. Kirshbaum [33] mailed to breast care nurses a booklet of evidence-informed guidelines for recommending exercise to their clients. Nurses in the control group received the booklet after the intervention period. Nurses who received the intervention were more likely than those in the control group to recommend exercise for nausea [OR 2.54, 95 % CI (2.53, 13.20)], loss of appetite [OR 3.67 9, 95 % CI 1.82, 3.76], and fatigue [OR 2.44, 95 % CI (1.12, 5.99)] consistent with the guideline; with no difference in making recommendations about weight gain, insomnia, loss of libido, panic attacks, altered body image, and headaches. While confidence in the findings could not be assessed, this study had serious study limitations due to potential detection bias.

Studies using self-report [33, 40, 44] or observational measures [39, 48] demonstrated an effect compared to studies using chart audits, which demonstrated evidence of no effect [32, 43]. The implementation of educational meetings and materials with group feedback, individual feedback, or no feedback demonstrated evidence of no effect on nurses’ correct completion of Braden scoring upon admission (*P* = 0.38) and implementation of a care plan in accordance with the practice guideline for clients at risk or with stage 1 pressure ulcers (*P* = 0.85) [43]. There is moderate confidence in the findings of this study. In another study, the delivery of an open-book examination and individual competency assessment increased the chance that nurses were compliant with detoxification guidelines by 28 % relative to nurses who received only educational meetings [RR 1.28, 95 % CI (1.1, 1.48) *P* 0.000; calculated by the team] [32]. This is a small effect size, and confidence in the findings of this study is very low. The degree of completeness and accuracy of chart audit data might explain the lack of treatment effects [62]. Neither of these studies reported development and testing of the audit tool or extraction process [63]. Missing or incomplete documentation was only acknowledged by one of the studies [43].

Studies reporting either validity [33, 44] or reliability [48], or both [39, 40], of the measurement tools demonstrated greater effects compared to studies that did not report reliability or validity [34, 43]. In one study without reported psychometric properties [43], there was evidence of no effect of a multifaceted KT intervention on practice improvements. Girouard [34] also did not report reliability and validity and demonstrated no change in reporting preoperative teaching by nurses and a small change in preoperative teaching as reported by clients. Nurses who received educational meetings, educational materials, and reminders taught 2.8 items more than those who did not receive the KT intervention, as measured by client report [MD 2.8 items, 95 % CI (1.57, 4.03) *P* < 0.004; calculated by review team]; there is low confidence in this finding. Studies with reported reliability and validity, however, demonstrated small to large effects. Hyndman and colleagues [40] used educational materials (clinical practice guideline, self-study package, and a 15-min video), educational outreach visits, and mass media compared to only educational meetings and mass media and found a small to moderate effect on the mean adherence score to the practice guideline for tobacco use and dependence [MD 6.5 at 10 weeks, 95 % CI (3.35, 9.65) *P* < 0.001; calculated by review team]. However, there is very low confidence in this finding. Day and colleagues [39] also used reliable and valid tools and had an effect as reported above. These examples indicate that use of valid and reliable measurement tools may have contributed to identification of the effect of the KT intervention on nurses’ use of research evidence.

#### Client outcomes

The use of research evidence (in the form of guideline, protocol, or pathway) along with an additional KT intervention assessed client outcomes in eight studies [36, 38, 41, 45–49]. The effects were mixed with studies finding evidence of no effect [41, 45, 47] or an effect [36, 38, 46, 48, 49]. The confidence in the findings of most studies was moderate to high [36, 41, 45, 47, 49]; the confidence for other studies were very low [46], low [46, 48], or unable to be rated [38]. Differences in the interventions, follow-up, confidence in the findings, use of theory, baseline characteristics, and power analysis do not appear to explain differences in effect sizes. Possible explanations for differences include study design and outcome.

Among the cluster RCTs [38, 41, 48, 49] and cluster non-randomized trials [46], contamination, as a function of allocation, may have influenced the intervention effects. Larger effects (small to large) were demonstrated in studies where allocation was done by hospital [36, 38, 48] versus evidence of no effect and small effects in studies where the KT intervention was allocated by unit within a hospital with multiple participating units thus introducing the possibility of contamination [41, 46, 49]. Contamination was unlikely in the study by Wesorick and colleagues [46] due to the use of a historic control in which the outcome data were collected from chart audits during the same months 2 years prior. Other factors, in this study with low confidence in the findings, may have contributed to the small effect size in percent patient days being hypoglycemic [OR 0.48, 95 % CI (0.27, 0.85) *P* = 0.01]. Dykes and colleagues [49] matched medical units in four hospitals in Boston with fall rates higher than the mean for the institution the year before, to units with similar fall rates and patient days, then randomized them. Clients cared for by healthcare professionals who received a fall prevention toolkit, use of computerized decision support, and mass media (posters above patient beds) experienced a small reduction in falls compared to clients receiving care from healthcare professionals who received usual education related to fall prevention [1.03 fewer falls per 1000 clients days, 95 % CI (−2.01, −0.57) *P =* 0.04]. Seers and colleagues [41] conducted a study within a single specialist orthopaedic hospital. Two surgical wards were randomized to receive the KT intervention and two surgical wards to control. Audit and feedback, approximately 10 h of educational meetings, and the use of an algorithm on the intervention units resulted in no differences in client report of pain intensity compared to the control units (no intervention). There was the possibility of contamination due to the allocation by unit within the hospital and a possibility that the ward staff allocated to the control unit knew results of the baseline audit and contributed to the development of the algorithm.

Similarly, studies reporting either reliability [36], validity [48], or both reliability and validity [38] of the measurement of client outcomes demonstrated greater effects compared to studies that did not report reliability or validity [41, 46, 47] or simply reported that instruments were validated [45, 49]. Studies without reported reliability and validity demonstrated evidence of no effect on pain [41] and length of stay [45, 47] and small effects on diabetic control [46] or falls [49].

Middleton and colleagues [36, 64, 65] implemented of a multifaceted intervention to implement clinical treatment protocols based on a clinical practice guideline in acute stroke units which demonstrated small effects for a number of client outcomes, of which the reduction in death and dependency was clinically important. Compared to clients receiving care from healthcare professionals who received access to the guideline only and clients cared for by healthcare professionals (including nurses) receiving the intervention, there was evidence of a reduction in mean temperature (°C) [MD 0.09, 95 % CI (0.04, 0.15), *P* = 0.001], the risk of temperature greater than 37.5 °C [RR, 0.64; 95 % CI (0.51, 0.81), *P* ≤ 0.0001; RR and 95 % CI calculated by review team], and mean glucose [MD 0.54, 95 % CI (0.08, 1.01), *P* = 0.02] during the first 72 h in an acute stroke unit, as well as reduction in the risk of death or dependence [RR 0.72, 95 % CI 0.65, 0.84, *P* < 0.002; RR and 95 % CI calculated by review team] and an increase in physical health [MD 3.4, 95 % CI (1.2, 5.5), *P* = 0.002]. Despite moderate to high confidence in these findings, the presence of mixed findings may limit the transferability of the findings; there was evidence of no effect for increasing mental health and functional dependence, as well as decreasing the risk of pneumonia upon discharge and length of stay.

The effect of one educational meeting which addressed guidelines for assessing and managing pain in older adults (6 h of instruction and 2 h of practical demonstration) was evaluated by Manias and colleagues [48]. Clients of nurses in the intervention group experienced reductions in pain intensity compared to clients cared for by healthcare professionals receiving usual staff development activities. Pain intensity on movement measured using a visual analogue scale of 0 to 10 (validated tool; adequate reliability) was reduced by 2.15 units [95 % CI (−3.19, −1.11), *P* < 0.0001] immediately post-intervention and by 2.49 units 3 months post-intervention [95 % CI (−3.54, −1.44), *P* < 0.0001]. Pain intensity at rest was reduced by 1.65 units [95 % CI (−2.79, −0.52), *P* = 0.004] immediately-post intervention. The reduction in pain intensity 3 months post-intervention could be considered clinically important; however, this study was judged to have low confidence in the findings.

Titler [38] reported validity and reliability for the medical record abstract form used to collect pain intensity outcome data. Clients hospitalized with a hip fracture and cared for by healthcare professionals who received a multifaceted KT intervention experienced a 2.5-unit greater reduction in pain intensity during the first 24 h and a 1.5-unit greater reduction in pain intensity over 72 h compared to clients who were cared for by healthcare professionals who only received copies of the guideline being implemented. The multifaceted KT intervention involved educational materials, meetings, and outreach visits; mass media; opinion leaders; change champions; and audit and feedback. While the reductions in pain intensity during the first 24 h met the MID difference of 2.0 points [66], the substantial number of components of the intervention may limit transferability.

Three studies considered the effect of multifaceted KT interventions to promote the use of evidence-informed guidelines and pathways on length of stay [36, 45, 47]; confidence in their findings was moderate to high. Educational meetings followed by the use of an integrated care pathway [47] or standard assessment form [45] compared to usual care did not result in an effect on length of stay. Educational meetings, educational outreach, and reminders compared to educational materials alone also demonstrated evidence of no effect on length of stay [36]. Multiple factors can influence length of stay; however, these factors were not accounted for in these KT intervention studies. This limitation makes it difficult to draw any conclusions about the effectiveness of KT interventions on length of stay.

Three additional studies considered the effect of multifaceted KT interventions on pain intensity with multiple possibilities for the variable results [38, 41, 48]. For example, clients in the study by Seers and colleagues [41] were asked to report their pain since surgery 3 to 5 days after surgery, while the outcomes for Mania and colleagues [48] and Titler and colleagues [38] were abstracted from medical record audits where outcomes were recorded in ‘real-time’ during nurses’ assessments. The larger effect demonstrated by Mania and colleagues [48] may be due to unreported differences at baseline between the intervention and control groups.

### Contextual factors influencing degree of success of KT interventions

Thirteen studies explored contextual factors related to success of KT interventions. Studies included 11 reports of 10 qualitative studies [50–59, 67] (all descriptive design, except for 2 grounded theory studies [51, 53]), 3 reports of two mixed methods studies [60, 61, 68], and 3 quantitative studies [37, 41, 44]. All studies included at least one educational component, and several were multifaceted. Most authors concluded that implementation of KT interventions was a complex undertaking, but factors related to success of KT strategies were identified at the level of the organization, the individual, and the planned innovation. Overall, the studies were considered to be of high quality. The findings are summarized below by themes.

#### Organizational factors

In interviews with nurses, managers, clinical nurse specialists (CNSs), and champions regarding barriers and facilitators, organizational factors were strongly related to the success of implementation of KT interventions. Organizational leadership emerged as a major theme [61, 68]. Leaders who gave support and encouragement, who prioritized the implementation project as important [56] and who had a positive attitude to the project or implementation were more likely to see greater success with implementation projects. Leadership strategies associated with sustained guideline use included facilitating staff to use guidelines, creating a positive environment of best practices, and influencing organizational structures and processes. Behaviours associated with these strategies included providing support, role modelling commitment, and reinforcing organizational policies and goals for evidence-based care [51, 67]. Other effective leadership contributions were supporting staff through adjusting workloads, allowing time to consider the evidence, and providing resources to free up staff time to support engaging in EIDM [50, 56, 61, 68]. These mechanisms associated with finding time were critical even with very intensive and extensive interventions [56].

Within an organization, Melnyk and colleagues [37] found that a multifaceted educational intervention did not have an effect on EIDM behaviour. Findings of focus groups conducted with a convenience sample suggested that the CNSs were most knowledgeable about evidence-based practice, followed by nurse managers; but success was dependent on grassroots staff and administrative involvement. Leadership support and the allocation of resources were identified as key facilitators for continuing to engage staff at all levels.

Another intervention was aimed at promoting culture change to facilitate greater interaction between research and nursing practice in a mental healthcare setting. Participants believed their involvement in the Nursing Clinical Development Unit Program changed nursing practices and influenced a culture shift on their clinical units, which was exemplified by staff nurses reflecting on practice and questioning the rationale for nursing actions and staff increased reading and critiquing of research evidence [55].

#### Individual and interpersonal factors

Individual staff nurse interest in research and the degree to which each was reading research regularly [56] facilitated research use, while reluctance to change and lack of time was a barrier [44, 61, 68]. However, when these attributes were measured, as opposed to being narratively reported, improved adherence to a protocol without alterations in propensity to change or attitudes to nursing research was found [44].

Nurses’ behaviours were not shaped solely by their personal attributes but shaped by interpersonal factors within their organizations [61, 68]. Individuals who perceived support by other managers, other nurses, and physician colleagues were more likely to utilize research after an educational programme [61, 68].

However, individual nurses’ perceptions of potential criticism by staff colleagues was a barrier. In the study by Royle and colleagues [57], staff perceived that organizational support was sufficient, where convinced clients would benefit from the chosen implementation of evidence, but felt that other staff members would criticize them for the time it would take to carry out the intervention with one client. This perception was consistent with a study by Seers who found that some nurses perceived that evidence-based practice was not always seen as ‘real’ work and not a legitimate activity when nurses were busy [41].

#### Characteristics of the innovation

Educational interventions for evidence-based practice were associated with lower research utilization if the focus of the education or discussion intervention was on the conduct of research versus the utilization of evidence in practice. In contrast, the use of planned implementation strategies [50], comprehensive training, and the effectiveness of staff education facilitated research uptake [61, 68].

Two studies assessed facilitation as a component of the Promoting Action on Research Implementation in Health Services (PARIHS) Framework. That framework has three key interacting elements that influence successful implementation of evidence-based practices: *evidence*, *context*, and *facilitation* [58, 59]. Using a mixed methods design, Wallin and colleagues [59] introduced a guideline in four clinical units, where two received additional facilitation strategies. Facilitation in the intervention units was no more effective than a focus on improved organizational culture where the nurse manager was actively involved in the change process. The authors concluded that implementation process is a social phenomenon that benefited from interaction. In one control group where no significant change activities were carried out, the guideline was regarded as trivial and not used. The other three units found the guideline to contain important knowledge. Thus, the successful aspect of implementation seemed to be the incorporation of a change team to manage the implementation of the guideline, not the external facilitation [59]. Ellis and colleagues (2005) [58] used the PARIHS framework to explore the relative and combined importance of context and facilitation in their successful implementation of a new evidence-based clinical practice protocol in six rural hospitals. A 1-day educational workshop was held and follow-up support was given. All hospitals except one were successful in implementing a new protocol. The researchers found that the context of each hospital was different and that no hospital rated high in all domains of context (culture, leadership, evaluation). The rate of adoption varied from 2 weeks to months. Participants reported being better informed about evidence-based practice in general and were positive about their ability to improve practice and search more efficiently for best practice information. In this study, good facilitation appears to be more influential than context in overcoming barriers to uptake of evidence-based practice.

## Discussion

This review identified that KT interventions are being implemented and evaluated to enhance EIDM among nurses in tertiary care and, in turn, to promote client outcomes. Due to the variety of methodologies, clinical areas, interventions, outcomes, and outcome measurement among studies in this systematic review, recommendations cannot be drawn about the relative effectiveness of single or multifaceted KT interventions or components of these interventions. This is similar to the conclusion of previous systematic reviews [5, 6, 11–17, 69] and overviews of reviews [5, 70]. Although they have at times discerned the degree of effectiveness of specific KT interventions, implemented as a single intervention, component of a multifaceted intervention, or multifaceted KT intervention, these reviews generally concluded that specific KT interventions resulted in either evidence of no effect or small to moderate effect sizes, with broad implementation not recommended.

Within this review, KT interventions for promoting EIDM among nurses are primarily limited to educational interventions alone or educational interventions as components of multifaceted intervention, similar to the existing literature [6, 15, 18]. All studies of multifaceted KT interventions included at least one educational component. With the exception of two studies, single interventions implemented were also educational interventions. Evidence from previous systematic reviews reports that educational interventions resulted in small to moderate improvements in engaging in EIDM behaviours and use of research evidence for practice change [6, 71] and that interactive education is more effective than didactic education [71–73]. Conversely, some researchers have identified that educational interventions are not effective for incorporating research evidence for practice change [15, 18] and client outcomes [74]. The findings of this systematic review were mixed. The use of single-component educational interventions (educational materials or educational meetings) compared to a control or no intervention explained the largest effects on nurses’ use of research evidence and resulted in small effects on nurses’ engagement in EIDM behaviours; however, this did not explain differences in effects of KT interventions between the studies on client outcomes. In addition, multifaceted interventions where educational materials or meetings were one of the components demonstrated a range of no change to significant improvements in these outcomes. Despite finding that educational interventions were the most frequent type of KT intervention implemented, differences such as the intensity, length, and delivery method prevent drawing conclusions that certain types of educational interventions are more effective than others.

This systematic review also considered qualitative and mixed methods studies, which broadens the included research designs to study implementation research [75–78]. Within the qualitative and mixed methods studies, this systematic review identified consistent characteristics that were considered facilitators: positive interpersonal relationships, supportive environment, shared governance, and leadership; ability to engage staff nurses at different junctions and to overcome negative reactions to practice changes; and allocation of resources and administrative support. Leadership was identified as integral to supporting the use of evidence in practice [44, 50–52, 54, 56, 59–61, 67], which is consistent with other literature [79–81]. Within this systematic review, some studies targeted the intervention to leaders within an organization to garner leaders’ support of staff [38, 60], selecting staff to participate in the intervention [53], or skilling up the leaders on a particular leadership style [55]. While it is not clear what forms of leadership support implementation of evidence into practice, involving leadership in the implementation appears to contribute to effectiveness.

In addition to the identification of facilitators, context was found to be an important factor, different in each acute care setting and equally important to consider as the strength of the research evidence. Context is widely cited throughout the literature as being important to the implementation of evidence into practice [18, 81, 82]. Authors have suggested that a more positive context predicts research use [82] and that contextual factors likely contribute to environments that are conducive for implementing evidence into practice [83]. Although contextual variability between the included studies and previous systematic reviews makes it difficult to make specific generalizations, evidence suggests that a positive context contributes to nurses’ use of research in practice.

### Recommendations for nursing practice

The evidence in the this review can be considered when making decisions about selecting, adapting, implementing, and evaluating KT interventions for promoting EIDM behaviours and client outcomes. Relevant research evidence for practice exists, in the form of evidence-based guidelines, protocols, pathways, (e.g. endotracheal suctioning [39], preoperative teaching [34], prevention of pressure ulcers [43], and falls [49]). Continuing to implement and evaluate the implementation of research evidence in practice is recommended. In assisting nurses to implement and evaluate the use of evidence in practice, there is value in the use of educational KT strategies. Consistent increases in the nurses’ engagement in EIDM behaviours and use of research were demonstrated with use of educational materials or educational. In addition, based on qualitative findings, involving leadership in the implementation of KT strategies also appears to contribute to improving EIDM among nurses.

### Directions for future research

From this review, several recommendations for future research have been identified. As a high number of prospective cohort studies (*n* = 221) were identified, it highlights the need for more robust study designs to evaluate KT interventions. While the use of a comparison group may not be feasible in many tertiary care settings, interrupted time series is a more rigorous and may be a more realistic methodology. Researchers and nurses involved in the implementation of KT interventions as part of continuous quality improvement efforts can identify a clearly defined point in time at which the KT intervention is implemented and ensure outcome measurement with at least three data collection points before and after the intervention [84]. The use of mixed methods designs should continue to evaluate the implementation of KT interventions to capture facilitators, barriers, and contextual factors that contribute to the success or failure of interventions [76–78].

Improvement in the reporting of implementation is also needed; even the most detailed studies in this review failed to report important aspects of studies, such as detailed descriptions of KT interventions and adherence and fidelity to the interventions. This lack of reporting has been identified in similar systematic reviews [7, 11, 15, 18, 71, 85, 86], and multiple authors have found that interventions are only described in detail 5 to 30 % of the time [18, 87, 88]. While recommendations for reporting the implementation of interventions in RCTs, as well as non-randomized and observational studies exist [88–92], recently, recommendations for the reporting of KT interventions have been developed by the Workgroup for Intervention Development and Evaluation Research (WIDER) [87] and should be followed.

The process of integrating quantitative, qualitative, and mixed methods designs into reviews of complex interventions is largely underdeveloped [78]. To be included in this review, mixed methods studies had to meet both qualitative and quantitative inclusion criteria. This excluded multiple studies that were prospective cohort studies [93–96]. While other recommended practices exist for incorporating qualitative, quantitative, and mixed methods research into systematic reviews of complex interventions [78, 97, 98], little guidance is available on how to integrate inclusion/exclusion criteria of mixed methods research within a review including quantitative and qualitative research. Further exploration is recommended on the best approaches to integrate different study designs so that the research philosophy, analytical technique, strategies, and interpretations can all play an important part in the synthesis of the findings [76].

Finally, this review used the EPOC Group’s (http://epoc.cochrane.org) classification system for organizing and describing interventions. While useful for drawing comparisons, these labels did not capture the types of interventions implemented within all of the studies included in this review. We were unsure of how to categorize strategies related to empowerment and celebrating successes [60]. While EPOC classifications should continue to be used, they may need to be expanded to acknowledge the complexity of interventions [78, 99] and to assist in comparisons across studies.

### Limitations

This systematic review has several limitations. Language was limited to English. An update of this review is indicated; six included studies were published 2 years prior to the end of the search strategy (November 2012), and thus, it is likely that eligible studies have been published since the end of the search. Also, the knowledge user partners and review team members determined that the implementation of a guideline, protocol, or pathway needed to meet two criteria, that the authors needed to indicate that the guideline, protocol, or pathway being implemented (1) had been informed/developed through a review of the best available research evidence and (2) had been accompanied in its implementation by an additional KT intervention. Therefore, this systematic review does not reflect the body of literature on guidelines, protocols, or protocols as a sole KT intervention for nurses in tertiary care. For mixed methods studies, we applied the individual (qualitative and quantitative) inclusion criteria which limited the number of mixed methods studies. Four mixed methods, studies were excluded because the quantitative aspect was a prospective cohort design [94–96, 100].

The confidence in the findings, determined by applying the GRADE criteria, resulted in most studies with quantitative data being of very low to low confidence in their findings. Furthermore, due to differences between the studies, only two studies were able to be synthesized using a meta-analysis. Lastly, given the inclusion and exclusion criteria, the generalization of the findings is limited to nurses working within tertiary care and is not representative of other settings (e.g. long-term care) or populations (e.g. student nurses).

## Conclusions

This systematic review addressed a gap in the literature and was also relevant, timely, and useful for the partners involved. Interventions are being implemented and evaluated to enhance the EIDM behaviours of nurses in tertiary care and to assess the effects on client outcomes. Implementing single-component educational interventions and multifaceted interventions with an educational component appear to have value for promoting nurses’ EIDM behaviours, while multifaceted interventions with an educational component were shown to contribute to improvements in client outcomes. Based on the review of contextual factors, leaders within an organization should be involved in the implementation of KT interventions; their involvement appears to positively influence EIDM among nurses. Above all, decision makers can refer to the synthesis and included studies in the review to assist in selecting KT interventions to be applied to their local context to promote evidence-informed nursing practice for the delivery of quality client care.

## Additional files

Additional file 1: **Electronic database search strategy.** This file provides full details of the electronic database search strategy.
Additional file 2: **Screening criteria.** This file provides full details of the title and abstract and full-text screening criteria.
Additional file 3: **Characteristics of included studies.** This file provides the details of the characteristics of the included studies identified during data extraction.
Additional file 4: **Quality assessment details.** This file provides the risk of bias assessment for the included studies. This includes the review author’s judgements about each quality criteria for each of the final included studies.
Additional file 5: **Outcomes tables.** This file provides the citation, measurement period, brief description of the intervention and control groups, outcome measurements at baseline and follow-up, and overall effect estimates and confidence intervals for included studies with quantitative data.

## Abbreviations

APN: advanced practice nurse
CI: confidence interval
CNS: clinical nurse specialist
EBP: evidence-based practice
EIDM: evidence-informed decision-making
EPOC: Effective Practice and Organization of Care
GRADE: Grades of Recommendation, Assessment, Development and Evaluation
KT: knowledge translation
LPN: licensed practical nurse
MD: mean difference
MID: minimally important difference
NP: nurse practitioner
OIS: optimal information size
OR: odds ratio
PARIHS: Promoting Action on Research Implementation in Health Services
QARI: Qualitative Assessment and Review Instrument
RCT: randomized controlled trial
RN: registered nurse
RPN: registered practical nurse
RR: relative risk
SD: standard deviation
WMD: weighted mean difference
