# Supplementary material for: The effectiveness of knowledge translation interventions for promoting evidence-informed decision-making among nurses in tertiary care: a systematic review and meta-analysis
Source: Implement Sci. 2015 Jul 14;10:98. doi: 10.1186/s13012-015-0286-1 (PMC4499897; doi:10.1186/s13012-015-0286-1)
Supplement: Additional file 1: — Electronic database search strategy. This file provides full details of the electronic database search strategy. [file 13012_2015_286_MOESM1_ESM.pdf]

Additional file 1: Electronic database search strategy

Cochrane Library (Inception – November 20, 2012)

1. Any MeSH descriptor with qualifier(s): [Nursing - NU]
2. MeSH descriptor: [Education, Nursing, Continuing] explode all trees
3. nurse or nursing
4. #1 or #2 or #3
5. chang* near/2 agent*
6. coordinat* near/2 chang*
7. critical* near/1 apprais*
8. "diffusion of innovation"
9. MeSH descriptor: [Decision Making] explode all trees

Centre for Reviews and Dissemination (Inception to November 22, 2012)

1. (nurse or nursing or nurses) AND (english:lp)
2. (chang* near2 agent*) OR (agent* near2 chang*)
3. (coordinat* near2 chang*) OR (chang* near2 coordinat*)
4. (critical* near1 apprais*) OR (apprais* near1 critical*)
5. ("diffusion of innovation") OR (decision making)
6. (((Knowledge or evidence or research or guideline*) near2 (utiliz* or utilis* or uptake or transfer or translat* or transmit* or transmission or effectiveness or populari* or exchange or synthes* or transform* or linkage* or disseminat* or implementation or exchange))) OR (((utiliz* or utilis* or uptake or transfer or translat* or transmit* or transmission or effectiveness or populari* or exchange or synthes* or transform* or linkage* or disseminat* or implementation or exchange) near2 (Knowledge or evidence or research or guideline*)))
7. ((Evidence near2 informed)) OR ((Quality near2 (assurance or improvement or service)))
8. (Best practice*) OR ((facilit* near2 chang*))
9. (informed near2 evidence) OR ((chang* near2 facilit*)) OR (((assurance or improvement or service) near2 quality))
10. #2 OR #3 OR #4 OR #5 OR #6 OR #7 OR #8 OR #9 7287 Delete
11. #1 AND #10

CINAHL (Inception – November 15, 2012)

S1. (MH "Nurses+")
S2. (MH "Specialties, Nursing+")
S3. (MH "Nursing Practice+")
S4. (MH "Nursing as a Profession")OR (MH" Practical Nursing")
S5. (MH "Education,Nursing, Continuing") OR (MH "Education, Nursing, Practical") OR(MH "Education, Nursing, Research- Based")
S6. “nurse educator"
S7. MJ NU
S8. S1 OR S2 OR S3 OR S4 OR S5 OR S6 OR S7
S9. “diffusion of innovation"
S10. chang* n2 agent*
S11. facilit* n2 chang*
S12. coordinat* n2 chang*
S13. critical* n1 apprais*
S14. (MH "Decision Making+")
S15. (Knowledge or evidence or research or guideline*) n2 (utiliz* or utilis* or uptake or transfer or translat* or transmit* or transmission or effectiveness or populari* or exchange or synthes* or transform* or linkage* or disseminat* or implementation or exchange)
S16. Evidence n2 informed
S17. Quality n2 (assurance or improvement or service)
S18. Best practice*
S19. S9 OR S10 OR S11 OR S12 OR S13 OR S14 OR S15 OR S16 OR S17 OR S18
S20. S8 AND S19

EMBASE (1980 to 2012 Week 46)

1. exp nursing
2. exp nurse
3. nursing education/ or research based nursing education
4. (nurse educator$ or nursing educator$).mp.
5. or/1-4
6. (chang$ adj2 agent$).mp.
7. (coordinat$ adj2 chang$).mp.
8. (critical$ adj1 apprais$).mp.
9. diffusion of innovation.mp.
10. exp decision making/
11. ((Knowledge or evidence or research or guideline:) adj2 (utiliz$ or utilis$ or uptake or transfer or translat$ or transmit$ or transmission or effectiveness or populari$ or exchange or synthes$ or transform$ or linkage: or disseminat$ or implementation or exchange)).mp.
12. (Evidence adj2 informed).mp.
13. (Quality adj2 (assurance or improvement or service)).mp.
14. Best practice$.mp.
15. (facilit$ adj2 chang?).mp.
16. or/6-15
17. 5 and 16
18. 5 and 16
19. limit 18 to (human and english language)

ERIC (Inception – November 20, 2012)

1. all(nursing) or all(nurse?)
2. all(chang* N/2 agent*)
3. all(coordinat* N/2 chang*)
4. all(critical* N/2 apprais*)
5. all("diffusion of innovation") or all("diffusion and innovation")
6. all("decision making") or all("decision-making") or all("decision maker") or all("decision makers")
7. (all(Knowledge N/2 utiliz*)) or (all(Knowledge N/2 utilis*)) or (all(Knowledge N/2 uptake)) or (all(Knowledge N/2 transfer)) or (all(Knowledge N/2 translat*)) or (all(Knowledge N/2 transmit*)) or (all(Knowledge N/2 transmission)) or (all(Knowledge N/2 effectiveness)) or (all(Knowledge N/2 populari*)) or (all(Knowledge N/2 exchange)) or (all(Knowledge N/2 synthes*)) or (all(Knowledge N/2 transform*)) or (all(Knowledge N/2 linkage*)) or (all(Knowledge N/2 disseminat*)) or (all(Knowledge N/2 implementation)) or (all(Knowledge N/2 exchange))
8. (all(evidence N/2 utiliz*)) or (all(evidence N/2 utilis*)) or (all(evidence N/2 uptake)) or (all(evidence N/2 transfer)) or (all(evidence N/2 translat*)) or (all(evidence N/2 transmit*)) or (all(evidence N/2 transmission)) or (all(evidence N/2 effectiveness)) or (all(evidence N/2 populari*)) or (all(evidence N/2 exchange)) or (all(evidence N/2 synthes*)) or (all(evidence N/2 transform*)) or (all(evidence N/2 linkage*)) or (all(evidence N/2 disseminat*)) or (all(evidence N/2 implementation)) or (all(evidence N/2 exchange))
9. (all(research N/2 utiliz*)) or (all(research N/2 utilis*)) or (all(research N/2 uptake)) or (all(research N/2 transfer)) or (all(research N/2 translat*)) or (all(research N/2 transmit*)) or (all(research N/2 transmission)) or (all(research N/2 effectiveness)) or (all(research N/2 populari*)) or (all(research N/2 exchange)) or (all(research N/2 synthes*)) or (all(research N/2 transform*)) or (all(research N/2 linkage*)) or (all(research N/2 disseminat*)) or (all(research N/2 implementation)) or (all(research N/2 exchange))
10. (all(guideline* N/2 utiliz*)) or (all(guideline* N/2 utilis*)) or (all(guideline* N/2 uptake)) or (all(guideline* N/2 transfer)) or (all(guideline* N/2 translat*)) or (all(guideline* N/2 transmit*)) or (all(guideline* N/2 transmission)) or (all(guideline* N/2 effectiveness)) or (all(guideline* N/2 populari*)) or (all(guideline* N/2 exchange)) or (all(guideline* N/2 synthes*)) or (all(guideline* N/2 transform*)) or (all(guideline* N/2 linkage*)) or (all(guideline* N/2 disseminat*)) or (all(guideline* N/2 implementation)) or (all(guideline* N/2 exchange))
11. all(Evidence N/2 informed)
12. (all(Quality N/2 assurance)) or (all(Quality N/2 improvement)) or (all(Quality N/2 service))
13. all("Best practice") or all("Best practices")
14. all(facilit* N/2 chang*)
15. S2 OR S3 OR S4 OR S5 OR S6 OR S7 OR S8 OR S9 OR S10 OR S11 OR S12 OR S13 OR S14
16. S1 AND S15

Medline (Inception – November 14, 2012)

1. exp nursing/
2. nursing.fs.
3. exp nurses/
4. education nursing, continuing/
5. (nurse educator$ or nursing educator$).mp.
6. or/1-5
7. (chang$ adj2 agent$).mp.
8. (coordinat$ adj2 chang$).mp.
9. (critical$ adj1 apprais$).mp.
10. Diffusion of innovation.mp.
11. exp decision making/
12. ((Knowledge or evidence or research or guideline:) adj2 (utiliz$ or > utilis$ or uptake or transfer or translat$ or transmit$ or > transmission or effectiveness or populari$ or exchange or synthes$ or > transform$ or linkage: or disseminat$ or implementation or > exchange)).mp.
13. (Evidence adj2 informed).mp. (525)
14. (Quality adj2 (assurance or improvement or service)).mp. (71725)
15. Best practice$.mp. (8658)
16. (facilit$ adj2 chang?).mp. (843)
17. or/7-16 (236759)
18. 6 and 17 (19760)
19. limit 18 to (english language and english) (17871)

Web of Science (SCI-EXPANDED, SSCI, A&HCI, CPCI-S, CPCI-SSH) (Inception – November 22, 2012)

1. (TI=nurs* OR TS=nurs*) AND Language=(English)
2. (TI=(chang* near/2 agent*) OR TS=(chang* near/2 agent*)) AND Language=(English)
3. (TS=(coordinat* near/2 chang*) or TI=(coordinat* near/2 chang*)) AND Language=(English)
4. (TS=(critical* near/1 apprais*) or TI=(critical* near/1 apprais*)) AND Language=(English)
5. (TS="diffusion of innovation" or TI="diffusion of innovation") AND Language=(English)
6. (TI="decision making" or TS="decision making" or TI="decision-making" or TS="decision-making" or TI=decisionmaking or TS=decisionmaking) AND Language= (English)
7. (TS=((Knowledge or evidence or research or guideline*) near/2 (utiliz* or utilis* or uptake or transfer or translat* or transmit* or transmission or effectiveness or populari* or exchange or synthes* or transform* or linkage* or disseminat* or implementation or exchange)) or TI=((Knowledge or evidence or research or guideline*) near/2 (utiliz* or utilis* or uptake or transfer or translat* or transmit* or transmission or effectiveness or populari* or exchange or synthes* or transform* or linkage* or disseminat* or implementation or exchange))) AND Language=(English)
8. (TS=(Evidence near/2 informed) OR TS=(Quality near/2 (assurance or improvement or service)) or TI=(Evidence near/2 informed) OR TI=(Quality near/2 (assurance or improvement or service))) AND Language=(English)
9. (TI="best practice" or TI="best practices" or TS="best practice" or TS="best practices") AND Language=(English)
10. (TI=(facilit* near/2 chang*) or TS=(facilit* near/2 chang*)) AND Language=(English)
11. #10 OR #9 OR #8 OR #7 OR #6 OR #5 OR #4 OR #3 OR #2
12. #10 OR #9 OR #8 OR #7 OR #6 OR #5 OR #4 OR #3 OR #2

EPOC Register (Inception – May 22, 2013)

1. (knowledge translation or knowledge transfer or KT or change agent* or agent* of change or coordinat* chang* or co-ordinat* chang* or diffusion of innovation) All indexed fields ((change and agent) or (change and coordinat) or (change and co-ordinat*) or (innovation AND diffusion) or knowledge transfer or knowledge translat) TITLE
2. Decision making all fields AND (nurse or nurses or nursing) all fields.

ProQuest Dissertations & Theses A&I (Inception – November 21, 2012)

S1. all(nursing) or all(nurse?)
S2. all(chang* N/2 agent*)
S3. all(coordinat* N/2 chang*)
S4. all(critical* N/2 apprais*)
S5. all("diffusion of innovation") or all("diffusion and innovation")
S6. all("decision making") or all("decision-making") or all("decision maker") or all("decision makers")
S7. ((all(Knowledge N/2 utiliz*)) or (all(Knowledge N/2 utilis*)) or (all(Knowledge N/2 uptake)) or (all(Knowledge N/2 transfer)) or (all(Knowledge N/2 translat*)) or (all(Knowledge N/2 transmit*)) or (all(Knowledge N/2 transmission)) or (all(Knowledge N/2 effectiveness)) or (all(Knowledge N/2 populari*)) or (all(Knowledge N/2 exchange)) or (all(Knowledge N/2 synthes*)) or (all(Knowledge N/2 transform*)) or (all(Knowledge N/2 linkage*)) or (all(Knowledge N/2 disseminat*)) or (all(Knowledge N/2 implementation)) or (all(Knowledge N/2 exchange))
S8. all((all(evidence N/2 utiliz*)) or (all(evidence N/2 utilis*)) or (all(evidence N/2 uptake)) or (all(evidence N/2 transfer)) or (all(evidence N/2 translat*)) or (all(evidence N/2 transmit*)) or (all(evidence N/2 transmission)) or (all(evidence N/2 effectiveness)) or (all(evidence N/2 populari*)) or (all(evidence N/2 exchange)) or (all(evidence N/2 synthes*)) or (all(evidence N/2 transform*)) or (all(evidence N/2 linkage*)) or (all(evidence N/2 disseminat*)) or (all(evidence N/2 implementation)) or (all(evidence N/2 exchange)))
S9. (all(research N/2 utiliz*)) or (all(research N/2 utilis*)) or (all(research N/2 uptake)) or (all(research N/2 transfer)) or (all(research N/2 translat*)) or (all(research N/2 transmit*)) or (all(research N/2 transmission)) or (all(research N/2 effectiveness)) or (all(research N/2 populari*)) or (all(research N/2 exchange)) or (all(research N/2 synthes*)) or (all(research N/2 transform*)) or (all(research N/2 linkage*)) or (all(research N/2 disseminat*)) or (all(research N/2 implementation)) or (all(research N/2 exchange))
S10. (TI=(facilit* near/2 chang*) or TS=(facilit* near/2 chang*)) AND Language=(English)
S11. (all(guideline* N/2 utiliz*)) or (all(guideline* N/2 utilis*)) or (all(guideline* N/2 uptake)) or (all(guideline* N/2 transfer)) or (all(guideline* N/2 translat*)) or (all(guideline* N/2 transmit*)) or (all(guideline* N/2 transmission)) or (all(guideline* N/2 effectiveness)) or (all(guideline* N/2 populari*)) or (all(guideline* N/2 exchange)) or (all(guideline* N/2 synthes*)) or (all(guideline* N/2 transform*)) or (all(guideline* N/2 linkage*)) or (all(guideline* N/2 disseminat*)) or (all(guideline* N/2 implementation)) or (all(guideline* N/2 exchange))
S12. all(Evidence N/2 informed)
S13. (all(Quality N/2 assurance)) or (all(Quality N/2 improvement)) or (all(Quality N/2 service))
S14. all("Best practice") or all("Best practices")
S15. all(facilit* N/2 chang*)
S16. S3 OR S4 OR S5 OR S6 OR S7 OR S8 OR S9 OR S10 OR S11 OR S12 OR S13 OR S14 or S15
S17. (all(nursing) OR all(nurse?)) AND (S3 OR S4 OR S5 OR S6 OR S7 OR S8 OR S9 OR S10 OR S11 OR S12 OR S13 OR S14 or S15)
