# Supplementary material for: The effectiveness of knowledge translation interventions for promoting evidence-informed decision-making among nurses in tertiary care: a systematic review and meta-analysis
Source: Implement Sci. 2015 Jul 14;10:98. doi: 10.1186/s13012-015-0286-1 (PMC4499897; doi:10.1186/s13012-015-0286-1)
Supplement: Additional file 2: — Screening criteria. This file provides full details of the title and abstract and full-text screening criteria. [file 13012_2015_286_MOESM2_ESM.pdf]

Additional file 2 – Screening criteria

**Title and Abstract**	• Is article in English?<br>• Does the study occur in an acute care/hospital setting?<br>• Does the study describe a KT intervention?<br>• Is the outcome about a) nurses' knowledge or skill for research use, b) nurses' research use (behaviour), c) client outcomes as a result of nurses' research use, or d) contextual factors for nurses' research use?
**Full-Text**	• Is article in English?<br>• Is the study design one of the following: systematic review, RCT, cluster RCT, non-randomized cluster controlled trials, controlled before and after studies, interrupted time series, mixed methods, qualitative?<br>• Does the study describe a KT intervention?<br>• Is the intervention applied in an acute care setting?<br>• Does article have data (qualitative or quantitative)?<br>• Is the outcome reported for a) change in nurses' knowledge or skills for research use, b) change in nurses' research use (behaviour), c) client outcomes as a result of nurses research use OR d) contextual information?<br>• Is the study a one-group no comparison, before/after study?
