# Supplementary material for: The effectiveness of knowledge translation interventions for promoting evidence-informed decision-making among nurses in tertiary care: a systematic review and meta-analysis
Source: Implement Sci. 2015 Jul 14;10:98. doi: 10.1186/s13012-015-0286-1 (PMC4499897; doi:10.1186/s13012-015-0286-1)
Supplement: Additional file 3: — Characteristics of included studies. This file provides the details of the characteristics of the included studies identified during data extraction. [file 13012_2015_286_MOESM3_ESM.pdf]

Additional file 3 – Characteristics of included studies

**Study**	**Authors**: Daly M, Kermode S, Reilly D [32]<br>**Date**: 2009<br>**Country:** Australia
**Objective**	To assess the impact of two different nurse education and training programs (self-directed vs traditional in-service) on screening and management of alcohol withdrawal.
**Theoretical Framework**	Malcolm Knowles – Adult Learning Theory
**Methods**	**Design**: Cluster non-randomized trial<br>**Sampling:** Three hospitals agreed to participate in self-directed learning intervention and eight hospitals in the in-service education intervention.<br>**Recruitment Methods:** Nurses recruited from across 8 hospitals in rural area health services in New South Wales.<br>**Unit of Allocation:** Hospital<br>**Unit of Analysis:** Individual (patient)<br>**Differences in Baseline Characteristics:** Not reported.<br>**Intention to Treat Analysis:** Not applicable (groups of clinicians or patients).
**Participants**	**Size of Eligible Population:** Not reported.<br>**Sample Size**<br>**Total:** 11 hospitals, N = 308 nurses; N = 340 medical records.<br>**Intervention Group #1 (Competency Program):** n = 70 nurses in 3 hospitals; n = 258 medical records in 3 hospitals.<br>**Intervention Group #2 (In-service Education):** n = 238 nurses in 8 hospitals; n = 82 medical records in 8 hospitals.<br>**Hospital Characteristics:** Rural hospitals in New South Wales.<br>**Description of Nurse Participants:**<br>**"Nurses":** 308<br>**Age:** Not reported.<br>**Years of Experience:** Not reported .
**Intervention Group #1**	**Type of Intervention:** Educational materials<br>**Evidence Based for Intervention:** New South Wales Detoxification Clinical Practice Guidelines.<br>**Description of the Recipients:** 70 nurses in 3 hospitals.<br>**Description of Deliverer:** Clinical Nurse Consultant (CNC) provided education for both programs (Intervention #1 and Intervention #2) with a Drug and Alcohol Clinical Nurse Specialist (CNS) assisting with Intervention #1 at one of the hospitals.<br>**Length/Duration:** Reported as "during 2005".<br>**Adherence/Fidelity:** Not reported.<br>**Description of the Intervention:** Referred to as the "Competency program." Self-paced training program consisting of a self-directed learning package, open-book exam, and individual competency assessment on the management of alcohol withdrawal.

**Intervention Group #2**	**Type of Intervention:** Educational meetings (in-person).<br>**Evidence Based for Intervention:** New South Wales Detoxification Clinical Practice Guidelines.<br>**Description of the Recipients:** 238 nurses in 8 hospitals.<br>**Description of Deliverer:** Clinical Nurse Consultant (CNC) provided education for both programs (Intervention #1 and Intervention #2).<br>**Length/Duration:** 12 months; four to six education sessions depending on size of the hospital.<br>**Adherence/Fidelity:** Not reported.<br>**Description of the Intervention:** Referred to as “In-service education” on the management of alcohol withdrawal. Four to six offered depending on the size of the hospital (six sessions offered in larger hospitals). The reviewers infer that the sessions are the same session offered multiple times.
**Data Collection**	**Data Collection:** Chart audit.<br>**Type of Measurement:** Subjective<br>**Description:** Medical record file audit tool was used to document the presence or absence of each of the nine standards included in the protocol.<br>**Interpretation of Direction:** If the standard was required and implemented it was scored ‘yes’, if the standards was required and implemented it was scored ‘no’, and if it was not required is was scored ‘n/a’.<br>**Reliability Details:** Not reported.<br>**Validity Details:** Not reported.
**Outcomes**	**Behaviour:** Compliance with protocol for the management of alcohol withdrawal.

**Study**	**Authors**: Davies B, Hodnett E, Hannah M, O-Brien-Pallas L, Pringle D, et al. [68]; Graham ID, Davies B, Nimrod C [61]<br>**Date**: 2004<br>**Country:** Canada
**Objective**	The objective of this study is to explore factors influencing the successful and unsuccessful introduction of an evidence-based fetal health surveillance guideline. More specifically the study explored the process by which the transfer of nursing guidelines into practice occurred at the three study hospitals and whether using an active approach with an interactive educational workshop designed to influence nurses' self-efficacy couple with hospital policy reviews, multidisciplinary meetings, rounds, and unit discussion would lead to more appropriate implementation of evidence-based fetal health surveillance.
**Theoretical Framework**	Ottawa Model of Research Use
**Methods**	**Design**: Mixed Methods<br>**Sampling:** Convenience [quantitative (hospitals), qualitative], Random (quantitative, patient charts)<br>**Recruitment Methods:** It is not reported how the four hospitals were recruited/selected. For the qualitative component, participants participated in focus group interviews on their units before and after their shifts, and at other times. For quantitative component patient charts were randomly selected, but it is not reported how.<br>**Unit of Allocation:** Hospital<br>**Unit of Analysis:** Hospital, Individual (patient)<br>**Differences in Baseline Characteristics:** Reported, similar.<br>**Intention to Treat Analysis:** Not applicable (groups of clinicians or patients).
**Participants**	**Size of Eligible Population:** 155 nurses<br>**Sample Size**<br>**Total:** Assumed 100% for quantitative data collection (charts of women who gave birth in Fall, 1995 or Fall, 1996). 40-50% of eligible population participated in the qualitative data collection.<br>**Intervention Group**: n = 132 nurses; n = 386 patient charts (pre-intervention), n = 390 patient charts (post-intervention).<br>**Control group:** 118 (nurses); n = 397 patient charts (pre-intervention), n = 393 patient charts (post-intervention)<br>**Hospital Characteristics:** Four hospitals (secondary and tertiary) in southeastern Ontario.<br>**Description of Nurse Participants:**<br>**Age:** Not reported.<br>**Years of Experience:** Not reported.
**Intervention**	**Type of Intervention:** Multifaceted: 1) Educational meetings, 2) Educational materials<br>**Evidence Based for Intervention:** The Society of Obstetricians and Gynecologists of Canada (SOGC) guidelines for intermittent continuous support.<br>**Description of the Recipients:** Nurses, educators and administrators

	**Description of Deliverer:** Primary investigator and member of Perinatal Partnership Program of Eastern and Southeastern Ontario (workshops)<br>**Length/Duration:** 9 months<br>**Adherence/Fidelity:** > 80% of nurses attended the workshops.<br>**Description of the Intervention:** Usual community wide approach (see below description) plus a tailored program. The tailored program consisted of an interactive educational workshop and hospital policy reviews, multidisciplinary meetings, rounds, and unit discussions. Four 8-hour interactive workshop (discussion of experiences, skill practice, case studies, videotapes, clinical decision making protocols and discussion of perceived barriers) with 14-35 nurses were held. Nurses also received an 85-page workbook.
**Control**	**Description of the Control:** Usual community-wide approach to new policy implementation.<br>**Description of the Recipients:** Nurses, educators and administrators<br>**Description of Deliverer:** Regional perinatal educational program, regional multidisciplinary subcommittee, and Perinatal Partnership Program of Eastern and Southeastern Ontario.<br>**Length/Duration:** Not reported.<br>**Adherence/Fidelity:** Not reported.<br>**Description of the Intervention:** The 2 control hospitals received the usual community-wide approach to new policy implementation coordinated by the regional perinatal educational program. This included the formation of a regional multidisciplinary subcommittee, newsletter publications and presentational at the annual conference of the Perinatal Partnership Program of Eastern and Southeastern Ontario.
**Data Collection**	**Method:** Chart audit<br>**Type of Measurement:** Subjective.<br>**Description:** To determine the proportion of women who received EFM, charts were randomly selected from all women who gave birth in an 8-week period using and adapted perinatal data collection form.<br>**Interpretation of Direction:** Assumed higher scores equal greater proportions of the outcome.<br>**Reliability Details:** Not reported.<br>**Validity Details:** Not reported.
	**Method:** Work-sampling approach.<br>**Type of Measurement:** Subjective.<br>**Description:** 2 nurse research assistants observed nurses activities and made instantaneous classifications of nurse behaviours according to a structured 24-item worksheet. Time for the observation were randomly selected (10-minute blocks, with 28 observations per day).<br>**Interpretation of Direction:** Assumed higher scores equal greater practice.<br>**Reliability Details:** Ratings were consistent (> 95%) between 2 research assistants of the same behaviours in practice sessions.<br>**Validity Details:** Not reported.
	**Method:** Interviews (focus group and personal) and organization documents.<br>**Additional Description:** Focus group interviews were conducted with nurses

	and personal interviews were conducted with educational and administrators. At the intervention hospitals interviews focused on perceptions of uptake, barriers and challenges encountered, and factors believed to be important in facilitating use of the recommendations. At the control hospital interviews focused on the perceptions of the factors that may have initially inhibited/facilitated the uptake of the recommendations. Labour and delivery unit documents (unit meeting minutes, memoranda to staff, and policy manual changes) were reviewed.
**Outcomes**	**Behaviour:** Time spent providing labour support (Note: No comparison between groups)<br>**Client Outcomes:** Receipt of EFM (Note: No comparison between groups)<br>**Contextual Factors**

Study	**Authors**: Day T, Wainwright S, Wilson-Barnett J [39]<br>**Date**: 2001<br>**Country:** United Kingdom
**Objective**	To investigate 1) to what extent intensive care unit nurses' knowledge and practice of endotracheal suctioning is based on research evidence and 2) the effectiveness of a research-based teaching intervention to improve intensive care unit nurses' knowledge and practice of endotracheal suctioning.
**Theoretical Framework**	None reported.
**Methods**	**Design**: RCT<br>**Sampling:** Random<br>**Recruitment Methods:** The sample consisted of 16 nurses of which four nurses per grade were selected at random.<br>**Unit of Allocation:** Individual (nurses)<br>**Unit of Analysis:** Individual (nurses)<br>**Differences in Baseline Characteristics:** Not reported.<br>**Intention to Treat Analysis:** Not applicable, 100% follow-up.
**Participants**	**Size of Eligible Population:** Not reported.<br>**Sample Size**<br>**Total:** N = 16 nurses<br>**Intervention Group**: n = 8 nurses<br>**Control group:** n **=** 8 nurses<br>**Hospital Characteristics:** Large intensive care unit.<br>**Description of Nurse Participants:**<br>**“Nurses":** n = 16<br>**Age:** Not reported<br>**Years of Experience:** Not reported
**Intervention Group**	**Type of Intervention:** Educational meetings (in-person, didactic, interactive).<br>**Evidence Based for Intervention:** Summary of research recommendations for suctioning based on review of the research literature.<br>**Description of the Recipients:** 8 nurses from an intensive care unit.<br>**Description of Deliverer:** An independent lecturer who was an expert in the subject areas carried out the teaching intervention.<br>**Length/Duration:** The education programme took place over a 2-hour period.<br>**Adherence/Fidelity:** Not reported.<br>**Description of the Intervention:** An educational teaching programme on endotracheal suctioning. A detailed plan, learning outcomes, and practice outcomes were identified. A variety of teaching methods were utilized, including both didactic and interactive approaches, and practical bedside demonstrations. Teaching took place in small groups.
**Control Group**	**Description of the Control:** Educational meeting (in-person, didactic, interactive).<br>**Description of the Recipients**: 8 nurses from an intensive care unit.<br>**Description of Deliverer:** An independent lecturer who was an expert in the subject area carried out the teaching intervention.<br>**Length/Duration:** The educational programme took place over 2 hours.

	**Adherence/Fidelity:** Not reported.<br>**Description of the Intervention:** Educational teaching programme on humidification for mechanical ventilation. A detailed plan, learning outcomes, and practice outcomes were identified. A variety of teaching methods were utilized, including both didactic and interactive approaches, and practical bedside demonstrations. Teaching took place in small groups.
**Data Collection**	**Method:** Structured observation.<br>**Type of Measurement:** Subjective.<br>**Description:** Demonstrated research-based endotracheal suctioning.<br>**Interpretation of Direction:** Assumed higher scores equal greater practice.<br>**Reliability Details:** Inter-rater: % agreement Kappa ranged from 0.54 (one item only) to 1.0 indicating acceptable level of agreement.<br>**Validity Details:** Instruments were distributed for appraisal to a range of senior intensive care unit practitioners who were unconnected to the study site, and other experts in the field. Instruments were amended accordingly.
**Outcomes**	**Behaviour:** Research based endotracheal suctioning techniques.

**Study**	**Authors**: Dykes PC, Carroll DL, Hurley A, Lipsitz S, Benoit A, Chang F, et al. [49]<br>**Date**: 2010<br>**Country:** United States
**Objective**	To investigate whether a fall prevention tool kit using health information technology decreases patient falls in hospitals.
**Theoretical Framework**	Not reported.
**Methods**	**Design**: Cluster RCT<br>**Sampling:** Convenience.<br>**Recruitment Methods:** Medical units in 4 hospitals with fall rates higher than the mean for the institution the year before were matched to units with similar fall rates and patient-days. Units were eligible if they had a match and were not involved in other performance improvement efforts specific to fall prevention. A total of 2 medical units for each hospital unit met these criteria. The study protocol and waiver of consent was approved. All patients admitted or transferred to selected units from Jan 1 2009 to Jun 30 2009 were included in the study.<br>**Unit of Allocation:** Unit<br>**Unit of Analysis:** Individual (patients)<br>**Differences in Baseline Characteristics:** Reported, non-significant.<br>**Intention to Treat Analysis:** Not applicable (groups of nurses or patients).
**Participants**	**Size of Eligible Population:** Not reported.<br>**Sample Size**<br>**Total:** N = 10264 patients<br>**Intervention group:** n = 5160 patients<br>**Control group:** n = 5104 patients<br>**Hospital Characteristics:** 4 hospitals in the Partners for HealthCare System in the Boston, Massachusetts area.<br>**Description of Nurse Participants:** Not reported.
**Intervention Group**	**Type of Intervention:** Multifaceted: 1) Clinical decision support system; 2) Mass media; 3) Automatic computer generated tailored plan.<br>**Evidence Based for Intervention:** Morse Falls Scale (MFS) and decision rules and interventions based on evidence from the literature.<br>**Description of the Recipients:** Not reported. Assume 1) clinicians on units receiving the intervention and 2) patients being cared for by clinicians on the intervention units.<br>**Description of Deliverer:** The study team developed toolkit for clinician. The computer automatically delivered plans to the clinicians. Assume clinicians caring for patients on the intervention units.<br>**Length/Duration:** Phase 4 over 6 months; January 1, 2009 to June 30, 2009.<br>**Adherence/Fidelity:** Adherence to daily MFS = 94%.<br>**Description of the Intervention:** In Phase 1 to 3 the following was conducted: qualitative inquiry to identify barriers and facilitators to fall risk communication and interventions, development of pilot fall prevention toolkit (FPTK), identification of the icons for the FPTK (with domain experts, end users, and an

	illustrator), recommended interventions were reviewed, tailored if needed, and approved, the FPTK printed a bed poster, a patient education handout, and a plan of care. During Phase 4, the MFS was completed by clinicians using the fall prevention tool kit. Evidence-based/feasible interventions were automatically selected and tailored by nurse based on knowledge of the patient. A tailored fall prevention plan is automatically generated by the fall prevention toolkit from the fall risk assessment. A tailored bed poster generated from the fall risk assessment automatically prints and is placed above the bed for all patients at risk.
**Control Group**	**Type:** Multifaceted: 1) Education meeting; 2) Mass media.<br>**Description of the Control:** Usual care related to fall prevention, including an educational program on fall risk assessment and prevention plus a generic "high risk for falls sign" above the patients bed for patients at high risk for falls.<br>**Description of the Recipients:** Not reported. Assume 1) clinicians on units in the control group and 2) patients being cared for by clinicians on the control units.<br>**Description of Deliverer:** Assume clinicians caring for patients on the control units.<br>**Length/Duration:** Not reported.<br>**Adherence/Fidelity:** Adherence to daily MFS =81%<br>**Description of the Intervention**: The MFS was complete by clinicians using existing paper or electronic forms. A generic "high risk for falls" sign was placed above beds for patients scoring > 45 on the MFS. The fall prevention plan was manually documented in a paper or electronic record.
**Data Collection**	**Method:** Event reporting system<br>**Type of Measurement:** Subjective<br>**Description:** Reporting of patient falls and injurious falls required at all hospitals and is routinely recorded in an event reporting system in all units by the clinician caring for the patient at the time of the fall.<br>**Interpretation of Direction:** Greater the number, the more of the outcome.<br>**Reliability Details:** Not reported.<br>**Validity Details:** Incidents are validated by unit managers and hospital quality personnel.
**Outcomes**	**Client:** 1) patient falls; 2) patient falls with injury

Study	**Authors**: Ellis I, Howard P, Larson A, Roberston J [58]<br>**Date**: 2005<br>**Country:** Australia
**Objective**	The aims of this study were to 1) explore the relative and combined importance of context and facilitation in the successful implementation of a new evidence-based clinical practice protocol and 2) examine the establishment of more lasting change to individuals and organizations that resulted in greater incorporation of the principles of evidence-based practice.
**Theoretical Framework**	Promoting Action on Research Implementation in Health Services (PARIHS) framework
**Methods**	**Design**: Qualitative (Descriptive)<br>**Sampling:** Convenience<br>**Recruitment Methods:** Rural hospitals that had joined The Joanna Briggs Institute (JBI) were offered the opportunity to host an on-site workshop. All six hospitals participating in the educational programme also participated in this study. Not reported how participants for first stage (pre-workshop) interviews were recruited. In the second stage (summative evaluation), participants completed evaluation forms and agreed to participate in follow-up interviews. For the third stage (follow-up), participants who agreed to be followed up were contacted by phone; some no longer worked at the hospitals and one participated who had attended was contacted on the recommendation of a friend and invited to participate in an interview.
**Participants**	**Size of Eligible Population:** 6 hospitals<br>**Sample Size**<br>**Total:** n = 16 pre-workshop interviews; n = 54 summative evaluation forms, n = 33 summative interviews; n = 23 follow-up interviews<br>**Hospital Characteristics:** Six hospitals in rural western Australia.<br>**Description of Nurse Participants:** Not reported.
**Intervention**	**Type of Intervention:** Multifaceted: 1) Educational meetings, 2) Follow-up support.<br>**Evidence Based for Intervention:** "Demystifying Evidence-Based Practice" educational program developed by the Western Australia Centre for Evidence-Based Nursing and Midwifery.<br>**Description of the Recipients:** Nurses who could attend the workshop at the six participating hospitals.<br>**Description of Deliverer:** Facilitator trained in evidence-based practice (EBP).<br>**Length/Duration:** 1-day (workshop).<br>**Adherence/Fidelity:** Not reported.<br>**Description of the Intervention:** Hospitals or units in a hospital agreeing to host a workshop had one or more senior nurses identify a relevant nursing issue which was discussed with the workshop facilitator. Prior to the workshop the facilitator devised a draft guideline on the issue using JBI materials and other systematic reviews. Workshops consisted of the facilitator giving lectures on EBP and change management and instruction on searching strategies. During the workshop the participants amended the draft guideline and the outcome of the workshop was a new clinical protocol.

**Data Collection**	**Method:** Telephone interviews and evaluation forms.<br>**Additional Description:** Pre-workshop interviews identified key elements of the practice context and culture and leadership and team processes. Summative evaluation forms and interviews provided feedback for qualitative improvement purposes and elicited information about group processed developed at the workshops, and commitment by individuals to implement the new protocol. Follow-up interviews elicited nurses' experiences of developing and using protocols within their practice setting, their understanding of the principles of EBP, and their own professional interests and aspirations.
**Outcomes**	**Contextual Factors**

**Study**	**Authors**: Fan J, Woolfrey K [45]<br>**Date**: 2006<br>**Country:** Canada
**Objective**	To determine whether the ordering of ankle or foot radiographs by triage nurses according to the Ottawa Ankle Rules would decrease the length of stay of patients with ankle or foot injuries.
**Theoretical Framework**	Not reported.
**Methods**	**Design**: RCT.<br>**Sampling:** Convenience.<br>**Recruitment Methods:** Adult patients who presented to a single urgent care department were screened for eligibility. Patients were included if there was a history of twisting trauma to the ankle or foot in the preceding seven days and they were >18 years old.<br>**Unit of Allocation:** Individual (patients).<br>**Unit of Analysis:** Individual (patients).<br>**Differences in Baseline Characteristics:** Reported, non-significant.<br>**Intention to Treat Analysis:** Not applicable (groups of nurses or patients).
**Participants**	**Size of Eligible Population:** 433 patients (232 met inclusion criteria).<br>**Sample Size**<br>**Total:** N = 124 patients.<br>**Intervention Group:** n = 62 patients.<br>**Control Group:** n = 62 patients.<br>**Hospital Characteristics:** A single academic urgent care department with 45,349 annual visits located in Hamilton, Ontario.<br>**Description of Nurse Participants:** Not reported.
**Intervention Group**	**Type of Intervention:** Multifaceted: 1) Educational meeting (assumed in-person); 2) Feedback sessions for two weeks after patient enrollment started; 3) Standardized form.<br>**Evidence Based for Intervention:** Ottawa Ankle Rules.<br>**Description of the Recipients:** Nurses who received the intervention and patients who were allocated to receive a physical assessment by a triage nurse who received the intervention using a standardized form detailing the Ottawa Ankle Rules (OAR).<br>**Description of Deliverer:** The investigators delivered feedback sessions to the nurses. Triage nurse who provided a physical assessment to patients allocated to the intervention.<br>**Length/Duration:** Training session lasted 1 hour, 2 weeks of feedback sessions; Intervention was delivered to patients for 3 months.<br>**Adherence/Fidelity:** Not reported.<br>**Description of the Intervention:** All triage nurses received a one-hour training session on the study protocol and use of the OAR and then completed a standardized form detailing the OAR.. Investigators provided feedback sessions for two weeks after patient enrollment to correct any misconceptions. Investigators were available at all times to clarify any issues throughout the duration of the study.

**Control Group**	**Description of the Control:** Physical assessment conducted by an emergency physician.<br>**Description of the Recipients:** Patients allocated to receive the control.<br>**Description of Deliverer:** Emergency physicians.<br>**Length/Duration:** 3 months (July to September 2004)<br>**Adherence/Fidelity:** Not reported.
**Data Collection**	**Method:** Event reporting system.<br>**Type of Measurement:** Subjective.<br>**Description:** A single investigator reviewed medical records to record ED process times.<br>**Interpretation of Direction:** Greater the number, the more of the outcome.<br>**Reliability Details:** Not reported.<br>**Validity Details:** Incidents are validated by unit managers and hospital quality personnel.
**Outcomes**	**Client:** Length of stay

**Study**	**Authors**: Gifford WA, Davie B, Edwards N, Graham ID [47]; Edwards N, Davies B, Ploeg J, Dobbins N, Skelly J, Griffin P, Ralphs-Thibodeau S [51]<br>**Date**: 2006<br>**Country:** Canada
**Objective**	The research objectives were (1) to identify leadership behaviours and activities associated with organizations that sustained the implementation of clinical practice guidelines and (2) to generate a theoretical model of leadership that enables nurses to use clinical guidelines in practice
**Theoretical Framework**	Strauss and Corbin
**Methods**	**Design**: Qualitative (Grounded theory).<br>**Sampling:** Purposeful sampling.<br>**Recruitment Methods:** The 12 organizations who were using the Registered Nurses Association of Ontario (RNAO) Best Practice Guidelines (BPGs) were invited to participate in the primary study and nine agreed. How these agencies were recruited is not reported. How participants were recruited to participate in data collection methods is not reported.
**Participants**	**Size of Eligible Population:** 9 out of 12 hospitals participated.<br>**Sample Size:** 9 hospitals (4 sustained CPG; 5 non sustained) and 32 individual interviews.<br>**Hospital Characteristics:** Organizations (long-term care, complex continuing, rehabilitation, acute care, teaching facility) in Ontario, Canada ranging in size from approx. 150-750 beds.<br>**Description of Nurse Participants:**<br>**Staff Nurses:** n = 15<br>**Clinical Resource Nurses:** n = 11<br>**Administrators (Directors of Nursing, Directors of Care, Administrators, Managers):** n = 6<br>**Professional License:** n = 20<br>**RN/RPN:** n = 2<br>**Age:** Not reported.<br>**Years of Experience:** Not reported.
**Intervention**	**Type of Intervention:** Multifaceted: 1) Educational materials (printed); 2) Educational meetings, 3) Knowledge broker (Clinical Resource Nurse), 4) Other (partial funding for knowledge broker for six to nine months).<br>**Evidence Based for Intervention:** RNAO BPGs (Risk Assessment and Prevention of Pressure Ulcers, Prevention of Falls and Fall Injuries in the Older Adult, Promoting Continence Using Prompted Voiding, Prevention of Constipation in the Older Adult Population).<br>**Description of the Recipients:** Assume staff nurses, clinical resource nurses administrators, professional license, and RN/RPNs.<br>**Description of Deliverer:** Clinical resource nurses, nursing managers, and administrators.<br>**Length/Duration:** 1 to 3 years.<br>**Adherence/Fidelity:** Not reported.<br>**Description of the Intervention:** Guidelines were implemented as part of the

	RNAO BPG project, a multi-year initiative to develop and evaluate nursing clinical guidelines in the province of Ontario, Canada. Participating organizations were provided with partial funding for six to nine months for a CRN to lead implementation, a formal launch organized by the RNAO, an orientation workshop for managers, senior administrators, and CRNs that focused on implementation strategies and evaluation processes, and pre/post evaluations for each BPG. CRNs worked with key stakeholders at the organizations to develop implementation strategies. Monthly teleconferences with the CRNs, a member of each of the BPG development panels, and the program manager from RNAO to share strategies for BPG implementation.
**Data Collection**	**Method:** Secondary analysis of qualitative data.<br>**Additional Description:** Data sources consisted of individual semi-structured interviews, group interviews and written summary reports, organizational documents, and group telephone interviews with administrators, clinical resource nurses, or both.
**Outcomes**	**Contextual Factors**

**Study**	**Authors**: Girouard S [34]<br>**Date**: 1978<br>**Country:** United States
**Objective**	To measure the effectiveness of the clinical specialist employing a strategy of planned change on nurses engagement in preoperative teaching.
**Theoretical Framework**	"Havelock (1976) provides a theoretical model for combining the definitions and functions of clinical specialists discussed in the nursing literature. In formulating the concept of linkage, Havelock united various theoretical propositions relating to change, into the concept of linkage. The model focuses on the user as problem solver. Change is facilitated by an outside resource (the change agent). The change agent links the user to the outside resources necessary for problem solving" (p. 59).
**Methods**	**Design**: RCT.<br>**Sampling:** Convenience.<br>**Recruitment Methods:** Not reported.<br>**Unit of Allocation:** Unit; one of the units was randomly selected to be the intervention unit and the other the control unit.<br>**Unit of Analysis:** Individual (Nurse, Patient).<br>**Differences in Baseline Characteristics:** Reported, Non-significant (nurses).<br>**Intention to Treat Analysis:** Not Applicable (groups of clinicians or patients).
**Participants**	**Size of Eligible Population:** Not reported.<br>**Sample Size**<br>**Total:** N = 2 surgical units, N = 36 nurses; N = 80 patients.<br>**Intervention Group**: n= 20 nurses; n = 40 patients (n = 20 before, n = 20 after).<br>**Control Group:** n = 16 nurses; n = 40 patients (n = 20 before, n = 20 after).<br>**Hospital Characteristics:** Not reported.<br>**Description of Nurse Participants:**<br>**"Licensed Nurses":** 36 licensed nurses.<br>**Age in years, mean (SD):** Intervention group: 39.25 (9.47); Control group: 38.12 (11.75).<br>**Years of Experience, mean (SD):** Intervention group: 14 (8.98); Control group: 11.12 (10.48).
**Intervention Group**	**Type of Intervention:** Multifaceted: 1) Knowledge Broker; 2) Educational Meetings (in-person, didactic).<br>**Evidence Based for Intervention:** Clinical specialist supporting nurses to perform pre-operative teaching (linked to evidence for pre-operative teaching).<br>**Description of the Recipients: S**taff nurses in one medical-surgical unit.<br>**Description of Deliverer:** Clinical specialist functioning as a resource person (change agent).<br>**Length/Duration:** 4 weeks.<br>**Adherence/Fidelity:** Not reported.<br>**Description of the Intervention:** 1. A formal class and discussion to fill knowledge gaps was held. Topics included were principles of learning, the benefits of preoperative teaching, research findings in relation to preoperative

	teaching, content for preoperative teaching, and methods for doing preoperative teaching. 2. Written resources, mainly journal articles relating to preoperative teaching, were made available to the staff. 3. Example setting (role model) was done by the clinical specialist to demonstrate patient teaching, the writing of care plans, and the writing of nursing notes about preoperative teaching. This activity took place during the first two weeks of the intervention. Using the kardex teaching plan, the clinical specialist taught most of the patients who were to have surgery and charted her activities. Information about particular concerns or problems of the patients was shared with the nurse caring for the patient. 4. The development and implementation of a preoperative teaching plan for the kardex was done. This tool was to be used as a guide for bedside teaching and charting. The kardex forms were used by the clinical specialist during the first two weeks of the intervention. They were then given to the head nurse for use by the nursing staff. The unit clerk included the kardex form as part of the surgical patients chart.
**Control Group**	**Description of the Control:** No participation in the intervention.<br>**Description of the Recipients**: **S**taff nurses in one medical-surgical unit.<br>**Description of Deliverer:** Not applicable.<br>**Length/Duration:** 4 weeks.<br>**Adherence/Fidelity:** Not applicable.
**Data Collection**	**Method:** Patient teaching activity questionnaire.<br>**Type of Measurement:** Self report.<br>**Description:** Questionnaire; items related to professed preoperative teaching activities and documentation of them.<br>**Interpretation of Direction:** Theoretical range of scores is from 28-140. This higher the score, the more often the respondent said they participated in the activity.<br>**Reliability Details:** Not reported.<br>**Validity Details:** Not reported.
	**Method:** Interviews<br>**Type of Measurement:** Subjective.<br>**Description:** Patient report of the number of items taught preoperatively.<br>**Interpretation of Direction:** Increased score indicated more documented preoperative teaching.<br>**Reliability Details:** Not reported.<br>**Validity Details:** Not reported.
**Outcomes**	**Behaviour:** Preoperative teaching.

Study	**Authors**: Happell B, Johnston L, Hill C [54]<br>**Date**: 2003<br>**Country:** Australia
**Objective**	The Clinical Research Fellowship (CRF) program was developed to assist nurses to change practice on the basis of high-quality research evidence. This paper presents the results of a qualitative study examining the experiences of four CRF participants and three of their unit managers<br>in completing the program and implementing changes within the clinical setting.
**Theoretical Framework**	Not reported.
**Methods**	**Design**: Qualitative (Descriptive)<br>**Sampling:** Convenience sampling<br>**Recruitment Methods:** The participants in this study were mental health nurses who completed the Clinical Research Fellowship (CRF) program during 2001, and their unit managers. It is not reported how participants were recruited/selected to participate in the CRF program. Letters were sent to the CRF participants and their unit managers inviting them to participate in the study, and informing them that a follow-up telephone call would be made by the research assistant unless they requested that this not occur.
**Participants**	**Size of Eligible Population:** 4 mental health nurses completing CRF in 2001 and their 4 unit managers.<br>**Sample Size:** N = 7 (4 mental health nurses completing the CRF in 2001 and 3 unit managers)<br>**Hospital Characteristics:** Not reported.<br>**Description of Nurse Participants:** Not reported.<br>**Age:** Not reported.<br>**Years of Experience:** Not reported.
**Intervention**	**Type of Intervention:** Multifaceted: 1) Educational meetings (in-person, didactic, and interactive), 2) Educational materials<br>**Evidence Based for Intervention:** The Clinical Research Fellowship (CRF) program developed by the Victorian Centre for Nursing Practice Research Victoria, Australia.<br>**Description of the Recipients:** Mental health nurses enrolled in the CRF.<br>**Description of Deliverer:** Assumed to be Victorian Centre for Nursing Practice Research.<br>**Length/Duration:** 10 weeks.<br>**Adherence/Fidelity:** Note reported.<br>**Description of the Intervention:** The CRF program is designed to support clinically based nurses in identifying, appraising and utilizing research that will serve as an evidential base for their daily clinical practice. After identifying a practice, policy, or procedure within their own work environment that does not have a known evidential base, the first 2-weeks of the program are full-time, intensive where participants learn skills necessary to find and appraise research evidence. This includes plenary sessions held each day, which are augmented by small group tutorials designed to teach critical appraisal skills. Participants

	also have access to a number of unique databases and evidence-based practice Internet and are provided an interactive CD-ROM and workbooks developed by the Critical Appraisal Skills Program (CASP). An additional 8 weeks part-time supports the clinician requires to undertake the implementation of findings into practice in their clinical agency. Each participant is required to conduct a teaching session for other participants on an aspect of evidence-based practice. The Program concludes with a Seminar Day where each participant presents the results of his/her project to colleagues, university staff, and nurse unit managers.
**Data Collection**	**Method:** Semi-structured interviews.<br>**Additional Details:** An interview guide was prepared to encourage participants to discuss the following: their experiences of the CRF program; the process of implementing best practice following the completion of the CRF; the response of other staff; any resulting changes to nursing practice; and whether they would recommend the program to others. Participants were also encouraged to discuss other issues they considered pertinent.
**Outcomes**	**Contextual Factors:**

**Study**	**Authors**: Happell B, Martin T [55]<br>**Date**: 2005<br>**Country:** Australia
**Objective**	The aim of the current study is to evaluate the implementation of a number of Nursing Clinical Development Unit (NCDU)s within the mental health setting in Victoria, Australia. The primary aim of this initiative was to support and enhance the ability of clinical nursing leaders to develop the framework for cultural change based on the contribution of psychiatric nursing to improved health outcomes for service users.
**Theoretical Framework**	Not reported.
**Methods**	**Design**: Qualitative (Descriptive).<br>**Sampling:** Convenience sampling.<br>**Recruitment Methods:** Not reported how organization was recruited/selected. All participants from the first two years of the Nursing Clinical Development Unit (NCDU) Program were contacted in writing. They were informed that an impact evaluation was being conducted and advised that they would be contacted by telephone and requested to participate in an interview. They were asked to contact the CPNRP if they did not wish to be involved in the program. A number of participants could not be contacted as they had changed employment or were on periods of extended leave.
**Participants**	**Size of Eligible Population:** 25 nurses<br>**Sample Size**<br>**Total:** N = 14 nurses.<br>**Hospital Characteristics:** Only location (Melbourne, Australia) is reported.<br>**Description of Nurse Participants:** Not reported.<br>**Age:** Not reported.<br>**Years of Experience:** Not reported.
**Intervention**	**Type of Intervention:** Educational meetings<br>**Evidence Based for Intervention:** The Nursing Clinical Development Unit (NCDU) Program developed by the Centre for Psychiatric Nursing Research and Practice (CPNRP), in Melbourne, Australia.<br>**Description of the Recipients:** Nurse participating in NCDU program.<br>**Description of Deliverer:** Assumed the CPNRP at the University of Victoria.<br>**Length/Duration:** 2 years<br>**Adherence/Fidelity:** Not reported.<br>**Description of the Intervention:** The implementation of the NCDU program was based on the implementation strategy adopted by the University of Western Sydney. It is described as six 2-day workshops held over 6 months. The NCDU support program was funded and supported by the CPNRP. Topics covered in the inaugural program included: leadership and management, clinical research, professional development, information technology, and dissemination of information.
**Data Collection**	**Method:** Face-to-face interviews.<br>**Additional Description:** The interview questions related to what the experience of being an NCDU meant to the clinical environment, notable

	changes occurring as a result of the NCDU process, the positive and negative aspects of the NCDU approach, factors and resources that assisted with the development of the NCDU, any identified barriers to implementation, and how (if at all) these barriers have been overcome/reduced.
**Outcomes**	**Contextual Factors**

Study	**Authors**: Hyndman K [40]<br>**Date**: 2005<br>**Country:** Canada
**Objective**	To evaluate a dissemination intervention to enhance registered nurses' use of clinical practice guidelines (CPGs) for treating tobacco use and dependence.
**Theoretical Framework**	Rogers' Innovation Diffusion Theory and Bandura's Social Cognitive Theory.
**Methods**	**Design**: Cluster non-randomized trial.<br>**Sampling:** Convenience.<br>**Recruitment Methods:** Nurses employed full time, part time, or casual (at least 60 hours in the month prior to the study) who worked in antepartum care, perinatal assessment unit, labour and delivery units, labour, delivery, recovery, and postpartum, and postpartum care were invited to participate.<br>**Unit of Allocation:** Hospital.<br>**Unit of Analysis:** Individual (nurses).<br>**Differences in Baseline Characteristics:** Reported, non-significant.<br>**Intention to Treat Analysis:** Not reported/unable to determine.
**Participants**	**Size of Eligible Population:**147 nurses from the intervention and 142 nurses from the control hospital were invited to participate.<br>**Sample Size**<br>**Total:** N = 138 nurses<br>**Intervention Group #1**: n = 67 nurses<br>**Intervention Group #2:** n = 71 nurses<br>**Hospital Characteristics:** Two large health centres in the Winnipeg Regional Health Authority.<br>**Description of Nurse Participants:**<br>**"Nurses":** n = 138.<br>**Age in years, mean (SD):** Overall 38.8 (9.9) with a range from 22-62, Intervention: 8.7 (10.2); Control: 38 (9.6).<br>**Years of Experience, mean (SD):** Intervention: 13 (10.3); Control: 13 (9.9).
**Intervention Group #1**	**Type of Intervention:** Multifaceted: 1) Educational meetings (in-person, formal/informal); 2) Educational materials (printed/electronic); 3) Mass media.<br>**Evidence Based for Intervention:** Clinical practice guidelines included: Agency for Health Care Research and Quality reference guide for treating tobacco use and dependence, the Canadian Nurses Association guidelines for nurses working with Canadian affected by tobacco, and the Canadian Cancer Society pamphlet on helping smokers quit.<br>**Description of the Recipients:** Nurses working in a variety of units of one hospital.<br>**Description of Deliverer:** Delivered by the researcher and a trained research assistant who was also an experienced registered nurse.<br>**Length/Duration:** 10 weeks.<br>**Adherence/Fidelity:** Not reported.<br>**Description of the Intervention:** One-on-one brief, educational visits supplemented with a self-study package of a video and print materials.

	The self-study package contained the CPGs, information on the smoking prevalence in Manitoba, the effectiveness of nurse-led smoking cessation interventions, the stages in the smoking cessation process, the adverse health effects of smoking to the women and the infant, and patient education pamphlets. A 15-minute video highlighted printed information with four vignettes demonstrating the application of the CPGs with simulated patients. Educational outreach visits reinforced what the nurses were expected to implement and document and gave feedback on what constituted good performance via one-on-one personal interaction, verbal encouragement and praise, opportunities to practice interventions with the researcher/research assistant providing the visits. A detailed visit guide and minute by minute framework outlining research activities was developed for consistency during the visits. The first visit was planned within two weeks after the distribution of the self-study package. Each nurse was visited twice - once at approximately two weeks and again approximately three to four weeks after the first visit. Written information about the CPGs contained in a poster display was available on the study units for nurses during recruitment in the months of August and September 2003.
**Intervention Group #2**	**Type of Intervention:** Multifaceted: 1) Educational meetings (in-person, formal/informal); 2) Mass media.<br>**Evidence Based for Intervention:** CPGs included: Agency for Health Care Research and Quality reference guide for treating tobacco use and dependence, the Canadian Nurses Association guidelines for nurses working with Canadian affected by tobacco, and the Canadian Cancer Society pamphlet on helping smokers quit.<br>**Description of the Recipients:** Nurses working in a variety of units of one hospital.<br>**Description of Deliverer:** Researcher.<br>**Length/Duration:** Meetings lasted 5-10 minutes and the brief informal sessions lasted 3-5 minutes. Assume the entire duration was 3 months (July to September 2003).<br>**Adherence/Fidelity:** Not reported.<br>**Description of the Intervention:** Hospital nurses in received verbal information about the CPGs during awareness sessions held from July to September 2003. Written information about the CPGs contained in a poster display was available on the study units for nurses during recruitment in the months of August and September 2003. Academic detailing sessions and smoking cessation interventions record (SCIR) forms were not implemented at the control hospital. After all post intervention surveys were returned, each nurse at the control hospital received the same self-study materials and had the opportunity to attend an educational session provided by the researcher.
**Data Collection**	**Method:** Cooke Scale<br>**Type of Measurement:** Self-report<br>**Description:** Nurses' adherence to the CPG was assessed by the number of different types of smoking cessation interventions used. Nurses were asked to recall the last 10 women smokers they cared for in the hospital and to estimate

	how many women received smoking cessation interventions. In relation to the last 10 smokers, nurses were asked to indicate how often they offered each smoking cessation intervention.<br>**Interpretation of Direction:** The items were scored using a five point frequency-based Likert scale from never (0 smokers out of 10) to usually (9-10 smokers out of 10). Responses were summed for each nurse resulting in a minimum adherence score of 12 and a maximum score of 60. Higher score reflect greater adherence.<br>**Reliability Details:** Cronbach's alpha for adherence to CPGs was 0.88.<br>**Validity Details:** The 12 items in the Cooke Scale (revised) were analyzed using factor analysis for this study. The factor structure was clear and all items addressed aspects of adhering to the CPGs on treating tobacco use and dependence.
**Outcomes**	**Behaviour:** Nurse adherence to CPG**.**

Study	**Authors**: Kajermo KN, Nordström G, Krusebrant A, Lützén K [56]<br>**Date**: 2001<br>**Country:** Sweden
**Objective**	This paper explores nurses' reactions on their experiences of disseminating and implementing research findings in clinical practice within the framework of an educational programme.
**Theoretical Framework**	Lewin's change process, quality improvement principles.
**Methods**	**Design**: Qualitative (Descriptive)<br>**Sampling:** Convenience.<br>**Recruitment Methods:** Not reported how organization recruited/selected. Of the 10 nurses purposively selected by head nurses to participate in the educational programme, they were divided into two groups of five nurses for qualitative data collection.
**Participants**	**Size of Eligible Population:** 10 nurses.<br>**Sample Size:** 10 nurses<br>**Hospital Characteristics:** Not reported.<br>**Description of Nurse Participants:**<br>**RN:** n =10<br>**Age:** Not reported.<br>**Years of Experience:** range 2 to 23 years
**Intervention**	**Type of Intervention:** Educational meetings (in-person, didactic, interactive)<br>**Evidence Based for Intervention:** Not reported.<br>**Description of the Recipients:** 10 nurses purposively selected to participate in the Research Nurse Intern Program.<br>**Description of Deliverer:** Activities were carried out under the guidance of the course leaders (two of the investigators) in collaboration with other nurse researchers.<br>**Length/Duration:** 2 years.<br>**Adherence/Fidelity:** Not reported.<br>**Description of the Intervention:** As part of project to facilitate the integration of research into practice and professional development of staff nurses, an educational program (Research Nurse Intern Programme) was introduced in 1994. Unit-based activities during the first year included creating a research corner for poster presentations of research findings and arranging research seminars. Unit-based activities during the second year included planning, implementing and evaluating a research-based innovation. Overall, the part-time programme covered the research process, literature searches, research methods, the reviewing and critical evaluation of research articles, the technique of developing a poster, the relationship between research and quality improvement and the process of change. Formal classes lasting usually 2.5 hours were held once a month on these topics.
**Data Collection**	**Method:** Focus groups<br>**Additional Description:** Focus groups, moderated by two of the invetigators, started 6 months after the initiation of the educational programme and continued throughout its implementation. The focus groups started by asking

	about situations/experiences that were an example of barriers to or facilitators of their activities in the dissemination and implementation of research findings. Follow-up questions were posed, e.g. `What were the comments from your colleagues on the research seminar?' or `Have you got any responses to or comments on your proposal?'.
**Outcomes**	**Contextual Factors**

**Study**	**Authors**: Kirshbaum M [33]<br>**Date**: 2007<br>**Country:** England
**Objective**	To evaluate the effect of a targeted booklet, Exercise and Breast Cancer: A Booklet for Breast-Care Nurses, on changes in knowledge, reported practice, and attitudes of breast care nurses (BCN) in the United Kingdom.
**Theoretical Framework**	Specific attributes and characteristics of the experimental dissemination method (the intervention) were derived from the results of (1) a national survey of barriers to research utilisation of breastcare nurses and (2) the development of a conceptual framework used for selecting a targeted intervention.
**Methods**	**Design**: Cluster RCT.<br>**Sampling:** Convenience .<br>**Recruitment Methods:** Subset from the national population of BCNs approached previously in a national survey.<br>**Unit of Allocation:** Hospital; Hospitals were categorized as general or specialty and then randomization was stratified by hospital type. All nurses working at the same hospital were allocated to the same group.<br>**Unit of Analysis:** Hospital (nurses clustered within hospitals)<br>**Differences in Baseline Characteristics:** Reported, significant<br>**Intention to Treat Analysis:** No
**Participants**	**Size of Eligible Population:** Not reported.<br>**Sample Size**<br>**Total:** 137 nurses participated who received the baseline questionnaire.<br>**N =** 112 responded and were randomized.<br>**Intervention Group:** n = 56.<br>**Control Group:** n = 46.<br>**Hospital Characteristics:** General and specialist hospitals in Northern England.<br>**Description of Nurse Participants:**<br>**RN:** Breast Care Nurses (BCNs)<br>**Age:** Not reported<br>**Years of Experience:** as a BCN:<br>< 2 years, n = 3; 2 to 4 years, n= 18; 5 to 10 years, n = 20; > 10 years, n = 10.
**Intervention Group**	**Type of Intervention:** Educational Materials (Printed).<br>**Evidence Based for Intervention:** A critical review was undertaken to identify, assess, and synthesize empirical data about breast cancer and physical exercise. A panel of experts working in the field of breast cancer, including a surgeon, clinical psychologist, and lecturer in cancer nursing were asked to review and confirm clinical accuracy of the booklet. Recommendations from these experts were incorporated into the final version, which consisted of 18 pages of text, 6 pages of references and a 3-page table that showed details of 18 empirical studies on the benefits of exercise for breast-cancer patients"<br>**Description of the Recipients:** BCNs.<br>**Description of Deliverer:** Participants were sent the booklet by mail.<br>**Length/Duration:** 2 months.

	**Adherence/Fidelity:** Not reported.<br>**Description of the Intervention:** The booklet indicated the special experience of the target group (BCNs), had professional terminology, and indicated in the title that it was intended for the use of BCNs. The booklet began with a summary of the physical and psychological needs of breast-cancer patients and the text was structured into eight sections: Introduction, The Challenges of Breast Cancer, The Benefits of Exercise, What Type of Exercise is Best?, Implications for Nursing Practice, Summary and Implications, References, and Table of Empirical Studies.
**Control Group**	**Description of the Control:** Eight weeks after the post-intervention data was collected, a copy of the booklet was mailed to all nurses assigned to the control group for information and use.<br>**Description of the Recipients**: BCNs.<br>**Description of Deliverer:** Participants were sent the booklet by postal mail.<br>**Length/Duration:** Not applicable.<br>**Adherence/Fidelity:** Not reported.<br>**Description of the Content Provided:** See description above.
**Data Collection**	**Method:** Exercise and Breast Cancer Questionnaire.<br>**Type of Measurement:** Self report.<br>**Description:** How often BCNs would recommend exercise to their patients for each of 12 common quality of life problems.<br>**Interpretation of Direction:** Higher score indicates less practice; 1 = always to 5 = never.<br>**Reliability Details:** Not reported.<br>**Validity Details:** Content and face validity was established via a systematic literature search and consensus of experts in related disciplines.
**Outcomes**	**Behaviour:** Reported practice (recommendations of exercise according to the booklet).

**Study**	**Authors**: Lewicki LJ [43]<br>**Date**: 1997<br>**Country:** United States
**Objective**	To examine if feedback given to nurses on their performance for assessing pressure ulcer risk from case studies, combined with guideline dissemination and education, increased the use of pressure ulcer prevention guidelines in their practice.
**Theoretical Framework**	Newman's Systems model and Rogers Diffusion of Innovation.
**Methods**	**Design**: RCT<br>**Sampling:** Random.<br>**Recruitment Methods:** The sample chosen was chosen from 10 nursing units with medium pressure ulcers rates. The units were placed in random order and each of the 206 RNs who met inclusion criteria assigned a number and 192 RNs were randomly selected. The researcher then approached each RN individually to explain the study and obtain informed consent.<br>**Unit of Allocation:** Individual (nurses).<br>**Unit of Analysis:** Individual (nurses).<br>**Differences in Baseline Characteristics:** Reported, non-significant.<br>**Intention to Treat Analysis:** Not applicable (groups of clinicans or patients).
**Participants**	**Size of Eligible Population:** 206 nurses<br>**Sample Size**<br>**Total:** N = 110 nurses (96 at baseline)<br>**Intervention Group #1:** n = 32 nurses (at baseline)<br>**Intervention Group #2:** n = 35 nurses (at baseline)<br>**Control Group:** n = 29 nurses (at baseline)<br>**Hospital Characteristics:** 29 nursing units (excluding Pediatric, Psychiatry/Mental health, Recovery Room, Operating Room) in a teaching hospital in Cleveland, Ohio. The 10 nursing units chosen included: Rehabilitation, Internal Medicine/Hypertension/Nephrology, Neuroscience, Neuroscience Intensive Care, Vascular Medicine and Surgery, Colorectal Surgery, Internal Medicine. The unit size of the 10 nursing units ranged from 8 to 36 beds.<br>**Description of Nurse Participants:** Note: At baseline.<br>**RN:** Overall: 100%<br>**Age in years, mean (SD):** Intervention #1: 35.16 (8.33); Intervention #2: 32.37 (9.66);; Control: 30.97 (9.79)<br>**Years of Experience, mean (SD):** Intervention #1: 8.13 (8.07); Intervention #2: 7.31 (7.26); Control: 5.97 (6.57)
**Intervention Group #1**	**Type of Intervention:** Multifaceted: 1) Educational materials (printed); 2) Educational meeting; 2) individual feedback on case studies.<br>**Evidence Based for Intervention:** Department of Health and Human Services AHCPR Clinical Practice Guideline# 3.<br>**Description of the Recipients:** Nurses who received the AHCPR guideline, an in-service on the content of the guideline, and the individual feedback intervention.

	**Description of Deliverer:** Not reported.<br>**Length/Duration:** 2 months for enrollment, guideline distribution, and in-services. 3 months for individual feedback intervention.<br>**Adherence/Fidelity:** 16% (n = 5) of participants read the guideline and 16% (n = 5) attended the in-service on the guideline.<br>**Description of the Intervention:** Participants received a copy of the guideline and an in-service explaining the guideline's recommendation. The individual feedback intervention consisted of returning the nurses completed six sets of four case studies that described a patient situation and contained information to rate the risk for pressure ulcer development using the Braden Scale for Predicting Pressure Score Risk to the nurses along with the responses and rationale that the experts have given. Participants were awarded 1.1 contact units for attending the in-service in which they received a copy of the guideline. A total of 61 in-services on all three shifts (days, evenings, nights) and among all 10 study units were conducted to accommodate all subjects. Feedback consisted giving the nurses a set of four case studies every other week. On weeks that the case studies were not completed, feedback on performance on the case studies was given
**Intervention Group #2**	**Type of Intervention:** Multifaceted: 1) Educational materials (printed); 2) Educational meeting; 2) Group feedback on case studies.<br>**Evidence Based for Intervention:** Department of Health and Human Services AHCPR Clinical Practice Guideline# 3 and the Braden Scale for Predicting Pressure Score Risk.<br>**Description of the Recipients:** Nurses who received the AHCPR guideline, an in-service on the content of the guideline, and the group feedback intervention.<br>**Description of Deliverer:** Not reported.<br>**Length/Duration:** 2 months for enrollment, guideline distribution, and in-services. 3 months for group feedback intervention.<br>**Adherence/Fidelity:** 17% (n = 6 read the guideline and 6% (n = 2 attended the in-service on the guideline.<br>**Description of the Intervention:** Participants received a copy of the guideline and an in-service explaining the guideline's recommendation. The group feedback intervention consisted of a report on the percentage of the group that agrees with the expert's scores and rationale on six sets of four case studies that described a patient situation and contained information to rate the risk for pressure ulcer development using the Braden Scale for Predicting Pressure Score Risk. Participants were awarded 1.1 contact units for attending the in-service in which they received a copy of the guideline. A total of 61 in-services on all three shifts (days, evenings, nights) and among all 10 study units were conducted to accommodate all subjects. Feedback consisted giving the nurses a set of four case studies every other week. On weeks that the case studies were not completed, feedback on performance on the case studies was given.
**Control Group**	**Description of the Control:** Multifaceted: 1) Educational materials (printed); 2) Educational meeting.<br>**Description of the Recipients:** Nurses who received the AHCPR guideline and an in-service on the content of the guideline.

	**Description of Deliverer:** Not applicable.<br>**Length/Duration:** 2 months for enrollment, guideline distribution, and in-services.<br>**Adherence/Fidelity:** 14% (n = 4) read the guideline and 7% (n = 1) attended the in-service on the guideline.<br>**Description of the Intervention:** Participants received a copy of the guideline and an in-service explaining the guideline's recommendation.
**Data Collection**	**Method:** Chart audit.<br>**Type of Measurement:** Subjective.<br>**Description:** A Braden score was calculated for each nurse participant (dividing the number of correct Braden admission scores completed by the number of opportunities). A plan/implementation score was calculated for each nurse participant by dividing the number of assigned patients with plans/interventions performed by the number of patients under the study nurses care with an at-risk score or Stage 1 pressure ulcer requiring an intervention.<br>**Interpretation of Direction:** Higher score indicated greater behaviour.<br>**Reliability Details:** Not reported.<br>**Validity Details:** Not reported.
**Outcomes**	**Behaviour:** 1) performance of admission Braden Scores; 2) identification of a plan of care and implementation of interventions for at-risk patients.

Study	**Authors**: Linde BJ [44]<br>**Date**: 1989<br>**Country:** United States
**Objective**	To examine the effects of three different levels of communicating a practice innovation to nurses on surgical sites in two different clinical settings and to determine the rate at which nurses adopted the innovation based on the communication.
**Theoretical Framework**	Problem Solving Model (Lippitt, Watson, Wesley, 1958); Linkage model (Havelock, 1969); Diffusion Model (Rogers & Shoemaker 1971, Rogers, 1983)
**Methods**	**Design**: Cluster RCT.<br>**Sampling:** Convenience.<br>**Recruitment Methods:** Two hospitals agreed to participate. Three general surgical units at each of the hospitals chose to participate after the investigator met with the head nurses to outline the project. Units were randomly assigned by site to Levels I through III by drawing envelopes containing the number for each level of the intervention. This resulted in two units receiving each level. Nurses on the units were recruited by the principal investigator.<br>**Unit of Allocation:** Unit<br>**Unit of Analysis:** Individual (nurses)<br>**Differences in Baseline Characteristics:** Reported, non-significant.<br>**Intention to Treat Analysis:** Not applicable (groups of clinicians or patients).
**Participants**	**Size of Eligible Population:** Not reported.<br>**Sample Size**<br>**Total:** N = 185 nurses (148 at follow-up).<br>**Intervention Group #1:** n = 61 nurses (at baseline).<br>**Intervention Group #2:** n = 70 nurses (at baseline).<br>**Intervention Group #3:** n = 54 nurses (at baseline).<br>**Hospital Characteristics:** Two large Midwestern hospitals.<br>**Description of Nurse Participants:** Note: At baseline.<br>**RN:** Intervention #1 (Level III): 85.2%; Intervention #2 (Level II): 92.9%; Intervention #3 (Level 1): 92.6%.<br>**LPN:** Intervention #1 (Level III):14.8%; Intervention #2 (Level II): 7.1%; Intervention #3 (Level 1): 7.4%.<br>**Age in years, mean :** Intervention #1 (Level III): 35; Intervention #2 (Level II): 32; Intervention #3 (Level 1): 32.<br>**Years of Experience, mean:** Intervention #1 (Level III): 7.87; Intervention #2 (Level II): 7.47; Intervention #3 (Level 1): 7.68.
**Intervention Group #1**	**Type of Intervention:** Multifaceted: 1) Educational materials (printed); 2) Educational meetings (in-person); 3) Mass media; 4) Head nurse commitment and ongoing support.<br>**Evidence Based for Intervention:** Previous study published as a thesis by Linde and Biven (1971).<br>**Description of the Recipients:** Nurses on units randomized to receive the Level III intervention.<br>**Description of Deliverer:** Principal investigator introduced study and distributed protocol and head nurses providing ongoing support. Details of in-

	service not provided.<br>**Length/Duration:** One staff meeting to introduce protocol followed by use of the protocol and head nurse commitment and support for one month; In-service lasted approximately 1-hour.<br>**Adherence/Fidelity:** Not reported.<br>**Description of the Intervention:** Level III intervention (administrative support plus in-service session plus written material) to implement protocol stating how patients should be medicated patients if nurses decide to adopt the innovation, including verbal and written directions about how to document coughing-deep breathing regimen. Nurses introduced to the study by the principal investigator at a staff meeting and received a copy of the protocol which outlined the process of implementation of the intervention. In-service sessions on the practice innovation elaborated on the findings of the research-based practice innovation and answered any questions about the practice innovation. In-services were arranged individually on each nursing unit and a total of 49 in-services were held at the two sites (27 at one site and 22 at the other site). Head nurses made a commitment that their staff nurses would use the innovation for one month and that they (the head nurses) would continue to be supportive of the research project on an ongoing basis. Several bright colored signs placed on the units to remind nurses that they were participating in the project.
**Intervention Group #2**	**Type of Intervention:** Multifaceted: 1) Educational materials (printed); 2) Educational meetings (in-person); 3) Mass media.<br>**Evidence Based for Intervention:** Previous study published as a thesis by Linde and Biven (1971).<br>**Description of the Recipients:** Nurses on units randomized to receive the Level II intervention.<br>**Description of Deliverer:** Principal investigator introduced study and distributed protocol. Details of in-service not provided.<br>**Length/Duration:** One staff meeting to introduce protocol followed by use of the protocol for one month; In-service lasted approximately 1-hour.<br>**Adherence/Fidelity:** Not reported.<br>**Description of the Intervention:** Level II intervention (in-service session plus written material) to implement protocol stating how patients should be medicated patients if nurses decide to adopt the innovation, including verbal and written directions about how to document coughing-deep breathing regimen. Nurses introduced to the study by the principal investigator at a staff meeting and received a copy of the protocol which outlined the process of implementation of the intervention. In-service sessions on the practice innovation elaborated on the findings of the research-based practice innovation and answered any questions about the practice innovation. In-services were arranged individually on each nursing unit and a total of 49 in-services were held at the two sites (27 at one site and 22 at the other site). Several bright colored signs placed on the units to remind nurses that they were participating in the project.
**Intervention Group #3**	**Description of the Control:** Multifaceted: 1) Educational materials (printed); 2) Educational meeting (in-person); 3) Mass media.

	**Description of the Recipients:** Nurses on units randomized to receive the Level I intervention.<br>**Description of Deliverer:** Principal investigator introduced study and distributed protocol.<br>**Length/Duration:** One staff meeting to introduce protocol followed by use of the protocol for one month**.**<br>**Adherence/Fidelity:** Not reported.<br>**Description of the Intervention:** Level I intervention to implement protocol stating how patients should be medicated patients if nurses decide to adopt the innovation, including verbal and written directions about how to document coughing-deep breathing regimen. Nurses introduced to the study by the principal investigator at a staff meeting and received a copy of the protocol which outlined the process of implementation of the intervention. Several bright colored signs placed on the units to remind nurses that they were participating in the project.
**Data Collection**	**Name:** Questions about current nursing practice<br>**Type of Measurement:** Self-report.<br>**Description:** Two items (# 47 and 48) were developed for the study to examine nurses' current practice and rationale for medicating patients in relation to coughing and deep breathing post-operatively .<br>**Interpretation of Direction:** Higher score indicated greater behaviour.<br>**Reliability Details:** Not reported.<br>**Validity Details:** Pre-tested with 50 nurses who did not participate in the final study. Researchers stated that this determined that the questions were clearly worded and reflected how nurses medicate post-operative patients in relation to coughing and deep breathing.
**Outcomes**	**Behaviour:** Current nursing practice and rationale for medicating patients in relation to coughing and deep breathing post-operatively .
**Additional Comments:**	All participating nurses were told they could use their participation in the project to partially fulfill their responsibility for research built into career ladder programs at each site.

Study	**Authors**: Manias E, Gibson S, Finch S [48]<br>**Date**: 2011<br>**Country**: Australia
**Objective**	To examine the effectiveness of a structured educational nursing intervention on pain assessment and management in older hospitalized people.
**Theoretical Framework**	Not reported.
**Methods**	**Design**: Cluster non-randomized trial.<br>**Sampling:** Convenience.<br>**Recruitment Methods:** Recruitment was sought from nurses employed in the GEM units of the two hospitals. Nurse participants considered for inclusion were licensed nurses who had completed a 3-year bachelor's degree, and who were employed permanently by the participating hospitals. Patients were eligible to participate if they were admitted into a GEM unit, if they experienced acute or chronic pain in the past 24 hours prior to recruitment, were able to consent to participate (as demonstrated by a Mini-Mental State Examination score of at least 24 out of 30), and were under the care of a participating nurse (Figure 1).<br>**Unit of Allocation:** Hospital.<br>**Unit of Analysis:** Individual (Nurses; Patients).<br>**Differences in Baseline Characteristics:** Not reported..<br>**Intention to Treat Analysis:** Not applicable (no missing data).
**Participants**	**Size of Eligible Population:** 44 nurses.<br>**Sample Size**<br>**Total: N =** 34 nurses; N = 96 patients.<br>**Intervention Group**: Nurses: n = 17; Patients: Pre = n = 32, Post n = 32; 3 month follow-up n = 32.<br>**Control Group:** Nurses: 17; Patients: Pre = n = 32, Post n = 32; 3 month follow-up n = 32.<br>**Hospital Characteristics:** Metropolitan teaching hospital.<br>**Description of Nurse Participants:**<br>**"Nurses":** n = 34.<br>**Age in years, mean (SD):** Intervention: 47.9 (10.2), range 29-60; Control: 43.1 (12.6), range 22-60.<br>**Years of Experience, mean (SD):** Intervention: 20.7 (11.2); Control: 17.4 (13.7).
**Intervention Group**	**Type of Intervention:** Multifaceted: 1) Educational meetings (in-person, didactic, interactive with clinical demonstration); 2) Educational materials.<br>**Evidence Based for Intervention:** Evidence based guidelines for assessing and managing pain in older adults (American Geriatrics Society, 2002; Australian National Health & Medical research Council, 2005; Bucknell et al., 2001).<br>**Description of the Recipients:** Primarily targeted at nurses in the intervention hospital. Other health care professionals (two physicians, two medical residents, and one pharmacist) also attended educational meetings and clinical demonstrations. Patients cared for by nurses who received the intervention.<br>**Description of Deliverer:** A nurse educator who had expert knowledge and

	experience in pain assessment and management carried out the intervention. Nurses who in the intervention hospital delivering care to patients.<br>**Length/Duration:** 8 hour session completed within an 8-week period.<br>**Adherence/Fidelity:** Not reported.<br>**Description of the Content Provided:** Structured intervention consisting of 6 hours of instruction and 2 hours of clinical demonstration on the assessment and management of pain in older people.<br>**Description of the Intervention:** The Educational meeting (instruction) was interactive and used a case study approach with small groups two to three people to cover the following pain assessment areas: overcoming barriers to effective assessment and management, taking a patient pain history and physical examination, assessing characteristics of pain, examining assessment challenges for patients with specific needs, including those of different cultural or language backgrounds, and assessment and reassessment of pain using pain assessment tools. Nurses were instructed on the use of eight different tools for assessing The pain in older people. The educational meeting also covered principles of pain management including: selecting the most appropriate approach to pain management, which could involve pharmacological and non-pharmacological pain strategies, selecting an appropriate analgesic, establishing a management plan, selecting an appropriate route of administration, titrating the dose, optimizing relief by around-the-clock administration, and treating adverse effects. Clinical demonstration involved the nurse educator working with participating nurses to practice learned activities and to identify and resolve difficulties and barriers that may occur in actual situations with the use of real patients at the bedside. Each of the pain assessment tools introduced were made available to nurses participating in the intervention unit. Nurses were encouraged to use scales regularly on patients and were able to choose whatever scale they felt was best for the patients in their care.
**Control Group**	**Description of the Control:** Nurses in the control group received their usual staff development sessions, which were also available to nurses located in the intervention group.<br>**Description of the Recipients:** Nurses in the control hospital and the patients who they cared for.<br>**Description of Deliverer:** The nurse educator who conducted the intervention at the intervention hospital. Nurses in the control hospital delivering care to patients.<br>**Length/Duration:** The nurse educator visited the control ward during the day for an equivalent amount of time as that spent in the intervention ward over 2 weeks.<br>**Adherence/Fidelity:** Not reported.<br>**Description of the Intervention:** These staff development activities delivered in the control hospital comprised hospital in-service education sessions that were normally provided to staff on a regular basis. Staff development sessions covered diverse topics such as care of patients with dementia, language difficulties, and sensory problems and management of patients with pressure areas, depression, chronic obstructive pulmonary disease, and treatment of

	patients following a stroke.<br>**Additional Description of the Intervention:** During the study, there were no staff development sessions delivered specifically in the area of pain assessment and management in either group. The nurse educator visited the control ward during the day for an equivalent amount of time as that spent in the intervention ward. During this time, the nurse educator spoke to nurses in general terms about their care, and no specific instruction was provided on pain assessment and management. In visiting the control ward, the nurse educator did not deliver any formalized instruction in pain to ensure that time availability was not a factor that influenced the outcomes of the delivered intervention.
**Data Collection**	**Method:** Observation.<br>**Type of Measurement:** Subjective.<br>**Description:** Nurses were observed during a 2-hour observation period to determine if they used a pain assessment tool on the patients recruited in the study.<br>**Interpretation of Direction:** Higher score indicated greater behaviour (assumed).<br>**Reliability Details:** Percentage agreement on the use of the structured observation checklist between the research assistant and the authors was 100%.<br>**Validity Details:** Not reported.
	**Method:** Observation.<br>**Type of Measurement:** Subjective.<br>**Description:** Nurses' actual conduct of non-pharmacological activities were recorded during observations of nurses. Three analgesics that were targeted for consideration were morphine, oxycodone, and acetaminophen, as these were the ones most commonly used. Also of interest was the extent of prescribing of non-steroidal anti- inflammatory drugs (NSAIDs) since due to their adverse effects, other analgesics (e.g., acetaminophen) should be ordered as a first line measure.<br>**Interpretation of Direction:** Higher score indicated greater behaviour (assumed).<br>**Reliability Details:** Not reported.<br>**Validity Details:** Not reported.
	**Method:** Visual Analog Scale (VAS).<br>**Type of Measurement:** Self-report.<br>**Description:** The VAS uses a 10 cm line. The right hand point of the line represents "no pain", which is allocated a score of 0 cm, and the left hand anchor point represents "worst possible pain", which is allocated a score of 10 cm.<br>**Interpretation of Direction:** 0 cm rrepresents "no pain" and 10cm represents the "worst possible pain". Higher the score, the more pain. , which is allocated a score of 10 cm.<br>**Reliability Details:** The VAS has reported high internal consistency (Cronbach's alpha = 0.87–0.88). Presumably in this study, the intra-class correlation coefficient (ICC) results for test–retest reliability were 0.923 (95% confidence interval [CI] 0.898–0.942, $P < 0.001$) at

	rest and 0.912 (95% CI 0.883–0.934, $P < 0.001$) on movement.<br>**Validity Details:** Not reported.
**Outcomes**	**Behaviour:** Nurses' uptake of pain assessment tools; nurses management of pain using non-pharmacological measures<br>**Client:** Pain intensity.

**Study**	**Authors**: Melnyk B, Bullock T, McGrath J, Jacobson D, Kelly S, Baba L [37]<br>**Date**: 2010<br>**Country**: United States
**Objective**	To determine (a) the impact of translating the evidence-based COPE program into clinical practice on nurses' EBP beliefs and implementation, (b) the best strategy for disseminating COPE into the NICU of a children's hospital, including evaluating the use of a COPE EBP mentor (i.e., a nurse assigned specifically to work with and assist the NICU nurses at point of care in delivering COPE to parents of prematurely born infants), and (c) barriers and facilitators to successful implementation of the COPE program.
**Theoretical Framework**	Self-regulation theory (Johnson) and Control theory (Carver)
**Methods**	**Design**: Non-randomized controlled trial.<br>**Sampling:** Convenience.<br>**Recruitment Methods:** All nurses who worked either full- or part-time in the NICU of one hospital were invited to participate in the study after they were informed of the project's aims.<br>**Unit of Allocation:** Unit<br>**Unit of Analysis:** Individual (nurses)<br>**Differences in Baseline Characteristics:** Reported, Non-Significant.<br>**Intention to Treat Analysis:** Not Applicable (groups of clinicians or patients).
**Participants**	**Size of Eligible Population:** 180 nurses.<br>**Sample Size**<br>**Total:** N = 83<br>**Intervention Group**: n = 48<br>**Control Group:** n = 3<br>**Hospital Characteristics:** 55-bed NICU in a large children's hospital in the southwest region of the United States.<br>**Description of Nurse Participants:**<br>**"Nurses":** n = 81..<br>**Age in years, mean (SD):** Intervention: 41.6 (10); Control: 35 (10); Note: Overall range from 23-69 years.<br>**Years of Experience, mean (SD):** Intervention: 13.5 (8.4); Control: 9.6 (10.3).
**Intervention Group**	**Type of Intervention:** Multifaceted: 1) Educational materials (electronic) or Educational meetings and 2) EBP mentor.<br>**Evidence Based for Intervention:** Not applicable.<br>**Description of the Recipients:** Nurses who worked either full- or part-time in 2 pods of the 55-bed NICU who decided to voluntarily participated in the study.<br>**Description of Deliverer:** Not reported.<br>**Length/Duration:** Not reported. The authors did not indicate for how long workshops were offered for, nor now long COPE EBP mentor was available to the nurses. The authors did indicate that 3 months following the implementation of the COPE program, the COPE EBP mentor was introduced and that follow-

	up data was collected at 6 months.<br>**Adherence/Fidelity:** 95% of nurses who worked in the 2 pods attended the workshop. Nurses unable to attend the workshop viewed the taped workshop on a DVD so that all nurses who worked in these pods received the information.<br>**Description of the Intervention:** An 8 hour workshop on evidence based practice (EBP) and the Creating Opportunities for Parent Empowerment (COPE) program. Content of the workshop included (a) a description of EBP and the steps of implementation (i.e. ask the PICO question, patient population, intervention or issue of interest, comparison intervention, outcome), search for the best evidence, critically appraise the evidence, integrate the best evidence with clinical experience and patient references/value, and evaluate the outcome of the evidence based practice change; (b) barriers to and facilitators of EBP; (c) a description of the COPE program; (d) findings from the COPE pilot study and full-scale clinical trial; and (e) details on how to administer the COPE program. Three months following the workshop, a COPE EBP mentor was introduced as a strategy to enhance implementation of the COPE program. The mentor, who functioned in the role for a few hours twice a week, also made sure that the materials to pro- vide COPE were available in a way that was supportive to the staff. She also listened to the staff members and was empathetic to their difficulties, offering suggestions and support when competing priorities made implementation of the program challenging.
**Control Group**	**Description of the Control:** No intervention.<br>**Description of the Recipients**: Nurses who worked either full- or part-time in 3 pods of the 55-bed NICU who decided to voluntarily participated in the study.
**Data Collection**	**Method:** Evidence-Based Practice Implementation (EBPI) scale.<br>**Type of Measurement:** Self report.<br>**Description:** The actual conduct of EBP. It is an 18 item, 5-point Likert scale about whether specific EBPs have been performed in the previous 8 weeks.<br>**Interpretation of Direction:** Subjects respond to each question by answering 0 (not at all) to 4 (very often). Item scores are summed with a range of scores from 0 to 60. Higher scores indicate greater behaviour.<br>**Reliability Details:** Cronbach α values have consistently been reported as 0.85 and higher. Internal consistency reliability with this sample averaged 0.95.<br>**Validity Details:** Content validity and clarity of this scale was established by experts in evidence based practice. Construct validity was recently supported through factor analysis.
**Outcomes**	**Behaviour:** Implementation of evidence-based practices.

**Study**	**Authors**: Middleton S, McElduff P, Grimshaw J, Dale S, Griffiths R, Cheung NW, et al. [36] ; Middleton S, Levi C, Ward J, Grimshaw J, Griffiths R, D'Este C [64-65]<br>**Date**: 2011<br>**Country:** Australia
**Objective**	To assess patient outcomes 90 days after hospital admission for stroke following a multidisciplinary intervention targeting evidence-based management of fever, hyerglycaemia, and swallowing dysfunction in acute stroke units (ASUs).
**Theoretical Framework**	None reported.
**Methods**	**Design**: Cluster RCT<br>**Sampling:** Nineteen ASUs agreed to participate.<br>**Recruitment Methods:** Eligible ASUs were those located in large, tertiary referral centres which provided care for stroke patients in a geographically defined location with immediate CT access and on-site high-dependency units. Both units with (Category A) and without (Category B) access to on-site neurosurgery were eligible.<br>**Unit of Allocation:** Unit<br>**Unit of Analysis:** Individual (patient)<br>**Differences in Baseline Characteristics:** Reported, non-significant.<br>**Intention to Treat Analysis:** Not applicable (groups of clinicians or patients).
**Participants**	**Size of Eligible Population:** 20 ASUs<br>**Sample Size**<br>**Total:** 19 hospitals, N = 340 medical records.<br>**Intervention Group:** n = 10 (units); n = 500 patients (follow-up).<br>**Intervention Group:** n = 9 units; n = 626 patients (follow-up).<br>**Hospital Characteristics:** Large, tertiary referral centres in New South Wales, Australia.<br>**Description of Nurse Participants:** Not reported
**Intervention Group**	**Type of Intervention:** Multifaceted: 1) Educational meetings (assumed in-person); 2) Educational outreach (interactive and didactic); 3) Reminders (site visits, telephone support; email supports)<br>**Evidence Based for Intervention:** National Stroke Foundation (Australia): Clinical guidelines for stroke.<br>**Description of the Recipients:** Reported as “all ASU clinicians” in 10 ASUs.<br>**Description of Deliverer:** Panel of experts developed clinical treatment protocols from the guideline. Overall the intervention was implemented with multidisciplinary support from physicians, speech pathologists and nurses. Clinical protocols were delivered by bedside nurses.<br>**Length/Duration:** May 15, 2007 to August 25, 2010.<br>**Adherence/Fidelity:** Not reported.<br>**Description of the Intervention:** Intervention focused on barrier identification, reinforcement of multidisciplinary teamwork, local adaptation, and use of site

	champions. From the guidelines, a panel of experts developed clinical treatment protocols for management of fever, hyperglycaemia, and swallowing for the first 72 hours after ASU admission. The intervention aimed to prompt nursing assessment and bedside treatment. Two team-building workshops were held to identify local barriers to multidisciplinary care and enablers to implementation of the nurse-initiated treatment protocols. Two site-based educational outreach meetings(interactive and didactic) were held for clinicians to discuss the protocols. Ongoing activities included site visits, telephone, and email support as reminders.
**Control Group**	**Type of Intervention:** Educational Materials.<br>**Evidence Based for Intervention:** National Stroke Foundation (Australia): Clinical guidelines for stroke.<br>**Description of the Recipients:** Clinicians in 9 ASUs.<br>**Description of Deliverer:** Not reported.<br>**Length/Duration:** Intervention implemented from May 15, 2007 to August 25, 2010. Not reported when educational materials were distributed.<br>**Adherence/Fidelity:** Not reported.<br>**Description of the Intervention:** ASUs in the control group received only an abridged version of existing guidelines.
**Data Collection**	**Data Collection:** Chart audit<br>**Type of Measurement:** Subjective<br>**Description:** Temperature<br>**Interpretation of Direction:** Greater ºC, indicates higher temperature.<br>**Reliability Details:** Of the first 700 audits conducted by two trained auditors, 10% were re-audited with agreement occurring 95% of the time.<br>**Validity Details:** Not reported.
	**Data Collection:** Chart audit<br>**Type of Measurement:** Subjective<br>**Description:** Blood glucose<br>**Interpretation of Direction:** Greater blood glucose (mmol/L), indicates higher blood glucose.<br>**Reliability Details:** Of the first 700 audits conducted by two trained auditors, 10% were re-audited with agreement occurring 95% of the time.<br>**Validity Details:** Not reported.
	**Data Collection:** Chart audit<br>**Type of Measurement:** Subjective<br>**Description:** Discharge diagnosis of aspiration pneumonia.<br>**Interpretation of Direction:** Increased percentage indicates greater diagnoses with aspiration pneumonia.<br>**Reliability Details:** Of the first 700 audits conducted by two trained auditors, 10% were re-audited with agreement occurring 95% of the time.<br>**Validity Details:** Not reported.
	**Data Collection:** Chart audit<br>**Type of Measurement:** Subjective<br>**Description:** Swallowing screening done within 24 hours of ASU admission.<br>**Interpretation of Direction:** Increased percentage indicates more screenings

	completed.<br>**Reliability Details:** Of the first 700 audits conducted by two trained auditors, 10% were re-audited with agreement occurring 95% of the time.<br>**Validity Details:** Not reported.
	**Data Collection:** Chart audit (assumed)<br>**Type of Measurement:** Subjective<br>**Description:** Length of stay.<br>**Interpretation of Direction:** Greater days, indicate greater length of stay (assumed).<br>**Reliability Details:** Not reported.<br>**Validity Details:** Not reported.
	**Data Collection:** Computer assisted telephone interviews<br>**Type of Measurement:** Subjective<br>**Description:** Death or dependency was measured using the modified Rankin Score (mRS); one question.<br>**Interpretation of Direction:** This is a 7-point scale measuring which ranges from 0 to 6; where 0 equals no symptoms, 5 equals severe disability and 6 equals death. Disability was defined as an mRS of ≥ 2.<br>**Reliability Details:** Two reviewers underwent online training and competency for administration of the mRS.<br>**Validity Details:** Not reported.
	**Data Collection:** Computer assisted telephone interviews<br>**Type of Measurement:** Subjective<br>**Description:** Health status was measured using the SF-36 that includes a single 'health transition rating' and scores eight health domains, aggregated to form the Physical Component Summary (PCS) score and the Mental Component Summary (MCS) score.<br>**Interpretation of Direction:** Higher mean summary scores reflect better states of health and well-being.<br>**Reliability Details:** Not reported.<br>**Validity Details:** Not reported.
**Outcomes**	**Client:** 1) Death or dependency, 2) Functional dependence, 3) Health status, 4) Temperature, 5) Blood glucose, 6) Discharge diagnosis of aspiration pneumonia, 8) Length of hospital stay

**Study**	**Authors**: Ploeg J, Davies B, Edwards N, Gifford W, Miller PE [52]; Edwards N, Davies B, Ploeg J, Dobbins M, Skelly J, Griffin P, et al. [67]<br>**Date**: 2007<br>**Country**: Canada
**Objective**	The purpose of this study was to describe and compare the perceptions and experiences of staff, health care administrators, and clinical resource nurses (CRNs) or project leaders regarding factors that influenced implementation of nursing best-practice guideline recommendations.
**Theoretical Framework**	Not reported
**Methods**	**Design**: Qualitative (Descriptive).<br>**Sampling:** Criterion sampling.<br>**Recruitment Methods:** Participants were recruited from the 22 agencies across Ontario, Canada implementing Registered Nurses Association of Ontario (RNAO) Best Practice Guidelines (BPGs). How these agencies were recruited is not reported. Criterion sampling for participants in this study was used and included: (1) the CRN for each guideline implementation, (2) health care providers from each participating unit, and (3) administrators or nursing managers at each participating unit/agency. CRNs were asked to identify staff who had favorable experiences as well as those who might have had less favorable experiences with the implementation process. CRNs also identified approximately 5–10 administrators from each organization either at the unit or organizational level who were directly or indirectly involved. Names of potential participants were forwarded to the research associate, who contacted them and invited them to participate in a telephone interview.
**Participants**	**Size of Eligible Population:** 22 organizations in Cycle 2 of the implementation.<br>**Sample Size:** 125 individual interviews.<br>**Hospital Characteristics:** Organizations (acute, long-term care agencies, and community care organizations) in Ontario, Canada ranging in size from approx. 150-750 beds. In hospitals, guidelines were implemented on diverse units including postpartum, medical, surgical, intensive care, neurology, orthopedic, mental health, complex continuing care, oncology, and palliative care.<br>**Description of Nurse Participants:**<br>**Staff:** n = 58.<br>**CRNs:** n = 8.<br>**Administrators:** n = 59.<br>**Age:** Not reported.<br>**Years of Experience:** Not reported.
**Intervention**	**Type of Intervention:** Multifaceted: 1) Educational materials (printed); 2) Educational meetings, 3) Knowledge broker (Clinical Resource Nurse), 4) Other (partial funding for knowledge broker for six to nine months).<br>**Evidence Based for Intervention:** RNAO BPGs (Client Centered Care, Crisis Intervention, Healthy Adolescent Development, Pain Assessment, Pressure Ulcers, Supporting and Strengthening Families, Therapeutic Relationships).<br>**Description of the Recipients:** Assume staff nurses, clinical resource nurses administrators, professional license, and RN/RPNs.

	**Description of Deliverer:** Clinical resource nurses, nursing managers, and administrators.<br>**Length/Duration:** Second cycle of implementation.<br>**Adherence/Fidelity:** Not reported.<br>**Description of the Intervention:** Guidelines were implemented as part of the RNAO BPG project, a multi-year initiative to develop and evaluate nursing clinical guidelines in the province of Ontario, Canada. Participating organizations were provided with partial funding for six to nine months for a CRN to lead implementation, a formal launch organized by the RNAO, an orientation workshop for managers, senior administrators, and CRNs that focused on implementation strategies and evaluation processes, and pre/post evaluations for each BPG. CRNs worked with key stakeholders at the organizations to develop implementation strategies. Monthly teleconferences with the CRNs, a member of each of the BPG development panels, and the program manager from RNAO to share strategies for BPG implementation.
**Data Collection**	**Method:** Semi-structured telephone interviews.<br>**Additional Details:** Participants were asked about their perceptions of the guideline, the facilitators and barriers influencing implementation, changes attributed to implementing the guideline and their experiences with the implementation process.
**Outcomes**	**Contextual Factors**

**Study**	**Authors**: Royle JA, Blythe C, Ingram A, DiCenso A, Bhatnager N, Potvin C [57]<br>**Date**: 1996<br>**Country:** Canada
**Objective**	The objectives of this study were to 1) enhance research utilization in a selected setting by introducing a framework for research-based care, and 2) evaluate the outcomes of research utilization on a specific clinical nursing problem chosen by nurses and researchers.
**Theoretical Framework**	Not reported.
**Methods**	**Design**: Qualitative (Descriptive)<br>**Sampling:** Convenience<br>**Recruitment Methods:** The recruitment/selection of the single teaching hospital was not reported. How nurses recruited to participate in this study was not reported.
**Participants**	**Size of Eligible Population:** Not reported<br>**Sample Size:** 22 nurses<br>**Hospital Characteristics:** 12-bed haematology/bone marrow transplant unit in a teaching hospital.<br>**Description of Nurse Participants:**<br>**"Nurses":** n = 22<br>**Age in years (mean):** Not reported.<br>**Years of Experience (mean):** 14.48.
**Intervention #1**	**Type of Intervention:** Multifaceted: 1) Educational meetings, 2) Training and support, 3) Support from managers.<br>**Evidence Based for Intervention:** Review of the literature on research utilization and action research.<br>**Description of the Recipients:** Nurses on the unit.<br>**Description of Deliverer:** Not reported.<br>**Length/Duration:** Not reported.<br>**Adherence/Fidelity:** Not reported.<br>**Description of the Intervention:** Nominal Group Technique used to help nurses identify, prioritize, and select a clinical problem. The nurses had regular meetings with the project team to review and critique the literature relevant to the clinical problem, set goals, select an intervention, and choose pre- and post-intervention measures. Meetings and ongoing communication with interested nursing managers and members of other health professions provided cooperation and support for the planned change in nursing practice.
**Data Collection**	**Method:** Focus group interviews.<br>**Additional Description:** Focus groups were conducted to evaluate the action research techniques and gather qualitative data on the outcome of the intervention.
**Outcomes**	**Contextual Factors**

**Study**	**Authors**: Seers K, Crichton N, Carroll D, Richards S, Saunders T [41]<br>**Date**: 2004<br>**Country:** United Kingdom
**Objective**	To assess whether implementing evidence-based pain management improved postoperative pain outcomes.
**Theoretical Framework**	Not reported
**Methods**	**Design**: Cluster RCT.<br>**Sampling:** Convenience.<br>**Recruitment Methods:** The sisters and staff on the surgical wards involved in the baseline audit and subsequent study were approached and gave consent for the study to occur. Patients from the surgical wards gave permission for data to be extracted from their notes.<br>**Unit of Allocation:** Unit<br>**Unit of Analysis:** Individual (patients).<br>**Differences in Baseline Characteristics:** Reported, significant.<br>**Intention to Treat Analysis:** Not applicable (groups of clinicians or patients).
**Participants**	**Size of Eligible Population:** Not reported.<br>**Sample Size**<br>**Total:** N = 120.<br>**Intervention Group**: patients on 2 surgical wards; n =58 patients at baseline, n = 60 patients at follow-up.<br>**Control Group:** patients on 2 surgical wards; n = 52 patients at baseline, n = 60 patients at follow-up .<br>**Hospital Characteristics:** 130 bed specialist orthopaedic NHS Trust hospital in South England with a mixture of medical and surgical patients, teaching hospital.<br>**Description of Nurse Participants:**<br>**Professional designation:** Not reported.<br>**Age:** Not reported.<br>**Years of Experience:** Not reported.
**Intervention Group**	**Type of Intervention:** Multifaceted:1) Audit & feedback, 2) Educational material (printed), and 3) Educational meeting (interactive, in-person).<br>**Evidence Based for Intervention:** Evidence from a series of systematic reviews were compared to produce a league table for oral postoperative analgesics (p. 184).<br>**Description of the Recipients:** Not reported. Assume that nurses on 2 surgical wards were part of a multidisciplinary team that received the intervention. The authors state that "the plan was to have all the pain link nurses at these sessions and as many others as possible, and use a cascade system to pass on the information" (p. 188).<br>**Description of Deliverer:** An external expert facilitated session 4.<br>**Length/Duration:** Sessions 1 – 3 lasted approximately 2 hours and session 4 was a half-day workshop. The 2 wards received the intervention over approximately 6 weeks.<br>**Adherence/Fidelity:** Although four teaching sessions were prearranged at

	agreed times, they had to be repeated several times because key staff were off sick or the ward was too busy.<br>**Description of the Intervention:** Four interactive sessions were planned to review the following: 1) detailed feedback of baseline data and discussion (utilizing audit and feedback), 2)why systematic reviews?, analgesic league tables and choice of drugs to develop an analgesic algorithm (see Figure 1), 3) principles of EBHC, including critical appraisal, and 4) a facilitation and change workshop, included key consideration in implementing evidence and changing clinical practice; promoting an awareness of the different roles and contributions of group members during a change; developing an understanding of facilitation skills and how these support effective group work and change management; planning how to implement the algorithm. The intervention was designed to me discursive and interactive. Additional support/teaching included, for example, use of the Internet and how to search databases.
**Control**	**Description of the Control:** The control wards did not receive the intervention.<br>**Description of the Recipients**: Not reported. Assume that nurses on 2 surgical wards were part of a multidisciplinary team that did not receive the intervention.<br>**Description of Deliverer:** Not applicable.<br>**Length/Duration:** Not applicable.<br>**Adherence/Fidelity:** Not applicable.
**Data Collection**	**Method:** Interviews with patients.<br>**Type of Measurement:** Self-report.<br>**Description:** Pain was assessed at rest and on movement using a 10-point numerical rating scale.<br>**Interpretation of Direction:** On a 10-point scale, 0 is no pain and 10 is worst pain possible. Higher pain scores indicate worse pain.<br>**Reliability Details:** Not reported.<br>**Validity Details:** Not reported.
**Outcomes**	**Client Outcomes:** Pain and worst pain since surgery (at rest, on movement)

**Study**	**Authors**: Stetler CB, Caramanica L [50]<br>**Date**: 2007<br>**Country:** United States
**Objective**	In this article a brief evaluation of a service-based initiative with the aim of obtaining insights regarding multiple types and levels of EBP-related outcomes is described.
**Theoretical Framework**	Stetler and Baystate models of evidence-based practice.
**Methods**	**Design**: Qualitative (Descriptive).<br>**Sampling:** Convenience.<br>**Recruitment Methods:** Nursing leadership at one hospital engaged in activities to enhance the conduct and use of research. How participants recruited for this study is not reported.
**Participants**	**Size of Eligible Population:** Not reported.<br>**Sample Size:** 37 Nurses from 12 self-identified teams (SiTs).<br>**Hospital Characteristics:** Hartford Hospital which is a large, acute care, teaching hospital in Hartford, Connecticut, USA.<br>**Description of Nurse Participants:**<br>**Professional designation:** Mix of staff nurses, specialty nurses (e.g., advanced practice nurses, clinical nurse specialists, or educators), and a few nurse managers.<br>**Age:** Not reported.<br>**Years of Experience** Not reported.
**Intervention**	**Type of Intervention:** Multifaceted: 1) Educational Meetings (interactive) and 2) Other (SiTs).<br>**Evidence Based for Intervention:** Authors cite several evidence-based practice and research utilization literature.<br>**Description of the Recipients:** Assume nurses who participated in components of the RRP and SiTs.<br>**Description of Deliverer:** Support in the form of one or two facilitators. Not indicated who conducted educational sessions.<br>**Length/Duration:** 1999-2005. It is not reported how long each SiT was supported or operationalized.<br>**Adherence/Fidelity:** > 1,200 attendees at educational sessions<br>**Description of the Intervention:** A Research Roundtable Program (RRP) existed from 1999-2005. The RRP focused on four formal goals: conduct of outcomes research, development of an action plan to incorporate/translate new knowledge gained into the practice setting, promotion of a spirit of inquiry/an evidence-based approach to practice, and achievement of formal or measurable success in terms of improving patient outcomes, addressing practice question(s)/current clinical and practical needs, and developing nurses' research utilization skills. The RRP consisted of the following operational components: series of four monthly educational sessions covering a standard set of roundtable topics (i.e., formulating<br>a search strategy, critiquing research (2 sessions) and transitioning to the next appropriate steps per evidence from critiqued studies, a self-identified team

	(SiT) from a clinical unit or specialty service that was focused on defining a study issue, doing a literature review, and conducting an outcomes research study and/or applying findings to practice, support in the form of one or two facilitators which were targeted to but not limited to the SiTs. From 1995-2005, 12 roundtables and related SiTs were created. Involvement in the SiTs began with team members attending and focusing a particular educational series on their identified issue.
**Data Collection**	**Method:** Semi-structured focus group interviews and use of available documents.<br>**Additional Details:** Opening interview questions were designed to clarify the nature of each SiT roundtable with contextual and end result questions focused on clarifying: purpose of each roundtable (including its identified practice question(s)/current clinical and practical needs), the project's change needs and related plans for implementation, the team's historical or current trajectory, and the nature of the roundtable's outcomes and related evaluation methods. The interview also asked participants about their individual research utilization outcomes and those of their colleagues. Participants also completed individual checklists focused on participants' potential use of research evidence, the spirit-of-inquiry aim, clinical scholarship, and use of the research evidence by colleagues not on the SiTs.
**Outcomes**	**Contextual factors**

**Study**	**Authors**: Sulch D, Perez I, Melbourn A, Kalra L [47]<br>**Date**: 2000<br>**Country:** United Kingdom
**Objective**	To evaluate the effectiveness of integrated care pathway (ICP) based management in reducing the length of hospital stay without affecting functional outcome in stroke patients undergoing specialist rehabilitation.
**Theoretical Framework**	No
**Methods**	**Design**: RCT.<br>**Sampling:** Convenience.<br>**Recruitment Methods:** The sample was drawn from 335 consecutive, acute-stroke patients. Patients were eligible if they had persistent motor, sensory, vision, speech, perceptual, or cognitive impairment resulting from a limitation of personal activities for daily living and required inpatient rehabilitation. One hundred fifty-two eligible patients were randomized.<br>**Unit of Allocation:** Individuals (patients) to the intervention or control bed area.<br>**Unit of Analysis:** Individual (patients)<br>**Differences in Baseline Characteristics:** Reported, non-significant.<br>**Intention to Treat Analysis:** Yes.
**Participants**	**Size of Eligible Population:** 335 patients<br>**Sample Size**<br>**Total:** N = 152 patients<br>**Intervention Group:** n = 76 patients<br>**Control Group:** n = 76 patients<br>**Hospital Characteristics:** Not reported<br>**Description of Nurse Participants:** Not reported.
**Intervention Group**	**Type of Intervention:** Educational Meeting.<br>**Evidence Based for Intervention:** The integrated care pathway (ICP) was developed through an extensive review of the available literature w/ MEDLINE, CINAHL, Nursing and Health services databases. Information was also collected on ICP projects that were not published but were known to members of the multidisciplinary team. This information was collated with local data and experience to be relevant to local service requirements. Each professional group listed therapeutic activities necessary for ensuring best practice in rehabilitation and discharge planning.<br>**Description of the Recipients:** Patients cared for by a multidisciplinary team (a physician, nurses, physiotherapists, occupational therapists, speech therapists, and a social worker) who developed the ICP and received multidisciplinary training sessions on the ICP.<br>**Description of Deliverer:** A senior nurse with experience in acute care, rehabilitation, and management was appointed to implement the ICP. Members of the multidisciplinary team who received training sessions on the ICP.<br>**Length/Duration:** The ICP was piloted for a 3-month period.<br>**Adherence/Fidelity:** Review of the ICP records in 76 patients in the intervention group showed good compliance with the care pathway in all

	domains assessed. The vast majority of interventions and events were recorded appropriately; only 14 (18%) sets of records showed incomplete documentation in 1 or more domains assessed. At least 80% of the specified interventions had been undertaken in >80% of the patients who had complete records.<br>**Description of the Intervention:** The ICP was a means of defining therapeutic activities, short-term goals, and time taken to achieve goals in advance. The ICP is located in the Appendix of the article. The ICP lists the therapeutic activities necessary for ensuring best practice in rehabilitation and discharge planning. Specific activities are grouped according to stage and predicted patient needs at a given time. Key short-term goals for each therapeutic intervention and the time estimated to achieve these were defined in advance. The ICP was piloted for a 3-month period in the study area to achieve staff compliance with the new methodology, resolve operational problems, and reduce practice bias in the study. Multidisciplinary training session included the philosophy, operational aspects, and expected gains of the ICP.
**Control**	**Description of the Control:** Conventional care, provided by means of the multidisciplinary model of care.<br>**Description of the Recipients:** Patients cared for by a multidisciplinary team providing conventional care.<br>**Description of Deliverer:** Multidisciplinary team members providing conventional care.<br>**Length/Duration:** 3 months.<br>**Adherence/Fidelity:** Not reported.<br>**Description of the Intervention** Patients were assessed comprehensively, and an individualized rehabilitation program was designed by members of the multidisciplinary team. In contrast to the ICP method, in which therapeutic activities, short-term goals, and the time taken to achieve these goals were defined in advance, these aspects were discussed in weekly multidisciplinary meetings and determined on the basis of patients' progress. The multidisciplinary process of care and documentation was reviewed, and a 3-month period of strict implementation of all aspects of multidisciplinary care was undertaken to exclude bias caused by the placebo effect of undertaking the trial.
**Data Collection**	**Method:** Chart audit (assumed).<br>**Type of Measurement:** Subjective.<br>**Description:** Not provided.<br>**Interpretation of Direction:** Greater days, indicate greater length of stay (assumed).<br>**Reliability Details:** Not reported for length of stay.<br>**Validity Details:** Not reported.
**Outcomes**	**Client:** Length of stay

Study	**Authors**: Titler G, Herr K, Brooks J, Xie X, Ardery G, Schilling M, et al. [38]<br>**Date**: 2009<br>**Country:** United States
**Objective**	To test an interdisciplinary, multifaceted, translating research into practice (TRIP) intervention to (a) promote adoption, by physicians and nurses, of evidence based (EB) acute pain management practices in hospitalized older adults, (b) decrease barriers to use of EB acute pain management practices, and (c) decrease pain intensity of older hospitalized adults.
**Theoretical Framework**	A translation research model (Titler and Everett), developed from Roger's Diffusion of Innovations model.
**Methods**	**Design**: Cluster RCT.<br>**Sampling:** Convenience.<br>**Recruitment Methods:** Twelve Midwest acute care hospitals that discharged at least 30 patients ≥ 65 years of age per year with a hip fracture were stratified and randomfized. Hospitals identified the principal non-ICU units where adult hip fracture patients were admitted and the intervention was implemented on study units at the six hospitals in the intervention group. The sample consisted of the medical records of patients who met inclusion criteria and the nurses and physicians who cared for the patients.<br>**Unit of Allocation:** Hospital.<br>**Unit of Analysis:** Individual (patients).<br>**Differences in Baseline Characteristics:** Reported, non-significant.<br>**Intention to Treat Analysis:** Not reported/Not applicable (groups of clinicians or patients).
**Participants**	**Size of Eligible Population:** Not reported.<br>**Sample Size**<br>**Total:** 12 hospitals<br>**Intervention Group:** 6 hospitals; Preintervention: n = 379 patient medical records; Postintervention: n = 338 patient medical records.<br>**Control Group:** 6 hospitals; Preintervention: n = 353 patient medical records; Postintervention: n = 331 patient medical records.<br>**Hospital Characteristics:** Acute care hospitals in the Midwest that discharged at least 30 patients ≥ 65 years of age per year with a hip fracture.<br>**Description of Nurse Participants:**<br>**"Nurses":** 198.<br>**Age in years, mean (SD):** 39.3 (9.1).<br>**Years of Experience, mean:** 5.
**Intervention Group**	**Type of Intervention:** Multifaceted: 1) Educational meetings (distance, assume in-person), 2) Educational materials (printed), 3) Mass media, 4) Educational outreach visits, 5) Local opinion leader, 6) Change champion, 7) Audit & Feedback.<br>**Evidence Based for Intervention:** Evidence based practice guideline on *Acute Pain Management in the Elderly* (Herr et al., 2000).<br>**Description of the Recipients:** Nurses and physicians who cared for patients > 65 years old admitted with a hip fracture in the hospitals receiving the intervention.

	**Description of Deliverer:** Multiple sources including researchers and opinion leaders.<br>**Length/Duration:** Engagement Phase: July 2000 – December 2000; Implementation Phase: January 2001- April 2002.<br>**Adherence/Fidelity:** Not reported.<br>**Description of the Intervention:** The TRIP intervention included quick reference guides developed by the research team and reviewed by four national pain experts on pain assessment, pharmacological treatment recommendations, equianalgesic chart, non-pharmacological treatment, and patient and family education related to pain management. Each hospital assigned to the intervention received multiple copies of the guideline, laminated pain rating scales in each patient room, the quick reference guides to keep desired pain management practices visible, a computer for each unit connect to the internet with directions for accessing an acute pain management website developed by the research team, and resources texts, videotapes and training manuals. Focus groups (n = 18) with nurses, physicians, and senior leaders were used to introduce the acute pain guideline, discuss perceptions regarding importance, value, and benefits of acute pain management, and elicit feedback on the quick reference guides. A 60-minute continuing education program for senior administrative leaders to discuss their role in promoting the adoption of the guideline. Twice during implementation, chief nurse executives were provide brief articles about the project specifically written for each hospital. Early in the intervention, study investigators met with physicians and nurses at the intervention sites to review baseline performance indicators of acute pain management. Nursing and medical staff were educated via a web-based course. Audit and feedback of pain data were abstracted from patient medical records and presented to the nurses and physicians every 6 weeks for 10 months (6 reports). Monthly teleconferences (n = 11) were used among nurses working on the project to discuss issues, strategies for overcoming barriers, progress made in education of the staff, and revision of policies. Local opinion leaders (nurse and physician) and nurse change champions were also used during the intervention, as well as educational outreach visits. Nurse opinion leaders and nurse change champions who were educated via a 3-day train the trainer program and physician opinion leaders trained by researchers via 60-minute educational discussion. Nurse and physician opinion leaders led organization and unit-level system changes to support use of evidence-based acute pain management practices, led education of their peer group, altered practice norms, and influenced their peers through coaching. The nurse change champions and physician opinion leaders circulated facts on acute pain management of older adults and encourage their colleagues to align their pain practices with the evidence. Educational outreach visit occurred every 3 weeks (13 visits) by an advanced practice nurse with pain management expertise to consult with the nurses and physicians on pain management practice. The intervention concluded with a meeting of nurse opinion leaders, change champions, and managers from all the intervention sites.

**Control Group**	**Type:** Educational materials (printed).<br>**Description of the Control:** Evidence based practice guideline on *Acute Pain Management in the Elderly* (Herr et al., 2000).<br>**Description of the Recipients:** Nurses and physicians who cared for the patients > 65 years old admitted with a hip fracture in the hospital in the control hospitals.<br>**Description of Deliverer:** Not reported.<br>**Length/Duration:** Not reported.<br>**Adherence/Fidelity:** Not reported.<br>**Description of the Intervention:** Received the evidence based practice guideline on *Acute Pain Management in the Elderly* (Herr et al., 2000).
**Data Collection**	**Method:** Medical record abstract form (MRAF).<br>**Type of Measurement:** Subjective.<br>**Description:** A 19-page medical record abstract instrument was used to determine nurse and physician acute pain management practices. It was created by investigators with expertise in acute pain management.<br>**Interpretation of Direction:** The MRAF the source of data for mean pain intensity as documented on a 0 (no pain) to 10 (worst pain) scale.<br>**Reliability Details:** Inter-rater reliability (r = 0.92– 0.95) was demonstrated through abstraction of 10 records by two individuals trained in use of the instrument. Intra-rater reliability (0.92 – 1.0) was demonstrated by the trained research assistant abstracting 25 of the same records 6 months following the initial abstraction.<br>**Validity Details:** Content validity was achieved through review by three investigators with expertise in acute pain.
**Outcomes**	**Client:** Pain intensity

**Study**	**Authors**: Tranmer JE, Lochhaus-Gerlach J, Lam M [42]<br>**Date**: 2002<br>**Country:** Canada
**Objective**	To determine the effect of participation in research on staff nurses' attitude towards, access to, perceived support of and reported use of research in practice.
**Theoretical Framework**	Not reported.
**Methods**	**Design**: Cluster non-randomized trial.<br>**Sampling:** Convenience.<br>**Recruitment Methods:** Six medical surgical units were randomly assigned to receive three different levels of exposure to research: high, low and usual. One group was nurses on low and high participation units volunteered to be part of the research working groups. A second group was all staff nurses on the six medical surgical units, not directly involved in the research working groups.<br>**Unit of Allocation:** Unit.<br>**Unit of Analysis:** Unit.<br>**Differences in Baseline Characteristics:** Reported, significant<br>**Intention to Treat Analysis:** Not applicable (groups of nurses or patients).
**Participants**	**Size of Eligible Population:** Not reported.<br>**Sample Size**<br>**Total:** N = 190 nurses (92 at baseline, 88 at follow-up)<br>**Intervention Group #1 (High Participation):** n = 37 nurses (at baseline)<br>**Intervention Group #2 (Low Participation):** n = 21 nurses (at baseline)<br>**Control Group:** n = 34 nurses (at baseline)<br>**Hospital Characteristics:** 424 bed university affiliated acute care, teaching hospital in southeastern Ontario.<br>**Description of Nurse Participants:** Note: At baseline.<br>**RN:** Overall: n = 76 (83%); Intervention #1: n = 30 (81%); Intervention #2: n = 18 (86%); Control: n = 28 (82%)<br>**RPN:** At baseline, Overall: n = 8 (9%); Intervention #1: n = 3 (8%); Intervention #2: n = 2 (10%); Control: n = 3 (9%).<br>**Age, years:** Overall: 20-29 n = 20 (22%), 30-39 n = 50 (56%); 40-49 n = 15 (16%), > 50 n = 7 (8%); Intervention #1: 20-29 n 7 = (18%), 30-39 n = 20 (54%); 40-49 n = 6 (16%), > 50 n = 4 (10%); Intervention #2: 20-29 n = 2 (10%), 30-39 n = 10 (48%); 40-49 n = 7 (33%), > 50 n = 2 (10%); Control: 20-29 n = 11 (33%), 30-39 n = 20 (40%); 40-49 n = 2 (6%), > 50 n = 1 (3%).<br>**Years of Experience, mean (SD):** Overall: 8; Intervention #1: 8(5); Intervention #2: 12(5); Control: 8(5).
**Intervention Group #1**	**Type of Intervention:** Multifaceted: 1) Clinical working group, including educational meetings (20 hours of workshop/protected time; in-person); 2) Implementation of research protocol.<br>**Evidence Based for Intervention:** Not applicable.

	**Description of the Recipients:** Eighteen nurses from the high participation group participated in the clinical research working group. All other staff nurses on high participation units were not directly involved with the clinical research working group and engaged in the implementation of the clinical research protocol.<br>**Description of Deliverer:** Not reported.<br>**Length/Duration:** Not reported; assume study length 1year from baseline to follow-up data collection.<br>**Adherence/Fidelity:** Not reported.<br>**Description of the Intervention:** Participants in the clinical research working groups learned how to review and critique literature, complete a literature review on practice issue, participated in the design of study to address practice issue, and participated in implementation of the research study. All staff nurses in high participation units engaged in implementation of the clinical research protocol and had approximately 20 hours of workshop/protected time. They were responsible for assisting with patient recruitment and data collection.
**Intervention Group #2**	**Type of Intervention:** Multifaceted: 1) Clinical research working groups, including educational meetings (in-person, one 8-hour workshop); 2) Implementation of research protocol.<br>**Evidence Based for Intervention:** Not applicable.<br>**Description of the Recipients:** Ten nurses from the low participation group participated in the clinical research working group. All other staff nurses on low participation units were not directly involved with the clinical research working group and engaged in the implementation of the clinical research protocol.<br>**Description of Deliverer:** Not reported.<br>**Length/Duration:** Not reported; assume study length was 1year from baseline to follow-up data collection.<br>**Adherence/Fidelity:** Not reported.<br>**Description of the Content Provided:** Participants in the clinical research working group learned about the background literature related to the specific clinical issues and discussed how best to implement the research study on their particular units. All staff nurses in low participation units were involved in one 8-hour workshop and engaged in implementation of the clinical research protocol. They were responsible for assisting with patient recruitment and data collection.
**Control Group**	**Description of the Control:** No research workshops or involvement in the implementation of the clinical research protocol.<br>**Description of the Recipients:** Staff nurses.<br>**Description of Deliverer:** Not applicable.<br>**Length/Duration:** Not reported.<br>**Adherence/Fidelity:** Not applicable.
**Data Collection**	**Method:** Research Utilization Questionnaire (RUQ) developed by Champion and Leach (1986; 1989).<br>**Type of Measurement:** Self-report.<br>**Description:** Consists of 42 statements comprising of four subscales (support, attitude, availability, use). The support subscale measures the degree to which

	research use is encouraged by colleagues, administrators, and other health care professionals. The attitude subscale measures the nurses' feelings about incorporating and using research in practice. The availability subscale measures the nurses' access to research findings. The research use subscale measures the degree to which nurses feel they incorporate research into their practice decisions.<br>**Interpretation of Direction:** Statements are rated on a five point scale from 1 (strongly disagree) to 5 (strongly agree). Higher scores are reflective of positive attitude, research availability, support, and use of research findings.<br>**Reliability Details:** Reported alpha reliabilities for the subscales range from 0.84 to 0.94. In this study the Cronbach's coefficient for the support, attitude, availability, and use subscales were 0.85, 0.94, 0.91, and 0.93.<br>**Validity Details:** Not reported.
**Outcomes**	**Behaviour:** Degree to which nurses incorporate research into practice decisions.

**Study**	**Authors**: Tsai S [35]<br>**Date**: 2003<br>**Country**: Taiwan
**Objective**	To evaluate an 8-week course of research utilization training provided for nurses.
**Theoretical Framework**	Stetler and Marram (1976) model of utilizing research results which was further modified by Stetler (1994) into six stages.
**Methods**	**Design**: Non-randomized controlled trial.<br>**Sampling:** Convenience and purposive sampling.<br>**Recruitment Methods:** Four to five participants, with at least one year of working experience at the medical center, were selected from each supervising area by the authors. The controls were matched to nurses at the same institution for age, rank, and educational background.<br>**Unit of Allocation:** Individuals (nurses).<br>**Unit of Analysis:** Individuals (nurses).<br>**Differences in Baseline Characteristics:** Reported, significant.<br>**Intention to Treat Analysis:** No.
**Participants**	**Size of Eligible Population:** Not reported.<br>**Sample Size**<br>**Total:** N = 105.<br>**Intervention Group**: Not reported.<br>**Control Group:** Not reported.<br>**Hospital Characteristics:** Not reported.<br>**Description of Nurse Participants:**<br>**"Nurses":** n = 105.<br>**Age in years, mean (SD):** Intervention: 33.9 (6.0); Control: 33.9 (7.2); Overall: 33.9 (6.5).<br>**Years of Experience:** 10 years (6.9).
**Intervention Group**	**Type of Intervention:** Educational meetings (in-person, didactic, interactive).<br>**Evidence Based for Intervention:** Not applicable.<br>**Description of the Recipients:** Four to five nurses from each supervising area selected **Description of Deliverer:** Nineteen clinical experts with master's or doctorate degrees with at least 15 years of experience in areas of internal medicine, surgery, gynecology by author.<br>and obstetrics, pediatrics, or psychiatrics. Masters prepared nursing expert for 14 hours of research discussion and 2 hours per week for six weeks of discussion of a specific research topic under the guidance of experts in clinical nursing.<br>**Length/Duration:** 65hrs over 8 weeks.<br>**Adherence/Fidelity:** Not reported.<br>**Description of the Intervention:** Basic courses were 10 hours of lecture completed in a week on the six steps of Stetler's research utilization model, research topic selection, retrieval of references, evaluating research tools, and critical review of research results. Following these were 30 hours of lecture on examples of research in nursing by using six steps of Stetler's model such as studies in pressure sore, relaxation training, sterilizing procedure for

	intravenous therapy, tension headache, nursing instructions for patients with total knee replacement. The courses concluded with 14 hours of research discussion. Nurses were divided into 12 groups and every group had a 2 hours per week discussion of a specific research for 6 weeks, plus another 2 hours of practice in research reference retrieval on laser disks. The 8th week was closing, during which each nurse made an oral presentation of a draft plan of clinical implementation of research results.
**Control Group**	**Description of the Control:** No participation in the research utilization course.<br>**Description of the Recipients**: Not applicable.<br>**Description of Deliverer:** Not applicable.<br>**Length/Duration:** Not applicable.<br>**Adherence/Fidelity:** Not applicable.
**Data Collection**	**Method:** Research Participation Questionnaire (Tsai, 1998b).<br>**Type of Measurement:** Self-report.<br>**Description:** Nurses' self-report of participation in research activities over the most recent 3 years. Consists of 12 research activities, five of the items contain 5-10 minor items for a total of 33 items.<br>**Interpretation of Direction:** A 2-point scoring system is used in which 1 means participation and 0 means no participation. A higher score means greater participation in research activities.<br>**Reliability Details:** Internal consistency, KR 0.91.<br>**Validity Details:** Initial tool was modified according to the opinions of eight experts.
	**Method:** Research Utilization Questionnaire (Tsai, 2000).<br>**Type of Measurement:** Self report.<br>**Description:** Self report of whether research utilization was implemented in nursing practice and to what degree. Utilization area covered nursing administration, nursing techniques, routine nursing, and nursing records. Consists of 11 items, including one single-choice and nine multiple-choice, and one open-ended question.<br>**Interpretation of Direction:** Analyzed as "yes" or "no".<br>**Reliability Details:** Not reported.<br>**Validity Details:** Checked and confirmed by 5 clinical nurses.
**Outcomes**	**Behaviour:**1) Research participation, 2) Research utilization

**Study**	**Authors**: Wallen GR, Mitchell SA, Melnyk B, Fineout-Overholt E, Miller-Davis C, Yates J, et al. [60]<br>**Date**: 2010<br>**Country:** United States
**Objective**	The aim of the study was to evaluate the effectiveness of a structured multifaceted mentorship programme designed to implement EBP in a clinical research intensive environment.
**Theoretical Framework**	The Advancing Research & Clinical Practice through Close Collaboration (ARCC) model. The ARCC model was first conceptualized in 1999 by Melnyk as a mentorship framework to assist advanced practice nurses in implementing evidence-based practice (EBP).
**Methods**	**Design**: Mixed Methods<br>**Sampling:** Convenience (quantitative), purposive (qualitative)<br>**Recruitment Methods:** The study took place at one hospital (the National Institutes of Health Clinical Center). How the hospital was recruited/selected was not reported. The nurses who were targeted for the EBP mentor programme and survey were those who would ultimately participate in leading and/or mentoring nurses at all levels and in all specialties throughout the nursing department. For the comparison group, non-workshop attendees were stratified into clinical practice areas and then randomly selected from those areas to complete the survey. Nurses from ambulatory care clinics and day hospitals were also randomly selected for participation. Nurses were invited by email to participate in an online, quantitative survey.<br>**Unit of Allocation:** Individual<br>**Unit of Analysis:** Individual<br>**Differences in Baseline Characteristics:** Reported, Significant<br>**Intention to Treat Analysis:** Not Applicable (groups of clinicians or patients)
**Participants**	**Size of Eligible Population:** Not reported.<br>**Sample Size**<br>**Total:** N = 159 staff (quantitative), N = 18 staff (qualitative)<br>**Intervention Group**: n = 94 (quantitative)<br>**Control group:** n = 65(quantitative)<br>**Hospital Characteristics:** The National Institutes of Health Clinical Center in Bethesda, Maryland, USA. This is a 234-bed research hospital that supports the Intramural Research Program of the National Institutes of Health.<br>**Description of Nurse Participants:**<br>**“Nurses”:** n = 131 (includes staff nurses, charge nurses, clinical educators, managers, researchers, executives, consultants)<br>**CNS:** n = 11<br>**Age:** 41 to 50 years old, n = 51, > 50 years old, n = 45<br>**Years of Experience:** Not reported
**Intervention**	**Type of Intervention:** Multifaceted: 1) Educational meetings (in-person, interactive), 2) Mentorship<br>**Evidence Base for the Intervention:** The Advancing Research & Clinical Practice through Close Collaboration (ARCC) model conceptualized in 1999 by Melnyk, B. as a mentorship framework to assist advanced practice nurses in

	implementing EBP. Melnyk, B. and Fineout-Overholt, E. have further developed the model, which now serves as a guide to advance system-wide implementation and sustainability of EBP.<br>**Description of the Recipients:** Core group of nurse leaders, including senior clinical research nursing staff, Shared Governance committee chairs, clinical nurse specialists, nurse managers and nurse educators who were identified as most likely to become EBP mentors throughout the organization.<br>**Description of Deliverer:** Two of the investigators<br>**Length/Duration:** 2 days (workshop)<br>**Adherence/Fidelity:** Not reported.<br>**Description of the Intervention:** A 2-day intensive workshop (provided a general foundation to developing EBP skills among identified nurse champions needed to promote, implement, and sustain EBP. The workshop was targeted at a core group of nurse leaders, including senior clinical research nursing staff, Shared Governance committee chairs, clinical<br>nurse specialists, nurse managers and nurse educators who were identified as most likely to become EBP mentors throughout the organization. The aim of the programme was to improve attendees' EBP knowledge and skills, beliefs about the value of EBP, and the ability to implement it. The programme was also designed to develop and empower mentors by providing ongoing mentorship skill-building activities (e.g. . an EBP luncheon workshop on ways to strengthen mentorship, a holiday tea party to celebrate and support<br>EBP mentors and nurse leaders, and interactive lectures on the basics of EBP presented for the Clinical Practice Committee of the Nursing Practice Council). Tutorials designed to increase nurses' knowledge related to EBP were offered via the nursing intranet, were also implemented. EBP mentors worked with direct care nurses on clinical research units to strengthen their beliefs about the value of EBP and their ability consistently to deliver<br>evidence-based care.
**Control**	**Description of the Control:** No intervention.<br>**Description of the Recipients:** Stratified sample of nurses not registered to attend the workshop.
**Data Collection**	**Method:** EBP Implementation Scale (EBPI).<br>**Type of Outcome:** Behaviour<br>**Type of Measurement:** Self -report<br>**Description:** The EBPI is an 18-item questionnaire. For each item, respondents indicate how often they have demonstrated a particular EBP implementation behaviour over the past 8 weeks (e.g. used evidence to change my clinical practice; critically appraised evidence from a research study).<br>**Interpretation of Direction:** Responses range from 0 times over the past 8 weeks to more than eight times over the past 8 weeks. Higher total scores reflect more frequent use of EBP behaviours and skills<br>**Reliability Details:** In the present study, pretest and post-test Cronbach's alphas for the EBP Implementation Scale ranged from 0.92 to 0.94, which is comparable to previous psychometric testing.<br>**Validity Details:** Construct validity has been supported through factor

	analysis.
	**Method:** Focus groups<br>**Additional Description:** Focus groups were consisted of a convenience sample of clinical nurse specialists (n = 4), nurse managers from inpatient and ambulatory care areas (n = 9), and members of the Shared Governance Clinical Practice Committee (n = 5). Each of the three formative focused discussions included the following four questions: (a) What does EBP mean to you? (b) Where does EBP fall among priorities at NIH? (c) What needs to happen to make EBP a consistent part of the culture at NIH CC? and (d) What are the barriers to EBP at NIH CC? These qualitative questions provided a formative evaluation of participants' knowledge about the process of EBP and a general overview of the organizational readiness for implementing EBP. Content from these focused discussions was used to determine perceptions and potential organizational barriers prior to programme implementation.<br>**Type of Outcome:** Contextual factors.
**Outcomes**	**Behaviour:** Implementation of evidence-based practices.<br>**Contextual Factors**

**Study**	**Authors**: Wallin L, Rudberg A, Gunningberg L [59]<br>**Date**: 2004<br>**Country:** Sweden
**Objective**	The aim of this study was to investigate staff experiences in implementing guidelines for Swedish Kangaroo Mother Care (SKMC) in neoatal care. Specific objectives were to determine : 1) what activities were carried out by the change teams to implement the SKMC guideline? 2) how did staff perceive the change work associated with the implementation of the SKMC guideline? 3) how were the contextual conditions for the change work perceived? And 4) how did the change teams at the intervention units experience the facilitation?
**Theoretical Framework**	Promoting Action on Research Implementation in Health Services (PARIHS) framework.
**Methods**	**Design**: Qualitative (Descriptive).<br>**Sampling:** Convenience .<br>**Recruitment Methods:** 10 neonatal units located in the central region of Sweden were invited to participate in the study and four voluntarily accepted to participate. All members of the change team on each of the four units at the four hospitals were invited to the first interview. For the second interview, the nurse manager gathered unit staff available on the day of the interview. A few members of the change teams did not participate because they were on education leave or had quit their positions.
**Participants**	**Size of Eligible Population:** 4 units.<br>**Sample Size**<br>**Total:** 4 units, N = 45 staff.<br>**Hospital Characteristics:** Four county hospitals in central Sweden.<br>**Description of Nurse Participants:**<br>**RN:** n = 12; Intervention: n = 6, Control: n = 6.<br>**LPN:** n = 6; Intervention n = 4, Control: n = 2.<br>**Age in years (mean):** 39, with a range from 23 to 63 years.<br>**Years of Experience (mean):** 15, range from one to 37 years.
**Intervention**	**Type of Intervention:** Multifaceted: 1) Educational materials (printed), 2) Educational meetings, 3) Change team, 4) External facilitation.<br>**Evidence Based for Intervention:** Swedish Kangaroo Mother Care (KMC) guideline was compiled by a research team at the Department for Women's and Children's Health at Uppsala University. 5-page guideline was based on a draft World Health Organization guideline and published research on KMC.<br>**Description of the Recipients:** Staff members participating in the change teams.<br>**Description of Deliverer:** Facilitator and external facilitator.<br>**Length/Duration:** 8 months.<br>**Adherence/Fidelity:** Not reported.<br>**Description of the Intervention:** All units received the SKMC guideline at the start of the intervention phase. Nurse managers were to set up a change team (consisting of staff members involved in patient care) to work on implementation. On units randomized to the intervention, the change teams received external facilitation. The change teams met every month with the

	external facilitator (one of the investigators), with some e-mail communication occurring in between meetings. The facilitation involved elements such as identification and appraisal of evidence on KMC, unit context mapping according to the strengths-weaknesses-opportunities-threats analysis (SWOT), support of unit adaptation of the of KMC guideline.
**Control**	**Type of Intervention:** Multifaceted: 1) Educational materials (printed), 2) Change team.<br>**Evidence Based for Intervention:** Swedish Kangaroo Mother Care (KMC) guideline was compiled by a research team at the Department for Women's and Children's Health at Uppsala University. 5-page guideline was based on a draft World Health Organization guideline and published research on KMC.<br>**Description of the Recipients:** Staff members participating in the change teams.<br>**Description of Deliverer:** Not reported.<br>**Length/Duration:** 8 months.<br>**Adherence/Fidelity:** Not reported.<br>**Description of the Intervention:** All units received the SKMC guideline at the start of the intervention phase. Nurse managers were to set up a change team (consisting of staff members involved in patient care) to work on implementation. On units randomized to the control, the change teams did not receive external facilitation and were expected to manage guideline implementation without external support.
**Data Collection**	**Method:** Focus group interviews.<br>**Additional Description:** Two interview guides with nine pre-selected and open-ended questions were developed, one for the change teams and one for the staff groups. The interview guides were pilot tested on medical and nursing staff, which resulted in a clearer structure of the questions and more emphasis on asking for perceived effects of guideline implementation on staff and patients. A moderator (LG), together with an assistant moderator (AR) making field notes, led all focus groups. The moderator was a nurse researcher not familiar with KMC, while the assistant moderator was a nurse specialist in neonatology. The interviews were conducted during phase 3 of the study (September and October 2002).
**Outcomes**	**Contextual Factors**

**Study**	**Authors**: Wesorick D, Grunawalt J, Kuhn L, Rogers M, Gianchandani R [46]<br>**Date**: 2010<br>**Country:** United States
**Objective**	To investigate the effects of targeted quality improvement interventions on insulin prescribing and glycemic control.
**Theoretical Framework**	Not reported
**Methods**	**Design**: Non-randomized controlled trial.<br>**Sampling:** Convenience.<br>**Recruitment Methods:** Not reported.<br>**Unit of Allocation:** Individual (patients).<br>**Unit of Analysis:** Individual (patients).<br>**Differences in Baseline Characteristics:** Reported, significant.<br>**Intention to Treat Analysis:** Not Applicable (groups of clinicians or patients).
**Participants**	**Size of Eligible Population:** Not reported.<br>**Sample Size**<br>**Total:** N = 245 patients, 1315 patient days.<br>**Intervention Group #1:** n = 84 patients, 453 patient days.<br>**Intervention Group #2:** n = 86 patients, 471 patient days.<br>**Control Group:** n = 75 patients, 391 patient days.<br>**Hospital Characteristics:** University of Michigan Hospital.<br>**Description of Nurse Participants:** Not reported.<br>**Age:** Not reported.<br>**Years of Experience:** Not reported.
**Intervention Group #1**	**Type of Intervention:** Multifaceted: 1) Standardized Subcutaneous Insulin Order Form and 2) Educational meeting (assumed in-person for nurses + physicians).<br>**Evidence Based for Intervention:** Technical review of the literature on diabetes in the hospital setting from 2004.<br>**Description of the Recipients:** Patients cared for by attending physicians, midlevel providers, residents, and nurses in the intervention group (IG).<br>**Description of Deliverer:** All physician education was provided by the physician authors. Authors state that the nurses caring for patients in the were given education similar to that which was provided to the physicians. These attending physicians, midlevel providers, residents, and nurses delivered care to the patients in the intervention group (IG).<br>**Length/Duration:** Assumed 6 months.<br>**Adherence/Fidelity:** Not reported.<br>**Description of the Content Provided:** Referred to as the intervention group (IG). Included physician education, nurse education, and the standardized order form.<br>**Description of the Intervention:** The Standardized Subcutaneous Insulin Order Form, based on best practice guidelines, was designed to encourage physicians to prescribe insulin physiologic way. The form was developed by a multidisciplinary team and was the only way to order or modify insulin regimens in the intervention group unit. Nurses caring for patients in the

	intervention group received education similar to that for physicians. Nurse education emphasized practical issues related to delivering physiologic insulin and included topics such as blood glucose monitoring and the real-time manipulation of nutritional insulin doses. Physician education included best practice recommendations for the management of diabetes and hyperglycemia based on principles of anticipatory, physiologic insulin use.
**Intervention Group #2**	**Type of Intervention:** Educational meeting (for physicians only).<br>**Evidence Based for Intervention:** Technical review of the literature on diabetes in the hospital setting from 2004.<br>**Description of the Recipients:** Patients cared for by attending physicians, midlevel providers, and residents the concurrent control group (CCG).<br>**Description of Deliverer:** All physician education was provided by the physician authors. These attending physicians, midlevel providers, and residents delivered care to the patients in the concurrent control group (CCG).<br>**Length/Duration:** Not reported.<br>**Adherence/Fidelity:** Not reported.<br>**Description of the Intervention:** Referred to as the concurrent control group (CCG). Physician education included best practice recommendations for the management of diabetes and hyperglycemia based on principles of anticipatory, physiologic insulin use.
**Control Group**	**Description of the Control:** No receipt of any interventions.<br>**Description of the Recipients:** Patients cared for by attending physicians, midlevel providers, residents, and nurses in the historic control group (HCG).<br>**Description of Deliverer:** Attending physicians, midlevel providers, residents, and nurses not receiving any intervention delivered care to the patients in the historic control group (HCG), in the 2 years prior to the intervention.<br>**Length/Duration:** Not reported.<br>**Adherence/Fidelity:** Not applicable.
**Data Collection**	**Method:** Chart Audit.<br>**Type of Measurement:** Subjective.<br>**Description:** The primary unit of measure was the patient-da (all of the information for 1 patient on a single qualifying day). Each patient day was categorized as in-range (70-180 mg/dl), hyperglycemic (> 180 mg/dl), severely hyperglycemic (> 250 mg/dl), hypoglycemic (< 70 mg/dl), and/or severely hypoglycemic (<50 mg/dl). The day weighted mean blood glucose value was calculated and the mean blood glucose for each patient-day and the assigned these values for each group.<br>**Interpretation of Direction:** Higher levels indicate poor glycemic control.<br>**Reliability Details:** Not reported.<br>**Validity Details:** Not reported.
**Outcomes**	**Client:** Glycemic control

Study	**Authors**: Weber S [53]<br>**Date**: 2007<br>**Country:** United States
**Objective**	The objective of this study was to generate a grounded theory to increase understanding of APNs' experiences with computerized clinical decision systems in critical care settings.
**Theoretical Framework**	Strauss and Corbin.
**Methods**	**Design**: Qualitative (Grounded Theory).<br>**Sampling:** Convenience.<br>**Recruitment Methods:** Participants were recruited from 16 critical care units in six large urban medical centers in the U.S. Midwest. Selection criteria included the following: (a) national certification as an NP or a CNS, (b) practice privileges in one or more of the 16 critical care nursing or intensive care units (ICUs) located in one of the six medical center research sites, (c) completion of at least one method of education or training related to a clinical decision support system (formal classroom, one on one, or self-study), (d) have clinical access to one or more of the clinical decision support systems in operation at one or more of the research sites, and (e) able to speak English.
**Participants**	**Size of Eligible Population:** Not reported.<br>**Sample Size:** 23 individual interviews.<br>**Hospital Characteristics:** 16 critical care units in six large urban medical centers in the U.S. Midwest.<br>**Description of Nurse Participants:**<br>**NP:** 10.<br>**CNS:** 13.<br>**Age:** Not reported.<br>**Years of Experience:** Not reported.
**Intervention**	**Type of Intervention:** Clinical Decision Support System.<br>**Evidence Based for Intervention:** A review of the strategic initiatives of patient care managers, including advanced practice nurses, indicated that the implementation of CDSS within a facility or across a system of care is carried out to provide healthcare professionals with an additional source of information for patient care decision making.<br>**Description of the Recipients:** Assume health care professionals, including NPs and CNSs.<br>**Description of Deliverer:** Not applicable due to nature of the intervention.<br>**Length/Duration:** Not reported.<br>**Adherence/Fidelity:** Not reported.<br>**Description of the Intervention:** Not reported. All that was reported was that to be a participant in the study, NPs and CNSs had to have clinical access to one or more of the clinical decision support systems in<br>operation at one or more of the research sites and have completed of at least one method of education or training related to a clinical decision support system (formal classroom, one on one, or self-study).
**Data Collection**	**Method:** Semi-structured interviews.<br>**Additional Details:** Questions only guided the interview process; participants were free to discuss any of their experiences and perceptions related to the use of the clinical decision system in the critical care<br>setting.
**Outcomes**	**Contextual Factors**

References

32. Daly M, Kermode S, Reilly D. Evaluation of clinical practice improvement programs for nurses for the management of alcohol withdrawal in hospitals. Contemp Nurse. 2009;31(2):98-107.

33. Kirshbaum M. Translation to practice: A randomised, controlled study of an evidence-based booklet for breast-care nurses in the United Kingdom. Worldviews Evid Based Nurs. 2008;5(2):60-74.

34. Girouard S. The role of the clinical specialist as change agent: An experiment in preoperative teaching. Int J Nurs Stud. 1978;15(2):57-65.

35. Tsai Sl. The effects of a research utilization in-service program on nurses. Int J Nurs Stud. 2003;40(2):105-13.

36. Middleton S, McElduff P, Ward J, Grimshaw JM, Dale S, D'Este C, et al. Implementation of evidence-based treatment protocols to manage fever, hyperglycaemia, and swallowing dysfunction in acute stroke (QASC): A cluster randomised controlled trial. Lancet. 2011;378(9804):1699-706.

37. Melnyk BM, Bullock T, McGrath J, Jacobson D, Kelly S, Baba L. Translating the evidence-based NICU COPE program for parents of premature infants into clinical practice: impact on nurses' evidence-based practice and lessons learned. J Perinat Neonatal Nurs. 2010;24(1):74-80.

38. Titler M. Translating research into practice. Am J Nurs. 2007;107(6):26-33.

39. Day T, Wainwright SP, Wilson-Barnett J. An evaluation of a teaching intervention to improve the practice of endotracheal suctioning in intensive care units. J Clin Nurs. 2001;10(5):682-96.

40. Hyndman KJ. An evaluation of a dissemination intervention to enhance registered nurses' use of clinical practice guidelines related to tobacco reduction University of British Columbia (Canada); 2005.

41. Seers K, Crichton N, Carroll D, Richards S, Saunders T. Evidence-based postoperative pain management in nursing: Is a randomized-controlled trial the most appropriate design? J Nurs Manag. 2004;12(3):183-93.

42. Tranmer JE, Lochhaus-Gerlach J, Lam M. The effect of staff nurse participation in a clinical nursing research project on attitude towards, access to, support of and use of research in the acute care setting. Can J Nurs Leadersh. 2002;15(1):18-26.

43. Lewicki LJ. Diffusion of pressure ulcer guidelines: Testing an intervention Case Western Reserve University (Health Sciences); 1997.

44. Linde BJ. The effectiveness of three interventions to increase research utilization among practicing nurses University of Michigan; 1989.

45. Fan J, Woolfrey K. The effect of triage-applied Ottawa ankle rules on the length of stay in a Canadian urgent care department: A randomized controlled trial. Acad Emerg Med. 2006;13(2):153-7.

46. Wesorick DH, Grunawalt J, Kuhn L, Rogers MAM, Gianchandani R. Effects of an educational program and a standardized insulin order form on glycemic outcomes in non-critically ill hospitalized patients. J Hosp Med. 2010;5(8):438-45.

47. Sulch D, Perez I, Melbourn A, Kalra L. Randomized controlled trial of integrated (managed) care pathway for stroke rehabilitation. Stroke. 2000;31(8):1929-34.

48. Manias E, Gibson SJ, Finch S. Testing an educational nursing intervention for pain assessment and management in older people. Pain Med. 2011;12(8):1199-215.

49. Dykes PC, Carroll DL, Hurley A, Lipsitz S, Benoit A, Chang F, et al. Fall prevention in acute care hospitals: A randomized trial. JAMA. 2010;304(17):1912-8.

50. Stetler CB, Caramanica L. Evaluation of an evidence-based practice initiative: outcomes, strengths and limitations of a retrospective, conceptually-based approach. Worldviews Evid Based Nurs. 2007;4(4):187-99.

51. Gifford WA, Davies B, Edwards N, Graham ID. Leadership strategies to influence the use of clinical practice guidelines. Can J Nurs Leadersh. 2006;19(4):72-88.

52. Ploeg J, Davies B, Edwards N, Gifford W, Miller PE. Factors influencing best-practice guideline implementation: Lessons learned from administrators, nursing staff, and project leaders. Worldviews Evid Based Nurs. 2007;4(4):210-9.

53. Weber S. A qualitative analysis of how advanced practice nurses use clinical decision support systems. J Am Acad Nurse Pract. 2007;19(12):652-67.

54. Happell B, Johnston L, Hill C. Implementing research findings into mental health nursing practice: Exploring the clinical research fellowship approach. Int J Ment Health Nurs. 2003;12(4):251-8.

55. Happell B, Martin T. Changing the culture of mental health nursing: The contribution of nursing clinical development units. Issues Ment Health Nurs. 2005;26(9):921-33.

56. Kajermo KN, Nordström G, Krusebrant A, Lutzén K. Nurses' experiences of research utilization within the framework of an educational programme. J Clin Nurs. 2001;10(5):671-81.

57. Royle JA, Blythe J, Ingram C, DiCenso A, Bhatnager N, Potvin C. The research utilization process: the use of guided imagery to reduce anxiety. Can Oncol Nurs J. 1996;6(1):20-5.

58. Ellis I, Howard P, Larson A, Robertson J. From workshop to work practice: An exploration of context and facilitation in the development of evidence-based practice. Worldviews Evid Based Nurs. 2005;2(2):84-93.

59. Wallin L, Rudberg A, Gunningberg L. Staff experiences in implementing guidelines for Kangaroo Mother Care - A qualitative study. Int J Nurs Stud. 2005;42(1):61-73.

60. Wallen GR, Mitchell SA, Melnyk B, Fineout-Overholt E, Miller-Davis C, Yates J, et al. Implementing evidence-based practice: Effectiveness of a structured multifaceted mentorship programme. J Adv Nurs. 2010;66(12):2761-71.

61. Graham ID, Logan J, Davies B, Nimrod C. Changing the use of electronic fetal monitoring and labor support: A case study of barriers and facilitators. Birth. 2004;31(4):293-301.

64. Middleton S, Levi C, Ward J, Grimshaw J, Griffiths R, D'Este C, et al. Fever, hyperglycaemia and swallowing dysfunction management in acute stroke: a cluster randomised controlled trial of knowledge transfer. Implement Sci. 2009;4:16.

65. Middleton S, Levi C, Ward J, Grimshaw J, Griffiths R, D'Este C, et al. Death, dependency and health status 90 days following hospital admission for acute stroke in NSW. Intern Med J. 2011;41(10):736-43.

67. Edwards N, Davies B, Ploeg J, Dobbins M, Skelly J, Griffin P, et al. Evaluating best practice guidelines. Can Nurse. 2005;101(2):18-23.

68. Davies B, Hodnett E, Hannah M, O'Brien-Pallas L, Pringle D, Wells G, et al. Fetal health surveillance: A community-wide approach versus a tailored intervention for the implementation of clinical practice guidelines. CMAJ. 2002;167(5):469-74.
