# Supplementary material for: The effectiveness of knowledge translation interventions for promoting evidence-informed decision-making among nurses in tertiary care: a systematic review and meta-analysis
Source: Implement Sci. 2015 Jul 14;10:98. doi: 10.1186/s13012-015-0286-1 (PMC4499897; doi:10.1186/s13012-015-0286-1)
Supplement: Additional file 4: — Quality assessment details. This file provides the risk of bias assessment for the included studies. This includes the review author’s judgements about each quality criteria for each of the final included studies. [file 13012_2015_286_MOESM4_ESM.pdf]

Additional file 4 – Quality assessment details

**Quantitative Studies** (*Cochrane Risk of Bias criteria [19]*)							
**Study**	**Random Sequence Generation**	**Allocation Concealment**	**Blinding of Participants & Personnel**	**Blinding of Outcome Assessment**	**Incomplete Outcome Data**	**Selective Reporting**	**Other Bias***
Daly, et al. [32] [d]	Unclear	Unclear	High Risk	Unclear	Unclear	Low Risk	High Risk
Day, et al. [39] [a]	Unclear	Unclear	High Risk	Low Risk	Low Risk	Low Risk	High Risk
Dykes, et al. [49] [b]	Unclear	Unclear	Unclear	Unclear	Low Risk	Low Risk	High Risk
Fan & Woolfrey [45] [a]	Low Risk	Low Risk	Unclear	Low Risk	Not applicable	Low Risk	Low Risk
Girouard [34] [a]	Unclear	Unclear	High Risk	Unclear (S); Unclear (SR)	High Risk (S); Low Risk (SR)	Low Risk	High Risk
Hyndman [40] [d]	High Risk	High Risk	High Risk	Unclear	Low Risk	Low Risk	High Risk
Kirshbaum [33]	Unclear	Unclear	Unclear	Unclear	Unclear	Low Risk	High risk
Lewicki [43] [a]	Low Risk	Low Risk	High Risk	Unclear	High Risk	Low Risk	High Risk
Linde [44] [b]	Unclear	Unclear	High Risk	High Risk	High Risk	Low Risk	High Risk
Manias, et al. [48] [d]	Unclear	Unclear	Low Risk	High Risk	High Risk	Low Risk	High Risk
Middleton, et al. [36, 64-65] [b]	Low Risk	Low Risk	High Risk	Low Risk	Low Risk	Low Risk	Low Risk
Melynk, et al. [37] [c]	High Risk	High Risk	High Risk	High Risk	Unclear	Low Risk	High Risk
Seers, et al. [41] [b]	Unclear	Unclear	High Risk	Unclear	Not applicable	Low Risk	High Risk
Sulch, et al. [47] [a]	Low Risk	Low Risk	High Risk	Unclear	Low Risk	Low Risk	High Risk
Titler et al. [38] [b]	Unclear	Unclear	Low Risk	Unclear	Not applicable	Low Risk	Low Risk
Tranmer, et al. [42] [d]	Unclear	Unclear	High Risk	High Risk	High Risk	Low Risk	High Risk
Tsai [35] [c]	High Risk	Unclear	High Risk	Unclear	Low Risk	Low Risk	High Risk
Wesorick, et al. [46] [c]	High Risk	High Risk	High Risk	Unclear	Not applicable	Low Risk	High Risk

* Other bias included: industry funding/involvement in the study; unadjusted baseline difference in outcome(s); insufficient power; unit of analysis issues; participation rate < 80%; different participants at all data points (not applicable for groups of patients); possibility for co-intervention and/or contamination.
[a] Randomized control trial (RCT); [b] Cluster RCT; [c] Non-randomized trial; [d] Cluster non-randomized trial
S: Subjective outcome; SR: Self-report outcome

Additional file 4 – Quality assessment details

**Qualitative Studies** *(Joanna Briggs Institute Qualitative Assessment and Review Instrument (QARI) [20])*										
**Study**	**Congruity between the stated philosophical perspective & research methodology**	**Congruity between research methods & question/ objectives**	**Congruity between research methods & data collection methods**	**Congruity between research methods & represent-ation & data analysis**	**Congruity between research methods & interpret-ation of results**	**There is a statement locating the researcher culturally or theoretic-ally**	**The influence of the researcher on the research & vice-versa is addressed**	**Participants & their voices are adequately represented**	**The research is ethical; there is evidence of ethical approval by an appropriate body**	**Conclusions drawn in the report appear to flow from the analysis, or data interpret-ation**
Ellis, et al. [58]	Yes	Yes	Yes	Yes	Yes	Yes	No	Yes	Unclear	Yes
Gifford, et al. [51]; Edwards, et al. [67]	Yes	Yes	Unclear	Yes	Yes	Yes	No	Yes	Yes	Yes
Happell & Martin [55]	Unclear	Yes	Yes	Yes	Yes	No	No	Yes	Unclear	Yes
Happell, et al. [54]	Yes	Yes	Yes	Yes	Yes	No	No	Yes	Yes	Yes
Kajermo et al. [56]	No	Yes	Yes	Yes	Yes	No	Yes	Yes	Yes	Yes
Ploeg, et al. [52]; Edwards, et al. [67]	Unclear	Yes	Yes	Yes	Yes	No	No	Yes	Yes	Yes
Royle, et al. [57]	Yes	Yes	Yes	Yes	Yes	No	No	Yes	Unclear	Yes
Stetler, & Caramani-ca [40]	Yes	Yes	Yes	Yes	Yes	Yes	Yes	Yes	Yes	Yes

Wallin et al. [55]	Unclear	Unclear	Unclear	Unclear	Yes	No	No	Yes	Yes	Yes
Weber [49]	Yes	Yes	Yes	Yes	Yes	No	Yes	Yes	Yes	Yes

**Mixed Methods Studies**										
	**Quantitative** *(Cochrane Risk of Bias criteria [19])*									
**Study**	**Random Sequence Generation**	**Allocation Concealment**	**Blinding of Participants & Personnel**	**Blinding of Outcome Assessment**	**Incomplete Outcome Data**	**Selective Reporting**	**Other Bias**			
Davies et al. [68]; Graham et al. [61] [c]	Unclear	Unclear	High Risk	Low Risk	Unclear	Low Risk	High Risk			
Wallen et al. [60] [c]	High Risk	High Risk	High Risk	Unclear	High Risk	Low Risk	High Risk			
	**Qualitative** *(Joanna Briggs Institute Qualitative Assessment and Review Instrument (QARI) [20])*									
	**Congruity between the stated philosophical perspective & research methodology**	**Congruity between research methods & question/ objectives**	**Congruity between research methods & data collection methods**	**Congruity between research methods & represent-ation & data analysis**	**Congruity between research methods & interpret-ation of results**	**There is a statement locating the researcher culturally or theoretic-ally**	**The influence of the researcher on the research & vice-versa is addressed**	**Participants & their voices are adequately represented**	**The research is ethical; there is evidence of ethical approval by an appropriate body**	**Conclusions drawn in the report appear to flow from the analysis, or data interpret-ation**
Davies et al. [68]; Graham et al. [61]	Unclear	Unclear	Unclear	Unclear	Unclear	Unclear	No	Unclear	Yes	Yes
Wallen et al. [60]	Unclear	Unclear	Unclear	Unclear	Unclear	No	No	No	Yes	Unclear
[c] Non-randomized trial										

Additional file 4 – Quality assessment details

References

19. Higgins Julian PT, Altman AM, Sterne JAC, Cochrane Statistical Methods Group and the Cochrane Bias Methods Group. Assessing risk of bias in included studies. In: Higgins Julian PT, Green S, editors. Cochrane Handbook for Systematic Reviews of Interventions Version 5.1.0 (updated March 2011).Chichester, UK: John Wiley & Sons; 2011.

20. SUMARI: user manual: version 5.0 system for the unified management, assessment and review of information [computer program]. 2013.

32. Daly M, Kermode S, Reilly D. Evaluation of clinical practice improvement programs for nurses for the management of alcohol withdrawal in hospitals. Contemp Nurse. 2009;31(2):98-107.

33. Kirshbaum M. Translation to practice: A randomised, controlled study of an evidence-based booklet for breast-care nurses in the United Kingdom. Worldviews Evid Based Nurs. 2008;5(2):60-74.

34. Girouard S. The role of the clinical specialist as change agent: An experiment in preoperative teaching. Int J Nurs Stud. 1978;15(2):57-65.

35. Tsai Sl. The effects of a research utilization in-service program on nurses. Int J Nurs Stud. 2003;40(2):105-13.

36. Middleton S, McElduff P, Ward J, Grimshaw JM, Dale S, D'Este C, et al. Implementation of evidence-based treatment protocols to manage fever, hyperglycaemia, and swallowing dysfunction in acute stroke (QASC): A cluster randomised controlled trial. Lancet. 2011;378(9804):1699-706.

37. Melnyk BM, Bullock T, McGrath J, Jacobson D, Kelly S, Baba L. Translating the evidence-based NICU COPE program for parents of premature infants into clinical practice: impact on nurses' evidence-based practice and lessons learned. J Perinat Neonatal Nurs. 2010;24(1):74-80.

38. Titler M. Translating research into practice. Am J Nurs. 2007;107(6):26-33.

39. Day T, Wainwright SP, Wilson-Barnett J. An evaluation of a teaching intervention to improve the practice of endotracheal suctioning in intensive care units. J Clin Nurs. 2001;10(5):682-96.

40. Hyndman KJ. An evaluation of a dissemination intervention to enhance registered nurses' use of clinical practice guidelines related to tobacco reduction University of British Columbia (Canada); 2005.

41. Seers K, Crichton N, Carroll D, Richards S, Saunders T. Evidence-based postoperative pain management in nursing: Is a randomized-controlled trial the most appropriate design? J Nurs Manag. 2004;12(3):183-93.

42. Tranmer JE, Lochhaus-Gerlach J, Lam M. The effect of staff nurse participation in a clinical nursing research project on attitude towards, access to, support of and use of research in the acute care setting. Can J Nurs Leadersh. 2002;15(1):18-26.

43. Lewicki LJ. Diffusion of pressure ulcer guidelines: Testing an intervention Case Western Reserve University (Health Sciences); 1997.

44. Linde BJ. The effectiveness of three interventions to increase research utilization among practicing nurses University of Michigan; 1989.

45. Fan J, Woolfrey K. The effect of triage-applied Ottawa ankle rules on the length of stay in a Canadian urgent care department: A randomized controlled trial. Acad Emerg Med. 2006;13(2):153-7.

46. Wesorick DH, Grunawalt J, Kuhn L, Rogers MAM, Gianchandani R. Effects of an educational program and a standardized insulin order form on glycemic outcomes in non-critically ill hospitalized patients. J Hosp Med. 2010;5(8):438-45.

47. Sulch D, Perez I, Melbourn A, Kalra L. Randomized controlled trial of integrated (managed) care pathway for stroke rehabilitation. Stroke. 2000;31(8):1929-34.

48. Manias E, Gibson SJ, Finch S. Testing an educational nursing intervention for pain assessment and management in older people. Pain Med. 2011;12(8):1199-215.

49. Dykes PC, Carroll DL, Hurley A, Lipsitz S, Benoit A, Chang F, et al. Fall prevention in acute care hospitals: A randomized trial. JAMA. 2010;304(17):1912-8.

50. Stetler CB, Caramanica L. Evaluation of an evidence-based practice initiative: outcomes, strengths and limitations of a retrospective, conceptually-based approach. Worldviews Evid Based Nurs. 2007;4(4):187-99.

51. Gifford WA, Davies B, Edwards N, Graham ID. Leadership strategies to influence the use of clinical practice guidelines. Can J Nurs Leadersh. 2006;19(4):72-88.

52. Ploeg J, Davies B, Edwards N, Gifford W, Miller PE. Factors influencing best-practice guideline implementation: Lessons learned from administrators, nursing staff, and project leaders. Worldviews Evid Based Nurs. 2007;4(4):210-9.

53. Weber S. A qualitative analysis of how advanced practice nurses use clinical decision support systems. J Am Acad Nurse Pract. 2007;19(12):652-67.

54. Happell B, Johnston L, Hill C. Implementing research findings into mental health nursing practice: Exploring the clinical research fellowship approach. Int J Ment Health Nurs. 2003;12(4):251-8.

55. Happell B, Martin T. Changing the culture of mental health nursing: The contribution of nursing clinical development units. Issues Ment Health Nurs. 2005;26(9):921-33.

56. Kajermo KN, Nordström G, Krusebrant A, Lutzén K. Nurses' experiences of research utilization within the framework of an educational programme. J Clin Nurs. 2001;10(5):671-81.

57. Royle JA, Blythe J, Ingram C, DiCenso A, Bhatnager N, Potvin C. The research utilization process: the use of guided imagery to reduce anxiety. Can Oncol Nurs J. 1996;6(1):20-5.

58. Ellis I, Howard P, Larson A, Robertson J. From workshop to work practice: An exploration of context and facilitation in the development of evidence-based practice. Worldviews Evid Based Nurs. 2005;2(2):84-93.

59. Wallin L, Rudberg A, Gunningberg L. Staff experiences in implementing guidelines for Kangaroo Mother Care - A qualitative study. Int J Nurs Stud. 2005;42(1):61-73.

60. Wallen GR, Mitchell SA, Melnyk B, Fineout-Overholt E, Miller-Davis C, Yates J, et al. Implementing evidence-based practice: Effectiveness of a structured multifaceted mentorship programme. J Adv Nurs. 2010;66(12):2761-71.

61. Graham ID, Logan J, Davies B, Nimrod C. Changing the use of electronic fetal monitoring and labor support: A case study of barriers and facilitators. Birth. 2004;31(4):293-301.

64. Middleton S, Levi C, Ward J, Grimshaw J, Griffiths R, D'Este C, et al. Fever, hyperglycaemia and swallowing dysfunction management in acute stroke: a cluster randomised controlled trial of knowledge transfer. Implement Sci. 2009;4:16.

65. Middleton S, Levi C, Ward J, Grimshaw J, Griffiths R, D'Este C, et al. Death, dependency and health status 90 days following hospital admission for acute stroke in NSW. Intern Med J. 2011;41(10):736-43.

67. Edwards N, Davies B, Ploeg J, Dobbins M, Skelly J, Griffin P, et al. Evaluating best practice guidelines. Can Nurse. 2005;101(2):18-23.

68. Davies B, Hodnett E, Hannah M, O'Brien-Pallas L, Pringle D, Wells G, et al. Fetal health surveillance: A community-wide approach versus a tailored intervention for the implementation of clinical practice guidelines. CMAJ. 2002;167(5):469-74.
