# Supplementary material for: The effectiveness of knowledge translation interventions for promoting evidence-informed decision-making among nurses in tertiary care: a systematic review and meta-analysis
Source: Implement Sci. 2015 Jul 14;10:98. doi: 10.1186/s13012-015-0286-1 (PMC4499897; doi:10.1186/s13012-015-0286-1)
Supplement: Additional file 5: — Outcomes tables. This file provides the citation, measurement period, brief description of the intervention and control groups, outcome measurements at baseline and follow-up, and overall effect estimates and confidence intervals for included studies with quantitative data. [file 13012_2015_286_MOESM5_ESM.pdf]

Additional file 5 - Outcomes tables

Engaging in EIDM Behaviours						
**Study**	**Outcome**	**Groups**	**Baseline**	**Interim**	**Follow-up**	**Overall Effect**
Melynk, et al. [37]	Implementation of evidence-based practice	I:<br>n = 25<br>*Multifaceted*<br><br>C:<br>n = 18<br>*No intervention*	Mean (SD):<br>7.37 (12.1)<br><br>Mean (SD):<br>5.95 (6.1)		Mean (SD):<br>11.42 (10.8)<br><br>Mean (SD):<br>8.25 (8.0)<br><br>T: 6 months	I versus C<br>Baseline: *P* = 0.16<br>Follow-up: MD 1.75*, 95% CI (-4.63, 8.13)*<br><br>Interpretation of direction: Total score ranges from 0-60. Higher scores indicate greater EIDM behaviours.
Tsai et al. [35]	Research participation	I:<br>n = 47<br>*Multifaceted*<br><br>C:<br>n = 42<br>*No intervention*	Median:<br>6.00<br><br>Median:<br>5.80	Median:<br>8.00<br><br>Median:<br>4.00<br><br>T: post-intervention	Median:<br>9.00<br><br>Median:<br>4.50<br><br>T: 6 months	I versus C<br>Baseline: *P* = 0.944<br>Interim: MD 4.00*, 95% CI (0.55, 7.45)* *P* = 0.02*<br>Follow-up: MD 4.50*, 95% CI (1.05, 7.95)* *P* = 0.01*<br><br>Interpretation of direction: Total score ranges from 0-33. Higher score indicates greater participation in research activities
	Use of research results	I:<br>n = 47<br>*Multifaceted*<br><br>C:<br>n = 42<br>*No intervention*	% yes:<br>46.8%<br><br>% yes:<br>42.9%	% yes:<br>42.6%<br><br>% yes:<br>40.5%<br><br>T: post-intervention	% yes:<br>51.1%<br><br>% yes:<br>57.1%<br><br>T: 6 months	I versus C<br>Baseline: *P* = 0.708<br>Interim: RR 1.05*, 95% CI (0.64, 1.72)*, *P* =0.84*<br>Follow-up: RR 0.89*, 95% CI (0.61, 1.31)*, *P* =0.57*<br><br>Interpretation of direction: Total of 11 items scored as "yes" or "no". Higher percentage indicates greater use of research results.

Tranmer, et al. [42]	Incorporate research evidence into practice decisions	I1:<br>n = 37 (baseline)<br>n = 29 (follow-up)<br>*Multifaceted*<br><br>I2:<br>n = 21 (baseline)<br>n = 39 (follow-up)<br>*Multifaceted*<br><br>Control:<br>n = 34 (baseline)<br>n = 24 (follow-up)<br>*No intervention*	Mean (SD):<br>3.40 (0.52)<br><br>Mean (SD):<br>2.98 (0.70)<br><br>Mean (SD):<br>3.20 (0.69)		Mean (SD):<br>3.46 (0.71)<br><br>Mean (SD):<br>3.03 (0.68)<br><br>Mean (SD):<br>3.07 (0.69)<br><br>T: 12 months	I1 versus C<br>Baseline: $P < 0.05$<br>Follow-up: MD 0.26*, (95% CI -0.12, 0.64*), $P = 0.18$*<br><br>I2 versus C<br>Baseline: $P \geq 0.05$<br>Follow-up: MD 0.17*, 95% CI -0.52, 0.18) *, $P = 0.34$*<br><br>I1 versus I2<br>Baseline: $P < 0.05$<br>Follow-up: MD: 0.43*, 95% CI (0.09, 0.77)*, $P = 0.01$*<br><br>Interpretation of direction: 42 items rated on a five point scale from 1 (strongly disagree) to 5 (strongly agree). Total score ranges from 42 to 210. Higher scores indicate greater positive attitude, research availability, support, and use of research findings.
Wallen, et al. [60]	Implementation of evidence-based practice	I:<br>n = 54<br>*Multifaceted*<br><br>C:<br>n = 35<br>*No intervention*	Mean (SD):<br>34.3 (13.9)<br><br>Mean (SD):<br>29.7 (8.9)		Mean (SD):<br>40.9 (16.9)<br><br>Mean (SD):<br>32.7 (11.9)<br><br>T: 6 months	I versus C<br>Baseline: $P \geq 0.05$<br>Follow-up: MD: 3.6*, 95% CI (-2.60, 9.80)*<br><br>Interpretation of direction: Total score ranges from 0-60. Higher scores indicate greater EIDM behaviours.
* calculated using study data by review team, interim or follow-up only<br>Abbreviations: I: Intervention; C: Control; T: Timeframe; CI: Confidence Interval, MD: Mean difference; AD: Absolute difference; RR: Relative Risk						

Use of Research Evidence for Practice Change						
**Study**	**Outcome**	**Groups**	**Baseline**	**Interim**	**Follow-up**	**Overall Effect**
Daly, et al. [32]	Compliance rate with protocol for alcohol management	I:<br>n = 17 (baseline)<br>n = 65 (follow-up)<br>*Educational materials*<br><br>C: n = 83 (baseline); n = 175 (follow-up)<br>*Education meeting*	% yes:<br>59%<br><br><br><br>% yes:<br>57%		% yes:<br>84%<br><br><br><br>% yes:<br>66%<br><br>T: 1-2 years	I versus C:<br>Baseline: Not reported.<br>Follow-up: RR: 1.28*, 95% CI (1.10, 1.48)*, *P* =0.001*<br><br>Interpretation of direction: Nine standards judged for implementation ('yes', 'no',or n/a'). Higher percentage indicates greater compliance.
Day, et al. [39]	Performance of research based endotracheal suctioning techniques	I:<br>n = 8<br>*Educational meeting*<br><br>C:<br>n = 8<br>*Educational meeting*		Mean: 22.37<br><br><br><br>Mean: 11.81<br><br>T: 4 days	Mean: 21.00<br><br><br><br>Mean: 11.12<br><br>T: 4 weeks	I versus C<br>Baseline: *P* = 0.36<br>Interim: MD 10.56*, 95% CI (4.10, 17.0)*<br>Follow-up: MD 9.88*, 95% CI (3.42, 16.34)*, *P* = 0.003*<br><br>Interpretation of direction: Higher scores indicate greater performance of techniques.
Giround [34]	Performance of preoperative teaching activities	I:<br>n = 20<br>*Multifaceted*<br><br>Control:<br>n = 16<br>*No intervention*	Mean (SD):<br>104.85 (10.13)<br><br><br>Mean (SD):<br>106.94 (5.55)		Mean (SD):<br>103.3 (6.67)<br><br><br>Mean (SD):<br>105.56 (6.67)<br><br>T: 4 weeks	I versus C<br>Baseline: Non-significant<br>Follow-up: MD -2.26*, 95% CI (-6.64, 2.1)*, *P* = 0.31*<br><br>Interpretation of direction: Total score ranges from 28-140. Higher scores indicate greater performance of activities.
	Document-ation of preoperative teaching activities	I: n = 10<br>*Multifaceted*<br><br><br><br>Control: n = 10<br>*No intervention*	0-4 items:<br>n = 4<br>5-9 items:<br>n = 6<br><br>0-4 items:<br>n = 3<br>5-9 items:<br>n = 7		Mean (SD):<br>5.6 (2.27)<br><br><br><br>Mean (SD):<br>2.8 (1.48)<br><br>T: 4 weeks	I versus C:<br>Baseline: Non-significant<br>Follow-up: MD 2.80*, 95% CI (1.57, 4.03)*, *P* < 0.00001*<br><br>Interpretation of direction: Higher scores indicate greater documented teaching.

Hyndman [40]	Adherence to guideline on treating tobacco use & dependence	I: n= 67<br>*Multifaceted*<br><br>C: n = 71<br>*Multifaceted*	Mean (SD):<br>21.7 (7.4)<br>95% CI:<br>(19.9, 25.5)<br><br>Mean (SD):<br>19.8 (7.7)<br>95% CI:<br>(18.0, 21.5)		Mean:<br>37.6<br>95% CI:<br>(35.3, 39.9)<br><br>Mean:<br>21.1<br>95% CI:<br>(19.0, 23.3)<br><br>T: 3 weeks	I versus C:<br>Baseline: Non-significant.<br>Follow-up: MD: 6.50*, 95% CI (3.58, 9.42)*, *P* < 0.0001*<br><br>Interpretation of direction: Total score ranges from 12-60 [scored scale from never (0 smokers out of 10) to usually (9-10 smokers out of 10)]. Higher scores indicate greater adherence.
Kirschbaum, et al. [33]	Make recommend-ations for exercise according , the evidence	I:<br>n = 51 (follow-up)<br>*Educational materials*<br><br>C:<br>n = 41 (follow-up)<br>*No intervention*			T: 2 months	I versus C<br>Baseline: Non-significant<br>Follow-up:<br>For nausea: OR 2.54, 95% CI (2.53, 13.20)<br>For loss of appetite: OR 3.67, 95% CI (1.82, 8.76)<br>For fatigue: OR 2.4, 95% CI (1.12, 5.99)<br>For weight gain: OR 1.55, 95% CI (0.73, 3.03)<br>For insomnia: OR 1.46, 95% CI (0.64, 3.60)<br>For loss of libido: OR 1.92, 95% CI (0.94, 3.64)<br>For panic attacks: OR 2.23, 95% CI (0.89, 5.75)<br>For altered body image: OR 1.62, 95% CI (0.67, 3.82)<br>For headaches: OR 2.41, 95% CI (0.98, 5.42)<br>For altered body image: OR 1.62, 95% CI (0.67, 3.82)<br><br>Interpretation of direction: Odds ratios (> 1.0) are associated with making greater recommendations.

Lewicki [43]	Performance of Braden Scores on admission	I1:<br>n = 32<br>*Multifaceted (Individual feedback)*<br><br>I2:<br>n = 35<br>*Multifaceted (Group feedback)*<br><br>C:<br>n = 29<br>*Multifaceted*	Mean (SD):<br>43.58 (31.04)<br><br>Mean (SD):<br>48.98 (29.45)<br><br>Mean (SD):<br>49.70 (33.88)		Mean (SD):<br>71.84 (28.55)<br><br>Mean (SD):<br>70.62 (31.03)<br><br>Mean (SD):<br>64.12 (26.22)<br><br>T: 1 week	I1 versus C:<br>Baseline: Non-significant**<br>Follow-up: MD 7.22*, 95% CI (-6.02, 21.46)*, *P* = 0.27*<br><br>I2 versus C<br>Baseline: Non-significant**<br>Follow-up: MD: 6.50*, 95% CI (-7.53, 20.53) *, *P* = 0.36*<br><br>I1 versus I2<br>Baseline: Non-significant**<br>Follow-up: MD 1.22*, 95% (-13.05, 15.49)*, *P* = 0.87*<br><br>Interpretation of direction: Total score not reported. Higher score indicates greater performance.
	At-risk patients receiving prevention interventions	I1: n = 32<br>*Multifaceted*<br><br>I2: n = 35<br>*Multifaceted*<br><br>C: n = 29<br>*Multifaceted*	Mean (SD):<br>36.80 (29.18)<br><br>Mean (SD):<br>49.50 (32.85)<br><br>Mean (SD):<br>35.19 (34.14)		Mean (SD):<br>69.10 (24.25)<br><br>Mean (SD):<br>73.30 (22.41)<br><br>Mean (SD):<br>65.80 (30.36)<br><br>T: 1 week	I1 versus C<br>Baseline: Non-significant**<br>Follow-up: MD 3.30*, 95% CI (-10.58, 17.18)*, *P* = 0.64*<br><br>I2 versus C<br>Baseline: Non-significant**<br>Follow-up: MD 7.50*, 95% CI (-5.81, 20.81)*, *P* = 0.27*<br><br>I1 versus I2<br>Baseline: Non-significant**<br>Follow-up: MD: 4.20*, 95% (-15.41, 7.01)*, *P* = 0.46*<br><br>Interpretation of direction: Total score not reported. Higher score indicates greater number of patient receiving prevention interventions.

Linde [44]	Use of the practice innovation	I1:<br>n = 61<br>*Multifaceted (Level 3)*<br><br>I2:<br>n = 70<br>*Multifaceted (Level 2)*<br><br>C:<br>n = 54<br>*Multifaceted (Level 1)*	% yes:<br>6.12%<br><br><br>% yes:<br>3.51%<br><br><br>% yes:<br>0.00%		% yes:<br>53.05%<br><br><br>% yes:<br>50.88%<br><br><br>% yes:<br>29.70%<br><br>T: 1 month	I1 versus C:<br>Baseline: Non-significant**<br>Follow-up: RR 1.77*, 95% (1.10, 2.85)*, *P* = 0.02*<br><br>I2 versus C<br>Baseline: Non-significant**<br>Follow-up: RR 1.74*, 95% (1.08, 2.78)*, *P* = 0.02*<br><br>I1 versus I2:<br>Baseline: Non-significant**<br>Follow-up: RR 1.02*, 95% (0.73, 1.42)*, *P* = 0.91*<br><br>Interpretation of direction: Higher percentage indicates greater use.
Manias, et al. [48]	Manage pain using non-pharm-acological activities	I:<br>n = 32<br>*Multifaceted*<br><br>C:<br>n = 32<br>*No intervention*	n = 10<br><br><br><br>n = 12	n = 31<br><br><br><br>n = 12<br><br>T: immediate post-intervention	n = 29<br><br><br><br>n = 9<br><br>T: 3 months	I versus C<br>Baseline: Non-significant**<br>Interim: RR 2.58*, 95% CI (1.64, 4.06)*, *P* < 0.001*<br>Follow-up: RR: 3.22*, 95% CI (1.83, 5.67)*, *P* < 0.0001<br><br>Interpretation of direction: Higher percentage indicates greater use of non-pharmacological activities.
	Use of pain assessment tools	I:<br>n = 32<br>*Multifaceted*<br><br>C:<br>n = 32<br>*No intervention*	n = 15<br><br><br>n = 17	n = 30<br><br><br>n = 16<br><br><br>T: immediate post-intervention	n = 28<br><br><br>n = 15<br><br><br>T: 3 months	I versus C:<br>Baseline: Non-significant**<br>Interim: RR 1.88*, 95% CI (1.3, 2.68)*, *P* = 0.0006*<br>Follow-up: RR: 1.87* 95% CI (1.26, 2.76)*, *P* = 0.0002*<br><br>Interpretation of direction: Higher percentage indicates greater use of non-pharmacological activities.
* calculated using study data by review team, interim or follow-up only<br>** determined by review team<br>Abbreviations: I: Intervention; C: Control; T: Timeframe; CI: Confidence Interval, MD: Mean difference; AD: Absolute difference; RR: Relative Risk; OR: Odds Ratio						

Client Outcomes						
**Study**	**Outcome**	**Groups**	**Baseline**	**Interim**	**Follow-up**	**Overall Effect**
Dykes, et al. [49]	Fall rate per 1000 patient days	I:<br>n = 5160<br>*Multifaceted*<br><br>C:<br>n = 5104<br>*No intervention*	Rate per 1000 patient days:<br>5.56<br><br>Rate per 1000 patient days:<br>5.86		Rate per 1000 patient days:<br>3.15<br>95% CI:<br>(2.54, 3.90)<br><br>Rate per 1000 patient days:<br>5.86<br>95% CI:<br>(3.45, 5.06)<br><br>T: 6 months	I versus C<br>Baseline: *P* = 0.61<br>Follow-up: MD -1.03, 95% CI (-2.01, -0.57), *P* 0.04<br><br>Interpretation of direction: Higher the number greater number of falls.
	Falls with injury	I:<br>n = 5160<br>*Multifaceted*<br><br>C:<br>n = 5104<br>*Usual care*			No. yes:<br>12<br><br>No. yes:<br>14<br><br>T: 6 months	I versus C<br>Baseline: Not reported.<br>Follow-up: RR 1.15*, 95% CI (0.53, 2.49)*, *P* = 0.72*<br><br>Interpretation of direction: Higher the number greater number of falls.
Fan & Woolfrey [45]	Length of stay (minutes)	I:<br>n = 62<br>*Multifaceted*<br><br>C:<br>n = 62<br>*Usual care*			Mean:<br>73.0<br>95% CI:<br>(49.0, 93.0)<br><br>Mean:<br>79.9<br>95% CI:<br>(44.8, 109.8)<br><br>T: 2 weeks	I versus C<br>Baseline: Not reported.<br>Follow-up: MD -6.7, 95% CI (-20.9, 7.4), *P* = 0.349<br><br>Interpretation of direction: Higher the number of minutes the greater the length of stay.

Manias, et al. [48]	Pain intensity at rest (Visual Analog Scale, 0-10)	I:<br>n = 32<br>*Multifaceted*<br><br>C:<br>n = 32<br>*No intervention*	Mean:<br>5.58<br><br>Mean:<br>5.28	Mean:<br>4.40<br><br>Mean:<br>6.05<br><br>T: Immediate post-intervention	Mean:<br>3.14<br><br>Mean:<br>4.17<br><br>T: 3 months	I versus C<br>Baseline: MD -0.29, 95% CI (-1.40, 0.82), *P* = 0.608<br>Interim: MD: - 1.65, 95% CI (-2.79, -0.52), *P* = 0.004<br>Follow-up: MD: -1.03, 95% CI (-2.17, 0.09) *P* = 0.072<br><br>Interpretation of direction: Score of 0 to 10 (0 cm represents “no pain” and 10cm represents the “worst possible pain”). Higher the score the greater the pan intensity.
	Pain intensity on movement (Visual Analog Scale, 0-10)	I:<br>n = 32<br>*Multifaceted*<br><br>C:<br>n = 32<br>*No intervention*	Mean:<br>7.16<br><br>Mean:<br>6.26	Mean:<br>5.27<br><br>Mean:<br>7.42<br><br>T: Immediate post-intervention	Mean:<br>3.75<br><br>Mean:<br>6.24<br><br>T: 3 months	I versus C<br>Baseline: MD -0.90, 95% CI (-1.97, 0.16), *P* = 0.097<br>Interim: MD - 2.15 units, 95% CI (-3.19, -1.11), *P* < 0.0001<br>Follow-up: MD -2.49, 95% CI (-3.54, -1.44), *P* < 0.0001<br><br>Interpretation of direction: Score of 0 to 10 (0 cm represents “no pain” and 10cm represents the “worst possible pain”). Higher the score the greater the pan intensity.
Middleton, et al. [36]	Death or dependency	I:<br>n = 558<br>*Multifaceted*<br><br>C:<br>n = 451<br>*Educational materials*			% yes:<br>42%<br><br>%yes:<br>58%<br><br>T: 39 months	I versus C<br>Baseline: Non-significant<br>Follow-up: RR: 0.72*, 95% CI (0.65, 0.84)*, *P* =0.002<br><br>Interpretation of direction: Higher the percentage the greater the functional dependence (scored on a scale from 0 to 6 where “0” equals no symptoms, and “5” equals severe disability and “6” equals death; disability = score of ≥ 2).

	Functional dependence (Barthel index ≥ 95)	I:<br>n = 558<br>*Multifaceted*<br><br>C:<br>n = 451<br>*Educational materials*			% yes:<br>69<br><br>%yes:<br>60<br><br>T: 39 months	I versus C<br>Baseline: Non-significant<br>Follow-up: RR: 1.15*, 95% CI (1.04, 1.27)*, *P* =0.07<br><br>Interpretation of direction: Higher the percentage the greater the functional dependence (scored on a scale from 0 to 6 where “0” equals no symptoms, and “5” equals severe disability and “6” equals death; disability = score of ≥ 2).
	Functional dependence (Barthel index ≥ 60)	I:<br>n = 558<br>*Multifaceted*<br><br>C:<br>n = 451<br>*Educational materials*			% yes:<br>92%<br><br>%yes:<br>90%<br><br>T: 39 months	I versus C<br>Baseline: Non-significant<br>Follow-up: RR: 1.02*, 95% CI (0.98, 1.06)*, *P* =0.44<br><br>Interpretation of direction: Higher the percentage the greater the functional dependence (scored on a scale from 0 to 6 where “0” equals no symptoms, and “5” equals severe disability and “6” equals death; disability = score of ≥ 2).
	SF-36 (physical component summary score)	I:<br>n = 558<br>*Multifaceted*<br><br>C:<br>n = 451<br>*Educational materials*			Mean (SD):<br>45.6 (10.2)<br><br>Mean (SD):<br>42.5 (10.2)<br><br>T: 39 months	I versus C<br>Baseline: Non-significant<br>Follow-up: MD 3.4, 95% CI (1.2, 5.5), *P* =0.002<br><br>Interpretation of direction: Total score not reported. Higher score indicates greater states of health and well-being.
	SF-36 (mental health component summary score)	I:<br>n = 558<br>*Multifaceted*<br><br>C:<br>n = 451<br>*Educational materials*			Mean (SD):<br>49.5 (10.9)<br><br>Mean (SD):<br>49.4 (10.6)<br><br>T: 39 months	I versus C<br>Baseline: Non-significant<br>Follow-up: MD 0.5, 95% CI (1.9, 2.8), *P* =0.69<br><br>Total score not reported. Higher score indicates greater states of health and well-being.

	Temperature during first 72 hours	I:<br>n = 603<br>*Multifaceted*<br><br>C:<br>n = 483<br>*Educational materials*			Mean (SD):<br>36.5 (0.27)<br><br>Mean (SD):<br>36.5 (0.30)<br><br>T: 39 months	I versus C<br>Baseline: Not reported<br>Follow-up: MD 0.09, 95% CI (0.04, 0.15), *P* =0.001<br><br>Interpretation of direction: Higher ºC indicates greater temperature.
	At least one temperature ≥ 37.5ºC in first 72 hours	I:<br>n = 603<br>*Multifaceted*<br><br>C:<br>n = 483<br>*Educational materials*			% yes:<br>17%<br><br>% yes:<br>27%	I versus C<br>Baseline: Non-significant<br>Follow-up: RR: 0.64*, 95% CI (0.51, 0.81)*, *P* = <0.0001<br><br>Interpretation of direction: Higher ºC indicates greater temperature.
	Glucose during first 72 hours	I:<br>n = 603<br>*Multifaceted*<br><br>C:<br>n = 483<br>*Educational materials*			Mean (SD):<br>6.8 (1.8)<br><br>Mean (SD):<br>7.0 (2.0)<br><br>T: 39 months	I versus C<br>Baseline: Not reported<br>Follow-up: MD 0.54, 95% CI (0.08, 1.01), *P* =0.02<br><br>Interpretation: Higher blood glucose (mmol/L), indicates greater blood glucose.
	Discharge diagnosis of aspiration pneumonia	I:<br>n = 603<br>*Multifaceted*<br><br>C:<br>n = 483<br>*Educational materials*			% yes:<br>2%<br><br>% yes:<br>3%	I versus C<br>Baseline: Not reported<br>Follow-up: RR 0.64*, 95% CI (0.30, 1.36)*, *P* =0.82<br><br>Interpretation of direction: Higher percentage indicated greater diagnoses of aspiration pneumonia.

	Length of stay (days)	I:<br>n = 603<br>*Multifaceted*<br><br>C:<br>n = 483<br>*Educational materials*			Mean (SD):<br>11.3 (10.3)<br><br>Mean (SD):<br>13.7 (12.7)<br><br>T: 39 months	I versus C<br>Baseline: Not reported<br>Follow-up: MD 1.5, 95% CI (-0.5, 3.5), *P* =0.144<br><br>Interpretation of direction: Higher the number of days the greater the length of stay.
Seers, et al. [41]	Current pain intensity at rest (0-10)	I:<br>n = 60<br>*Multifaceted*<br><br>C:<br>n = 60<br>*No intervention*	Mean (SD):<br>1.75 (2.24)<br><br>Mean (SD):<br>1.80 (2.19)		Mean (SD):<br>1.36 (1.99)<br><br>Mean (SD):<br>1.36 (1.85)<br><br>T: 3 months	I versus C<br>Baseline: Non-significant.<br>Follow-up: MD: 0.00*, 95% CI (-0.69, -0.69)*, *P* = 1.00*<br><br>Interpretation of direction: Total score ranges from 0-10 (scored on a 10-point scale, “0” is no pain and “10” is worst pain possible). Higher scores indicate greater pain.
	Current pain intensity on movement (0-10)	I:<br>n = 60<br>*Multifaceted*<br><br>C:<br>n = 60<br>*No intervention*	Mean (SD):<br>3.47 (2.85)<br><br>Mean (SD):<br>3.51 (2.52)		Mean (SD):<br>2.98 (2.69)<br><br>Mean (SD):<br>3.12 (2.95)<br><br>T: 3 months	I versus C<br>Baseline: Non-significant<br>Follow-up: MD -0.14*, 95% CI (-1.15 to 0.87)*, *P* = 0.79*<br><br>Interpretation of direction: Total score ranges from 0-10 (scored on a 10-point scale, “0” is no pain and “10” is worst pain possible). Higher scores indicate greater pain.
	Pain intensity at rest since surgery (0-10)	I:<br>n = 60<br>*Multifaceted*<br><br>C:<br>n = 60<br>*No intervention*	Mean (SD):<br>2.55 (2.73)<br><br>Mean (SD):<br>3.98 (2.71)		Mean (SD):<br>2.54 (2.39)<br><br>Mean (SD):<br>2.81 (1.98)<br><br>T: 3 months	I versus C<br>Baseline: *P* = 0.009<br>Follow-up: MD -0.27*, 95% CI (-1.06, 0.52)*, *P* = 0.50*<br><br>Interpretation of direction: Total score ranges from 0-10 (scored on a 10-point scale, “0” is no pain and “10” is worst pain possible). Higher scores indicate greater pain.

	Pain intensity on movement since surgery (0-10)	I:<br>n = 60<br>*Multifaceted*<br><br>C:<br>n = 60<br>*No intervention*	Mean (SD):<br>4.02 (2.75)<br><br><br>Mean (SD):<br>4.84 (2.63)		Mean (SD):<br>3.54 (2.46)<br><br>Mean (SD):<br>3.58 (2.8)<br><br>T: 3 months	I versus C<br>Baseline: Non-significant<br>Follow-up: MD -0.04*, 95% CI (-0.99, 0.91)*, *P* = 0.93*<br><br>Interpretation of direction: Total score ranges from 0-10 (scored on a 10-point scale, “0” is no pain and “10” is worst pain possible). Higher scores indicate greater pain.
	Worst pain intensity since surgery at rest (0-10)	I:<br>n = 60<br>*Multifaceted*<br><br>C:<br>n = 60<br>*No intervention*	Mean (SD):<br>4.80 (3.26)<br><br><br>Mean (SD):<br>6.10 (2.50)		Mean (SD):<br>4.61 (3.45)<br><br><br>Mean (SD):<br>5.46 (3.22)<br><br>T: 3 months	I versus C<br>Baseline: *P* = 0.023<br>Follow-up: MD -0.85*, 95% CI (-2.04, 0.34)*, *P* = 0.16*<br><br>Interpretation of direction: Total score ranges from 0-10 (scored on a 10-point scale, “0” is no pain and “10” is worst pain possible). Higher scores indicate greater pain.
	Worst pain intensity since surgery on movement (0-10)	I:<br>n = 60<br>*Multifaceted*<br><br>C:<br>n = 60<br>*No intervention*	Mean (SD):<br>5.69 (3.06)<br><br><br>Mean (SD):<br>6.40 (2.67)		Mean (SD):<br>5.46 (3.11)<br><br><br>Mean (SD):<br>4.97 (3.34)<br><br>T: 3 months	I versus C<br>Baseline: Non-significant<br>Follow-up: MD 0.49*, 95% CI* (-0.66, 1.64)*, *P* = 0.41<br><br>Total score ranges from 0-10 (scored on a 10-point scale, “0” is no pain and “10” is worst pain possible). Higher scores indicate greater pain.
Sulch, et al. [47]	Length of stay (days)	I:<br>n = 76<br>*Multifaceted*<br><br>C:<br>n = 76<br>*Usual care*			Mean (SD):<br>50 (19)<br><br>Mean (SD):<br>45 (23)<br><br>T: 3 months	I versus C<br>Baseline: Not reported<br>Follow-up: MD 5, 95% CI, (-14.0, 24.0)<br><br>Interpretation of direction: Higher the number of days the greater the length of stay.

Titler, et al. [38]	Pain intensity during the first 24 hours of admission (0-10)	I: *Multifaceted*<br><br>C: *Educational materials*			T: 1 year	I versus C<br>Baseline: Not reported<br>Follow-up: MD -2.5, *P* < 0.0001<br><br>Interpretation of direction: Total score ranges from 0-10 [scored as 0 (no pain) to 10 (worst pain)]. Higher scores indicate greater pain. .
	Pain intensity over 72 hours of admission (0-10)	I: *Multifaceted*<br><br>C: *Educational materials*			T: 1 year	I versus C<br>Baseline: Not reported<br>Follow-up: MD: -1.5, *P* < 0.0001<br><br>Interpretation of direction: Total score ranges from 0-10 [scored as 0 (no pain) to 10 (worst pain)]. Higher scores indicate greater pain. .
Wesorick, et al. [46]	Blood glucose In-range	I: n = 453 *Multifaceted*<br><br>C: n = 391 *No intervention*			% yes: 17.0%<br><br>% yes: 16.9%<br><br>T: Not reported	I versus C:<br>Baseline: Not applicable<br>Follow-up: OR 1.08, 95% CI (0.74, 1.58), *P* = 0.68<br><br>Interpretation of direction: Higher percentage indicates greater blood glucose within range.
	Hyperglycemic	I: n = 453 *Multifaceted*<br><br>C: n = 391 *No intervention*			% yes: 63.8%<br><br>% yes: 63.4%<br><br>T: Not reported	I versus C:<br>Baseline: Not applicable<br>Follow-up: OR 0.95, 95% CI (0.71, 1.28), *P* = 0.74<br><br>Interpretation of direction: Higher percentage indicates greater hyperglycemia.
	Severely Hyperglycemic	I: n = 453 *Multifaceted*<br><br>C: n = 391 *No intervention*			% yes: 48.3%<br><br>% yes: 45.0%<br><br>T: Not reported	I versus C:<br>Baseline: Not applicable<br>Follow-up: OR 1.10, 95% CI (0.82, 1.47), *P* = 0.52<br><br>Interpretation of direction: Higher percentage indicates greater severe hyperglycemia.

	Hypoglycemic	I: n = 453<br>*Multifaceted*<br><br>C: n = 391<br>*No intervention*			yes: 5.1%<br><br>% yes: 9.2%<br><br>T: Not reported	I versus C:<br>Baseline: Not applicable<br>Follow-up: OR 0.48, 95% CI (0.27, 0.85), *P* = 0.01<br><br>Interpretation of direction: Higher percentage indicates greater hypoglycemia
	Severely Hypoglycemic	I: n = 453<br>*Multifaceted*<br><br>C: n = 391<br>*No intervention*			% yes: 2.9%<br><br>% yes: 3.8%<br><br>T: Not reported	I versus C:<br>Baseline: Not applicable<br>Follow-up: OR 0.97, 95% CI (0.29, 1.44), *P* = 0.28<br><br>Interpretation of direction: Higher percentage indicates greater severe hypoglycemia

* calculated using study data by review team, interim or follow-up only
Abbreviations: I: Intervention; C: Control; T: Timeframe; CI: Confidence Interval, MD: Mean difference; AD: Absolute difference; RR: Relative Risk; OR: Odds Ratio

References

32. Daly M, Kermode S, Reilly D. Evaluation of clinical practice improvement programs for nurses for the management of alcohol withdrawal in hospitals. Contemp Nurse. 2009;31(2):98-107.

33. Kirshbaum M. Translation to practice: A randomised, controlled study of an evidence-based booklet for breast-care nurses in the United Kingdom. Worldviews Evid Based Nurs. 2008;5(2):60-74.

34. Girouard S. The role of the clinical specialist as change agent: An experiment in preoperative teaching. Int J Nurs Stud. 1978;15(2):57-65.

35. Tsai Sl. The effects of a research utilization in-service program on nurses. Int J Nurs Stud. 2003;40(2):105-13.

36. Middleton S, McElduff P, Ward J, Grimshaw JM, Dale S, D'Este C, et al. Implementation of evidence-based treatment protocols to manage fever, hyperglycaemia, and swallowing dysfunction in acute stroke (QASC): A cluster randomised controlled trial. Lancet. 2011;378(9804):1699-706.

37. Melnyk BM, Bullock T, McGrath J, Jacobson D, Kelly S, Baba L. Translating the evidence-based NICU COPE program for parents of premature infants into clinical practice: impact on nurses' evidence-based practice and lessons learned. J Perinat Neonatal Nurs. 2010;24(1):74-80.

38. Titler M. Translating research into practice. Am J Nurs. 2007;107(6):26-33.

39. Day T, Wainwright SP, Wilson-Barnett J. An evaluation of a teaching intervention to improve the practice of endotracheal suctioning in intensive care units. J Clin Nurs. 2001;10(5):682-96.

40. Hyndman KJ. An evaluation of a dissemination intervention to enhance registered nurses' use of clinical practice guidelines related to tobacco reduction University of British Columbia (Canada); 2005.

41. Seers K, Crichton N, Carroll D, Richards S, Saunders T. Evidence-based postoperative pain management in nursing: Is a randomized-controlled trial the most appropriate design? J Nurs Manag. 2004;12(3):183-93.

42. Tranmer JE, Lochhaus-Gerlach J, Lam M. The effect of staff nurse participation in a clinical nursing research project on attitude towards, access to, support of and use of research in the acute care setting. Can J Nurs Leadersh. 2002;15(1):18-26.

43. Lewicki LJ. Diffusion of pressure ulcer guidelines: Testing an intervention Case Western Reserve University (Health Sciences); 1997.

44. Linde BJ. The effectiveness of three interventions to increase research utilization among practicing nurses University of Michigan; 1989.

45. Fan J, Woolfrey K. The effect of triage-applied Ottawa ankle rules on the length of stay in a Canadian urgent care department: A randomized controlled trial. Acad Emerg Med. 2006;13(2):153-7.

46. Wesorick DH, Grunawalt J, Kuhn L, Rogers MAM, Gianchandani R. Effects of an educational program and a standardized insulin order form on glycemic outcomes in non-critically ill hospitalized patients. J Hosp Med. 2010;5(8):438-45.

47. Sulch D, Perez I, Melbourn A, Kalra L. Randomized controlled trial of integrated (managed) care pathway for stroke rehabilitation. Stroke. 2000;31(8):1929-34.

48. Manias E, Gibson SJ, Finch S. Testing an educational nursing intervention for pain assessment and management in older people. Pain Med. 2011;12(8):1199-215.

49. Dykes PC, Carroll DL, Hurley A, Lipsitz S, Benoit A, Chang F, et al. Fall prevention in acute care hospitals: A randomized trial. JAMA. 2010;304(17):1912-8.

60. Wallen GR, Mitchell SA, Melnyk B, Fineout-Overholt E, Miller-Davis C, Yates J, et al. Implementing evidence-based practice: Effectiveness of a structured multifaceted mentorship programme. J Adv Nurs. 2010;66(12):2761-71.
